# Imidazo[2,1-*b*][1,3]thiazine Derivatives as Potential Modulators of Alpha-Synuclein Amyloid Aggregation

**DOI:** 10.1021/acschemneuro.4c00451

**Published:** 2024-11-27

**Authors:** Indrė Misiu̅naitė, Kamilė Mikalauskaitė, Martyna Paulauskaitė, Ru̅ta Sniečkutė, Vytautas Smirnovas, Algirdas Brukštus, Mantas Žiaunys, Ieva Žutautė

**Affiliations:** † Institute of Chemistry, Faculty of Chemistry and Geosciences, Vilnius University, Naugarduko st. 24, Vilnius LT-03225, Lithuania; ‡ Institute of Biotechnology, Life Sciences Center, Vilnius University, Saulėtekio al. 7, Vilnius LT-10257, Lithuania

**Keywords:** alkynes, Au catalysis, cyclization, imidazoles, alpha-synuclein, amyloid aggregation

## Abstract

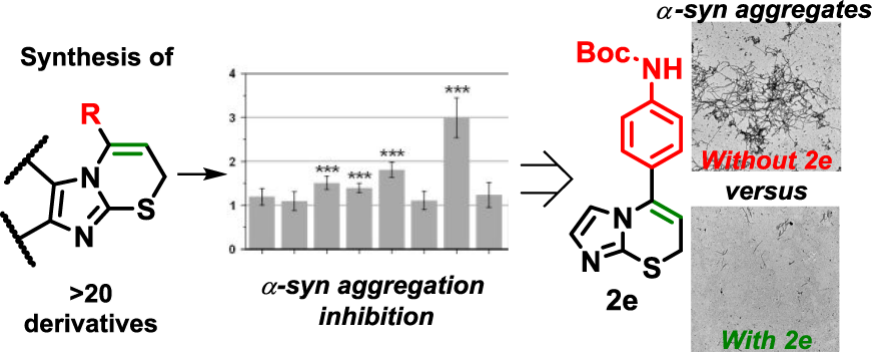

Insoluble amyloid fibrils accumulate in the intercellular spaces of organs and tissues, leading to various amyloidosis-related disorders in the human body. Specifically, Parkinson’s disease is associated with the aggregation of alpha-synuclein. However, current treatments for Parkinson’s primarily focus on managing motor symptoms and slowing disease progression. Efforts to prevent and halt the progression of these diseases involve the search for small molecular compounds. In this work, we synthesized imidazo[2,1-*b*][1,3]thiazines in an atom-economic way by cyclization of 2-alkynylthioimidazoles using 10% AuCl as the catalyst. We identified several compounds with specific functional groups capable of both inhibiting the aggregation of alpha-synuclein and redirecting the fibril formation pathway. The investigation into how these substances function revealed that imidazo[2,1-*b*][1,3]thiazine derivatives can influence alpha-synuclein aggregation in several ways. They not only inhibit the primary nucleation process and maintain a balance toward nonaggregated protein states but also stabilize smaller oligomeric species of alpha-synuclein and cause the formation of fibrils with unique structures and forms. These imidazo[2,1-*b*][1,3]thiazines could potentially be used in developing highly efficient, small molecular weight protein aggregation inhibitors.

## Introduction

Amyloid protein aggregation in the form of insoluble fibrils is associated with multiple widespread and incurable disorders, such as Alzheimer’s or Parkinson’s disease.^1,2^ More than 30 different proteins and peptides have been identified, whose cytotoxic aggregates accumulate in the intercellular spaces of organs and tissues.^1^ Despite decades of research and enormous effort in deciphering the amyloid aggregation process, there are still very few known treatment modalities,^3,4^ and the number of affected individuals is projected to increase further in the foreseeable future.^5,6^ Efforts to prevent and halt the progression of these diseases involve the search for various types of inhibitors, which range from small molecular weight compounds^7^ to monoclonal antibodies^8^ or complex drug mixtures.^9,10^

In the case of small molecules, a number of different class compounds have been identified as having antiamyloid potential by either directly inhibiting the fibril formation process or targeting factors that create conditions suitable for amyloid aggregation. These compounds include various polyphenols (gallic acid,^11^ epigallocatechin-3-gallate^12^), alkaloids (tolserine,^13^ galantamine^14^), terpenoids (1.8-cineole,^15^ α-pinene^16^), and polyketides (hispidin^17^). Several small molecular weight compounds have progressed to clinical trials, such as Anleb138b and NPT100-18A.^18^ Anleb138b has been found to inhibit alpha-synuclein (α-syn) oligomer formation,^19^ while NPT100-18A interferes with α-syn aggregation by displacing the protein from membranes.^20^ Minzasolmin, currently in Phase 2 clinical trials, stands out as one of the most promising potential disease-modifying therapeutics for Parkinson’s disease.^21^ Biophysical evaluations revealed that this compound acts early in the aggregation process by displacing membrane-bound oligomeric α-syn and returning it to a monomeric form, thus preventing the formation of larger protein aggregates and eventually Lewy bodies.^21^ However, despite hundreds of such potential antiamyloid compounds, the success rate in clinical trials remains very low, with most molecules failing at the initial phases.^22^

To expand the list of potential amyloid aggregation inhibitors, this work was dedicated to analyzing the activity of a previously unexplored small molecular weight compound group – imidazo[2,1-*b*][1,3]thiazines. These molecules are synthetically novel and have not been observed in nature, which has limited extensive investigations of their properties. Despite no prior research on their antiamyloid potential, it has been shown that compounds bearing a 1,3-thiazine moiety exhibit a wide range of biological activities,^23^ including antimicrobial, anti-inflammatory, antidiabetic, analgetic, and anticancer properties. To evaluate their possible influence on amyloid fibril formation, we synthesized 21 compounds and examined their effect on the aggregation process of α-syn – an intrinsically disordered protein related to the onset and progression of Parkinson’s disease.^24^

We discovered that some of the imidazo[2,1-*b*][1,3]thiazine derivatives possessed strong antiamyloid properties, which resulted in significantly longer aggregation lag phases. The inhibitor compounds also redirected the aggregation pathway, causing the formation of fibrils with different secondary structures and morphologies. Additionally, the presence of the imidazo[2,1-*b*][1,3]thiazine framework also shifted the α-syn monomer-fibril equilibrium toward the nonaggregated state. Together with previous reports of various positive biological activities, these results indicate that imidazo[2,1-*b*][1,3]thiazine derivatives are promising candidates for both synthetic and pharmaceutical applications.

## Results and Discussions

### Compound Synthesis

The synthesis of imidazo[2,1-*b*][1,3]thiazines **2** was carried out under the conditions established in our previous work.^25^ Various functionalized alkynes were selected as starting materials for the gold(I) chloride-promoted nucleophilic closure reaction in a microwave (MW) synthesizer. The synthesis of 2-alkynylthioimidazoles **1** typically began with the Sonogashira cross-coupling reaction of aryl iodides, followed by bromination of the hydroxyl group, and was concluded with nucleophilic substitution with the desired thioimidazole (see the Supporting Information). However, using 4-iodoaniline as the starting compound, the Sonogashira reaction did not proceed as expected. Therefore, a *tert*-butyloxycarbonyl (*Boc*) protective group was introduced into the molecule, allowing for selective alkylation and the Sonogashira reaction. The synthesis of imidazo[2,1-*b*][1,3]thiazines was generally carried out in a MW synthesizer at 50 °C for 140 min. Adjustments to these conditions were made, depending on the thioimidazole moiety in the starting material (Table 1). For instance, 2-alkynylthio-4,5-dimethylimidazoles **1g,h** required higher reaction temperatures to fully convert the starting materials while maintaining similar reaction time to those of compounds **1a**–**c**,**f** (Table 1, entries 1–3, 6), and the cyclization of compound **1e** (Table 1, entry 5) required a prolonged reaction time of up to 12.3 h. Compounds **2a**, **2c**, **2d**, **2g**, and **2i** (Table 1, entries 1, 3–4, 7, 9) were synthesized and isolated in our previous work.^25^ To evaluate the impact of the protecting group on α-syn aggregation, the *Boc* group was removed from compound **2j** (Table 1, entry 11). The deprotection was carried out under acidic conditions using TFA in DCM, resulting in compound **3** with a 61% yield.

**Table 1:**
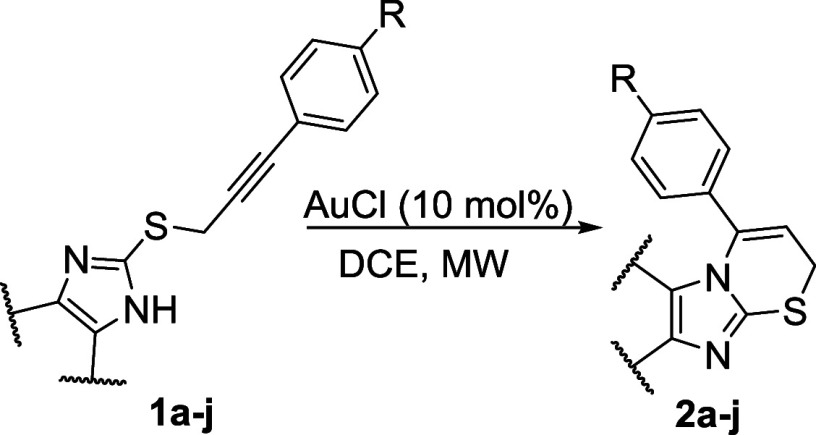

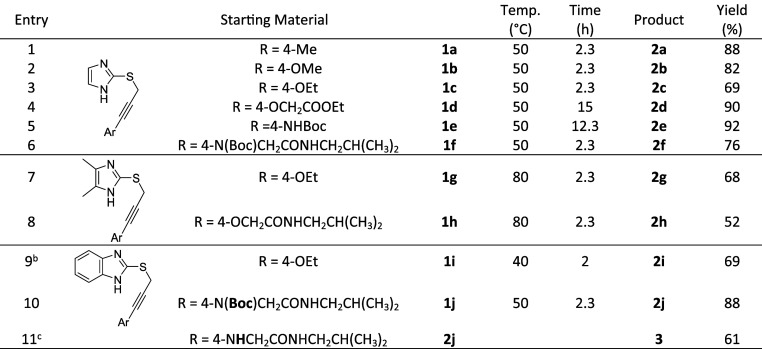
Au(I)Cl Initiated Cyclization Reactions of Various 2-Alkynylthioimidazoles^a^^b^^c^

a In an MW vial, compound **1** (from 50 mg to 200 mg, 0.17–0.45 mmol) was dissolved in 5 mL of DCE, and AuCl (10 mol %) was added under an argon atmosphere. The sealed vial was subjected to microwave irradiation until full conversion of the starting material.

b Substrate **1i** (80 mg, 0.30 mmol) was dissolved in 5 mL of DCM, then 10 mol % of AuCl was added, and the reaction mixture was refluxed under an argon atmosphere.

c Deprotection conditions: to the solution of **2j** (140 mg, 0,28 mmol, 1.0 equiv) in DCM (5 mL), TFA (1 mL) was added dropwise. The reaction mixture was stirred at room temperature overnight until full conversion of the starting material.

To broaden the scope of the compounds for investigation, ester **2d** was modified by substituting it with an amide group. This modification can be achieved from either an ester or an acid. To streamline the process and minimize the number of reaction steps, reactions involving ester **2d** were prioritized. According to the literature,^26^ amides can be synthesized from esters in ethanol using an appropriate amine. However, this method yielded only 33% of the desired amide. Another method suggested that esters and amines could react in DMF at 35–40 °C;^27^ however, after 5 days of heating, only a small amount of product was detected by TLC, leading to the termination of this reaction. Subsequent attempts to synthesize amides using excess amine as both a reagent and solvent also failed. Consequently, ester **2d** was hydrolyzed to its corresponding acid **4**, and a more sophisticated approach was employed using EDCI (1-ethyl-3-(3-dimethylaminopropyl)carbodiimide) and HOBt (1-hydroxybenzotriazole)^28^ as coupling reagents. Although the yield of amide formation with isobutylamine was modest, superior yields exceeding 70% were achieved with the corresponding amines (Table 2). Notably, compound **2h** was synthesized from the corresponding 2-alkynylthio-4,5-dimethylimidazole. Both methods are viable, depending on whether the goal is to modify an existing compound rapidly or to obtain a specific target compound.

**Table 2:**
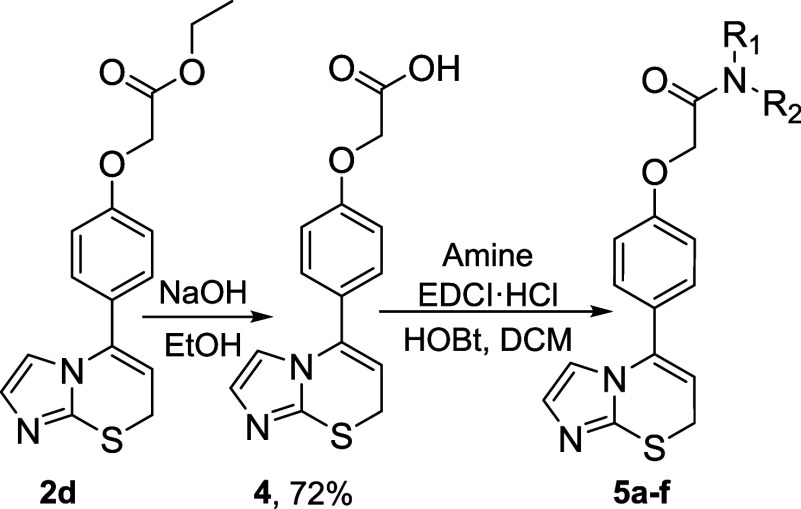

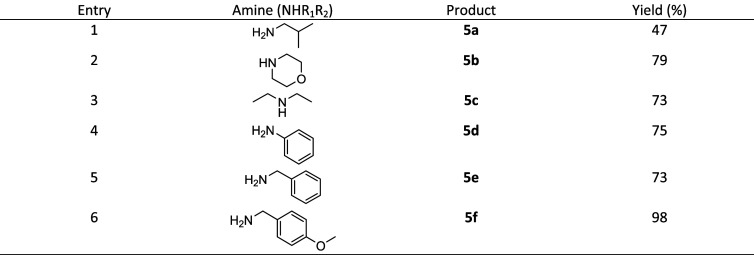
Synthesis of Amides **5** from Ester **2d**^a^

a Reaction condition for acid **4**: into a solution of **2d** (883.2 mg, 2.79 mmol, 1.0 equiv) in 20 mL of EtOH, NaOH (111.7 mg, 2.79 mmol, 1.0 equiv) was added. The reaction mixture was stirred at room temperature overnight. Reaction conditions for amides **5**: into a solution of compound **4** (50 mg, 0.17 mmol, 1 equiv) in 5 mL of DCM, EDCI·HCl (49.9 mg, 0.26 mmol, 1.5 equiv), HOBt (35.2 mg, 0.26 mmol, 1.5 equiv), and appropriate amine (0.21 mmol, 1.2 equiv) were added. The reaction mixture was stirred at room temperature overnight.

**Scheme 1:**
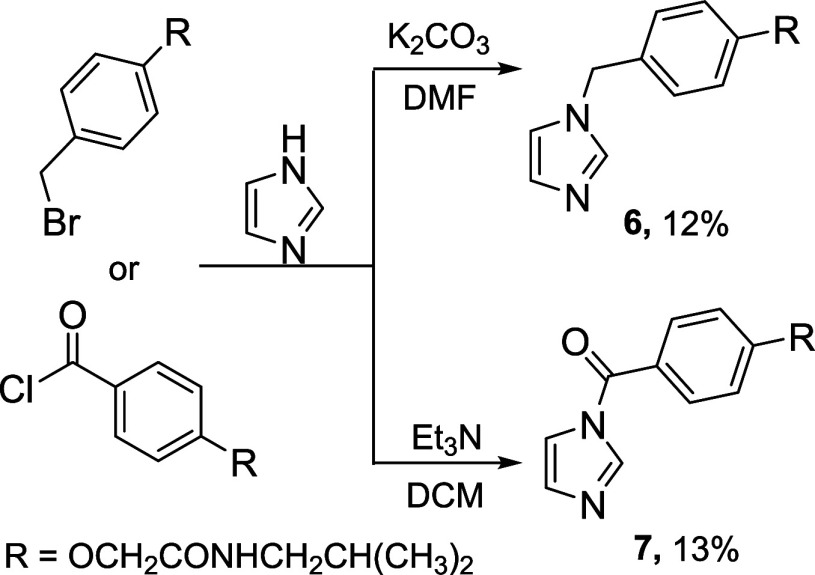
Synthesis of Compounds **6** and **7**

To investigate the necessity of the imidazothiazine ring for α-syn aggregation, it was decided to synthesize compounds **6** and **7** while retaining the imidazole ring and maintaining the same distance to the functional group as in the imidazothiazine **5a**. The mentioned compounds were obtained under classical alkylation and acylation conditions (Scheme 1). These molecules exhibit structural similarities to oxindoles, which have been studied for their effects on amyloidogenic protein aggregation by Kimura and colleagues.^29^

### Alpha-Synuclein Aggregation Inhibition

**Figure 1:**
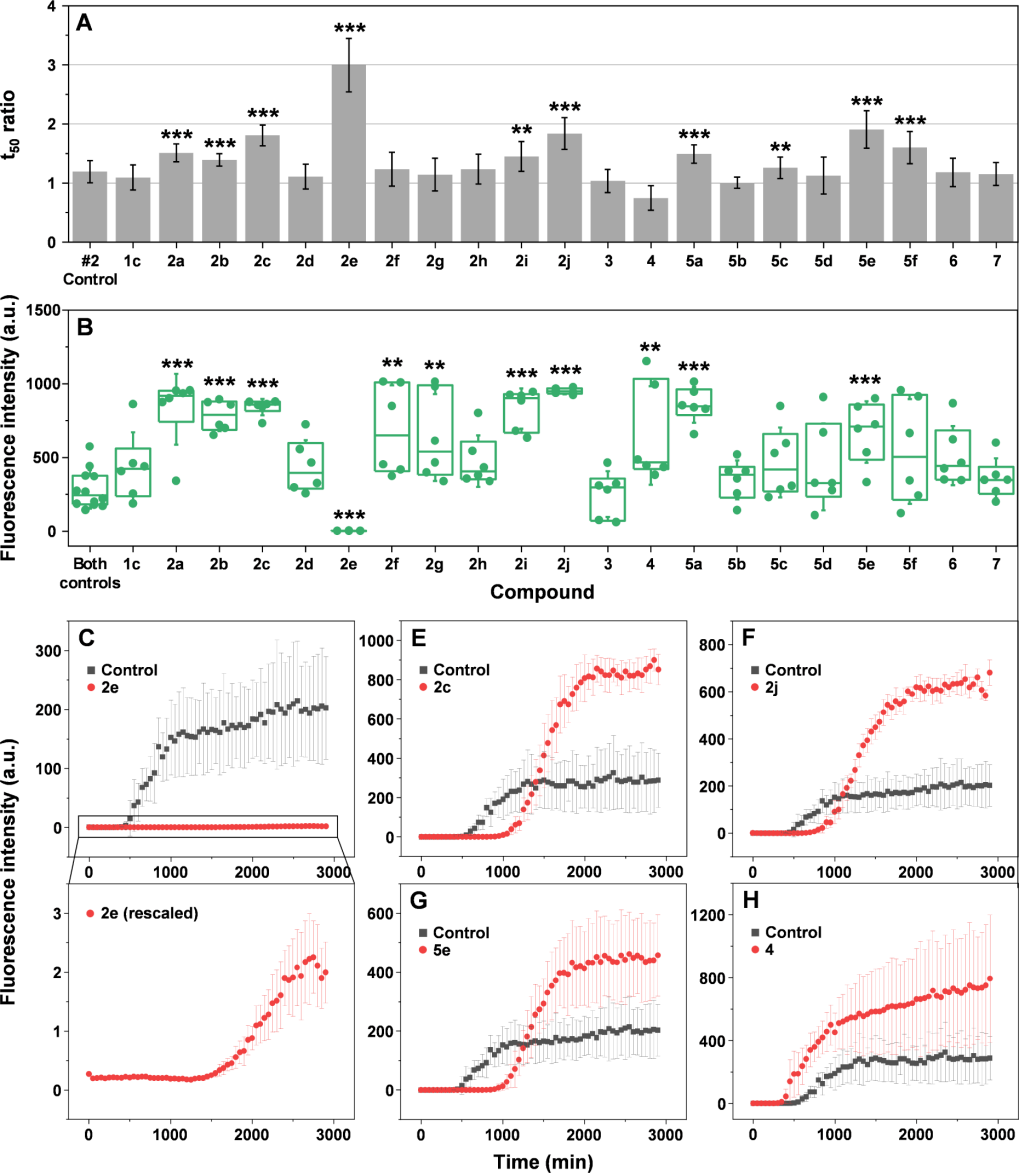
α-Syn aggregation kinetics in the presence of different imidazothiazine derivatives. (A) Aggregation half-time (*t*_50_) ratios (sample *t*_50_ values divided by control *t*_50_ values) of α-syn samples containing equimolar concentrations of different compounds (100 μM, *n* = 6, error bars are combined standard deviations). (B) End-point sample ThT fluorescence intensity value distribution (*n* = 6, box plots indicate the interquartile range, error bars are for one standard deviation). Significant differences were determined using ANOVA Bonferroni means comparison, ** *p* < 0.01, *** *p* < 0.001. (C–H). Rescaled images of kinetics with compound **2e** are shown below in panel C. Example kinetic curves of α-syn aggregation in the presence of compounds that inhibit or enhance the reaction.

The aggregation-inhibiting potential of the 18 synthesized imidazo[2,1-*b*][1,3]thiazine derivatives was determined (Figure S1) was examined by combining them with an equimolar concentration of α-syn and monitoring the formation of amyloid fibrils (Figure 1). During this research, the main attention was focused on substituents to the condensed heterocyclic system and additional functional groups in the phenyl *para* position of 5-arylimidazo[2,1-*b*][1,3]thiazines. First, the influence of substituents on the imidazole ring on α-syn aggregation was investigated. Imidazo[2,1-*b*][1,3]thiazine **2c** and benzimidazo[2,1-*b*][1,3]thiazine **2i** resulted in aggregation curves with significantly higher *t*_50_ values (Bonferroni means comparison, *n* = 6, *p* < 0.01), while for 2,3-dimethylimidazo[2,1-*b*][1,3]thiazine **2g**, the values were only slightly above the control but were not statistically significant (Figure 1A). Interestingly, all three molecules resulted in a significant increase in the sample end-point ThT fluorescence intensity values (Figure 1B), which would suggest an opposite effect to inhibition. According to the results, it was evident that the strongest aggregation reduction was achieved with compound **2c** bearing an unsubstituted imidazo[2,1-*b*][1,3]thiazine heterocyclic system. An additional comparison of compounds **2h** and **5a** confirmed the decrease in activity resulting from the introduction of methyl groups to the imidazole ring. 2,3-Dimethylimidazo[2,1-*b*][1,3]thiazine **2h** did not affect either the *t*_50_ values or the sample fluorescence intensity, while imidazo[2,1-*b*][1,3]thiazine **5a** displayed a significant effect on both parameters. However, it was considerably lower than that in the case of **2c**.

The crucial role in reducing α-syn aggregation of the thiazine ring was confirmed by tests with compounds **1c**, **6**, and **7**. All of them resulted in aggregation curves with similar *t*_50_ values. The 2-((3-(4-ethoxyphenyl)prop-2-yn-1-yl)thio)-1*H*-imidazole **1c**, the starting material of imidazothiazine **2c**, exhibited *t*_50_ values that were almost identical to the control. Compounds **6** and **7** affected α-syn aggregation significantly less than the corresponding imidazothiazine **5a** and were not significantly different from the control. Similar to the lack of influence on α-syn aggregation, these three compounds also did not cause any end-point sample fluorescence value deviations from the control samples (Figure 1B). From these results, it is evident that the imidazothiazine fragment forms an appropriate angle for the aryl substituent in the molecule and also may take part in interaction with the protein.

Next, the impact of the R group at the *para* position on the phenyl substituents in 5-arylimidazo[2,1-*b*][1,3]thiazines was examined. Even a single carbon difference had a notable outcome on α-syn aggregation. Comparing compound **2c**, with an −OEt group, and compound **2b**, with an −OMe group, revealed that a shorter chain decreased the average aggregation half-time ratio from 1.75 to 1.3. Compound **2a** differed from compound **2b** only in having a −Me group instead of an −OMe. This small structural change did not significantly impact α-syn aggregation reduction as the average values were 1.5 and 1.3, respectively. Despite these differences, all three compounds (**2a**, **2b**, **2c**) resulted in a significant increase in both the reaction *t*_50_ values, as well as the end-point fluorescence intensities (Figure 1A,B). Additionally, the functionalization of the phenolic group with an ester moiety in compound **2d** showed minimal change in α-syn aggregation, with *t*_50_ and fluorescence intensity values being similar to those of the control. The corresponding acid **4** appeared to even enhance the aggregation process. However, the change was not statistically significant. Replacing the ester group with various amides (compounds **5a**, **5c**, and **5e**–**5f**) increased activity compared to compounds **2d** and **4**, while activity remained the same for compounds **5b** and **5d**. The best result was achieved with amide **5e**, which yielded an average half-time ratio of 1.8, while the introduction of an additional −OMe group in benzyl (amide **5f**) resulted in a similar activity to compound **2c**, with a half-time ratio of 1.6 (Figure 1A). Of all amides **5a**–**f**, only **5a** and **5e** caused significantly higher end-point fluorescence intensity values (Figure 1B).

Replacing the phenolic group with substituted anilines resulted in enhanced activity of the corresponding compounds. Compound **2e**, bearing only the *Boc* group on aniline, unexpectedly demonstrated the largest half-time ratio value (Figure 1A). Unlike all other inhibitors, **2e** caused a massive reduction in the sample end-point fluorescence intensity (Figure 1B). While the addition of the −CH_2_CONHCH_2_CH(CH_3_)_2_ group in compound **2f** did not enhance the *t*_50_ value as expected and even lowered it, compared to lose aniline **2e** and mimicked compound **5a**. Maintaining this additional group while changing the imidazole ring to benzimidazole **2j** resulted in an increased average half-time ratio of 1.8, similar to compounds **2c** and **5e**. The role of the protecting group in α-syn aggregation reduction was crucial, as the activity was completely diminished in the case of compound **3**.

In summary, 21 compounds were tested for their ability to inhibit α-syn aggregation. The highest half-time ratios were achieved with imidazo[2,1-*b*][1,3]thiazines **2e**, **5e**, and **2c** and benzimidazo[2,1-*b*][1,3]thiazines **2j** (Figure 1C–G), while acid **4** displayed a possible aggregation-enhancing effect (Figure 1H). The presence of the imidazo[2,1-*b*][1,3]thiazine fragment was crucial for improved protein aggregation reduction. Particularly, the best results were achieved with *Boc* protected aniline **2e**. However, the addition of extra groups to this structure led to a drastic drop in the efficiency. Nonetheless, the benzimidazole moiety helped to mitigate the impact of additional groups.

### Effect on Fibril Structure and Morphology

Despite the positive antiamyloid activity of the compounds, a majority of them yielded samples with a significantly higher ThT fluorescence intensity (Figure 1B). Since none of these compounds possess an intrinsic fluorescence capacity under the ThT-specific excitation and emission wavelengths (Figure S2), the observed phenomenon had two possible explanations. Either the compounds shifted the equilibrium between the native and aggregated states of α-syn in favor of amyloid fibrils, or the resulting structures had different ThT binding modes.^30^ We have previously observed a similar event, where antiamyloid compounds redirected α-syn aggregation into fibrils with different secondary structures,^31^ which, in turn, can affect their dye-binding properties.^32,33^

**Figure 2:**
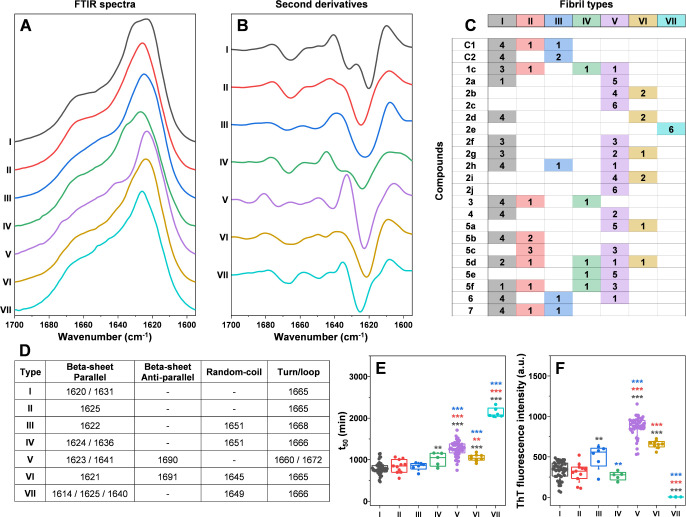
α-Syn aggregate secondary structure dependence on the presence of compounds. (A, B) Seven different α-syn fibril FTIR spectra (FTIR) and their second derivatives detected after aggregation with or without compounds. (C) Fibril type distribution in samples without (control #1, control #2) or with different compounds (color-coded boxes indicate the number of each specific type of fibril). (D) Different fibril FTIR spectra second-derivative minimum positions related to parallel beta-sheets, antiparallel beta sheets, random-coil, and turn/loop motifs. (E, F) *t*_50_ and end-point ThT fluorescence intensity value distribution of different fibril type aggregation curves. Significant differences were determined using ANOVA Bonferroni means comparison, ** *p* < 0.01, *** *p* < 0.001.

To examine this possibility, all samples (*n* = 6 for each compound) were tested using Fourier-transform infrared (FTIR) spectroscopy. The resulting spectra of the amide I region and their second derivatives were compared against each other and grouped based on similarities in their peak and minima positions (Figure 2A,B). In the case of samples from both control runs, the FTIR spectra were divided into three distinct types: I, II, and III (Figure 2C). Such random polymorphism has been shown in previous studies under similar conditions.^34^ Type I fibril FTIR spectra second derivatives contained two minima at 1631 and 1620 cm^–1^ (Figure 2D), associated with two different strengths of hydrogen bonding in the beta-sheet structure^35^ and a minimum at 1665 cm^–1^ (turn/loop motif). Type II displayed a single minimum related to beta-sheets at 1625 cm^–1^ and a minimum identical to type I at 1665 cm^–1^. Type III fibril FTIR derivatives had minima at 1622 cm^–1^ (beta-sheets), 1651 cm^–1^ (random-coil), and 1668 cm^–1^ (turn/loop motif).

When α-syn was aggregated in the presence of exploratory compounds, four additional types of FTIR spectra were observed. Type IV was only detected in one out of the six repeats for samples containing compounds **1c**, **3**, **5d**, **5e**, and **5f** (Figure 2C). Its second derivatives had minima at 1624 cm^–1^, with a shoulder at 1636 cm^–1^ (two different strength hydrogen bonding types in the beta-sheet structure) and minima at 1651 cm^–1^ (random-coil), 1666 cm^–1^ (turn/loop motif). Type V, on the other hand, was the most abundant spectral type detected among all of the samples (Figure 2C). It had minima related to beta-sheets at 1641 and 1623 cm^–1^, turn/loop motifs at 1660 and 1672 cm^–1^, as well as a minimum associated with antiparallel beta-sheets at 1690 cm^–1^ (Figure 2B,D). Type VI fibril FTIR spectra were detected only for a small number of samples containing **2b**, **2d**, **2g**, **2i**, **5a**, and **5d** compounds (Figure 2C). Their second derivatives had minima at 1621 cm^–1^ (beta-sheets), 1645 cm^–1^ (random-coil), 1665 cm^–1^ (turn/loop motif), and 1691 cm^–1^ (antiparallel beta-sheets). Finally, type VII spectra were observed only in the case of the strongest inhibitor compound **2e**. Their second derivatives had minima at 1625 cm^–1^ with a shoulder at 1614 cm^–1^ (two types of hydrogen bonding strength in the beta-sheet structure), 1640 cm^–1^ (weak beta-sheet hydrogen bonding), 1649 cm^–1^ (random-coil), and 1666 cm^–1^ (turn/loop motif).

Based on the FTIR spectral distribution, it was clear that imidazo[2,1-*b*][1,3]thiazine derivatives could influence the secondary structure of the formed aggregates. In some cases, such as with compounds **2c**, **2e**, and **2j**, the aggregation reaction was redirected to produce fibrils with a single FTIR spectrum across all six repeats. Interestingly, the compounds that increased *t*_50_ and/or fluorescence intensity were also, for the most part, the ones that promoted the formation of these four different secondary structures (Figures 1A and 2C). These findings suggest a possible correlation among the inhibitory effect, the resulting aggregate structures, and their ThT-binding ability.

In order to test this hypothesis, the sample aggregation *t*_50_ values and end-point fluorescence intensities were grouped based on their respective FTIR spectrum type. Surprisingly, the spread of data was minimal for almost all types, further supporting the aforementioned correlation. In the case of *t*_50_ value distributions (Figure 2E), samples resulting in type I–III FTIR spectra did not have any significant differences between them (ANOVA Bonferroni means comparison). Conversely, type V–VII samples all displayed significantly different *t*_50_ value distributions. The type IV sample *t*_50_ values were significantly different from those of only one of the three types detected in the control samples. A similar tendency was observed for the fluorescence intensity distributions (Figure 2F). Type V–VII samples were significantly different from either all or two of the three control sample types. However, unlike the *t*_50_ values, the three control samples also did not display nearly identical distributions, with type III having a mean value significantly higher than that of the type I samples. Finally, type IV sample fluorescence intensities were comparable to two of the three control samples and shared a dissimilarity to the type III samples.

Taken together, these data revealed that there was a strong correlation between the aggregate secondary structures and their formation time, as well as ThT-binding parameters. This could be achieved either by the compounds promoting the formation of certain fibril nuclei or by selectively inhibiting the assembly of type I–III aggregate nuclei. Considering that all samples with type IV–VII FTIR spectra had larger *t*_50_ values, the selective inhibition hypothesis seemed like the most likely explanation.

**Figure 3:**
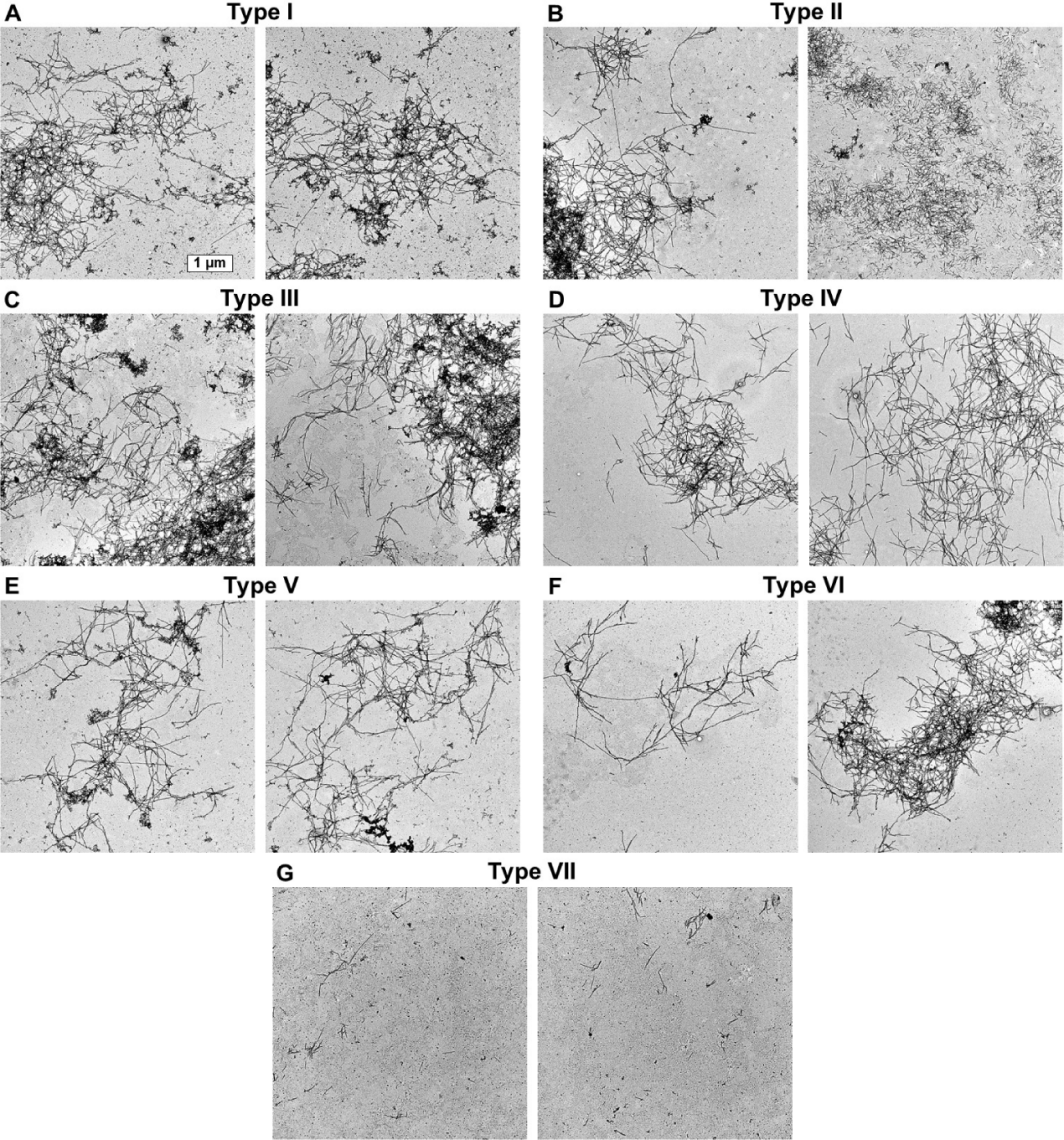
Electron microscopy (EM) images of α-syn aggregates with seven distinct FTIR spectra. (A–G) Two 4096 × 4096 pixel resolution representative images for each fibril type. The scale bar (presented in the top left image) is identical for all images.

Since different α-syn secondary structures are often an indicator of distinct fibril morphologies, the seven fibril types were further examined using electron microscopy (EM). Based on the results, four unique morphological aspects were observed. Type I, II, III, and VI fibrils displayed a high level of self-association, yielding large aggregate clusters (Figure 3A–C and F). Type IV and V fibrils, despite possessing similar lengths to the aforementioned types, were less prone toward such clump formation and associated into intertwined networks (Figure 3D,E). Type II aggregates, along with their tendency of cluster formation, also displayed an abundance of short (<1 μm) fibrils (Figure 3B, second image). Finally, type VII samples were composed of a low concentration of fibrillar structures and a relatively high amount of small amorphous aggregates or oligomeric species (Figure 3G).

These results were correlated with the previously described sample fluorescence intensity values. The type VII samples displayed the least amount of fibrillar structures, and they also pertained the lowest fluorescence intensities. Type I, II, III, and VI fibrils formed large aggregate clusters, which likely impeded the ability of ThT to bind on some parts of the structures. Due to this reason, the type V and VI fibrils, which formed intertwined networks, were also the ones with the highest ThT fluorescence intensity. These morphological distinctions indicate that the inhibitory compounds can affect α-syn fibril self-association tendencies and length as well as the appearance of oligomeric or amorphous structures and the resulting overall sample ThT fluorescence intensity.

### Mechanism of Inhibition

**Figure 4:**
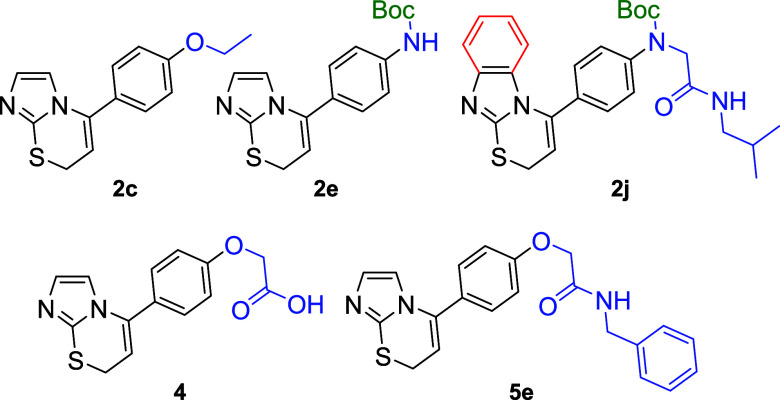
Structures of selected active compounds **2e**, **2c**, **2j**, **4**, and **5e**.

Despite these observations, two important questions remained: what is the mechanism of inhibition, and how do the imidazo[2,1-*b*][1,3]thiazine derivatives affect the monomer-fibril equilibrium. In order to gain deeper insight into the process and answer these questions, five compounds (**2c**, **2e**, **2j**, **4**, and **5e**) were selected for further study (Figure 4). According to the obtained experimental data, compound **2e** was chosen as the strongest inhibitor, and compounds **2c**, **2j**, and **5e** were selected as moderate inhibitors. Imidazothiazine **2c** represents the simplest active structure with an ethoxy substituent. Compound **2j** bears a benzimidazothiazine moiety with an amide substituent like imidazothiazine **5e**. Imidazotiazinylphenoxyacetic acid **4** was also additionally tested as the only compound that displayed a possible aggregation-enhancing activity. To determine which fibrillization step was influenced by these compounds, α-syn aggregation was carried out under different compound concentrations. The resulting aggregation curves were fit using a Boltzmann sigmoidal equation, and three parameters were calculated, which included the reaction lag time (affected by primary nucleation), apparent rate constant (affected by the rate of elongation, secondary nucleation, and fragmentation), and end-point fluorescence intensity (affected by aggregate structure and quantity).

**Figure 5:**
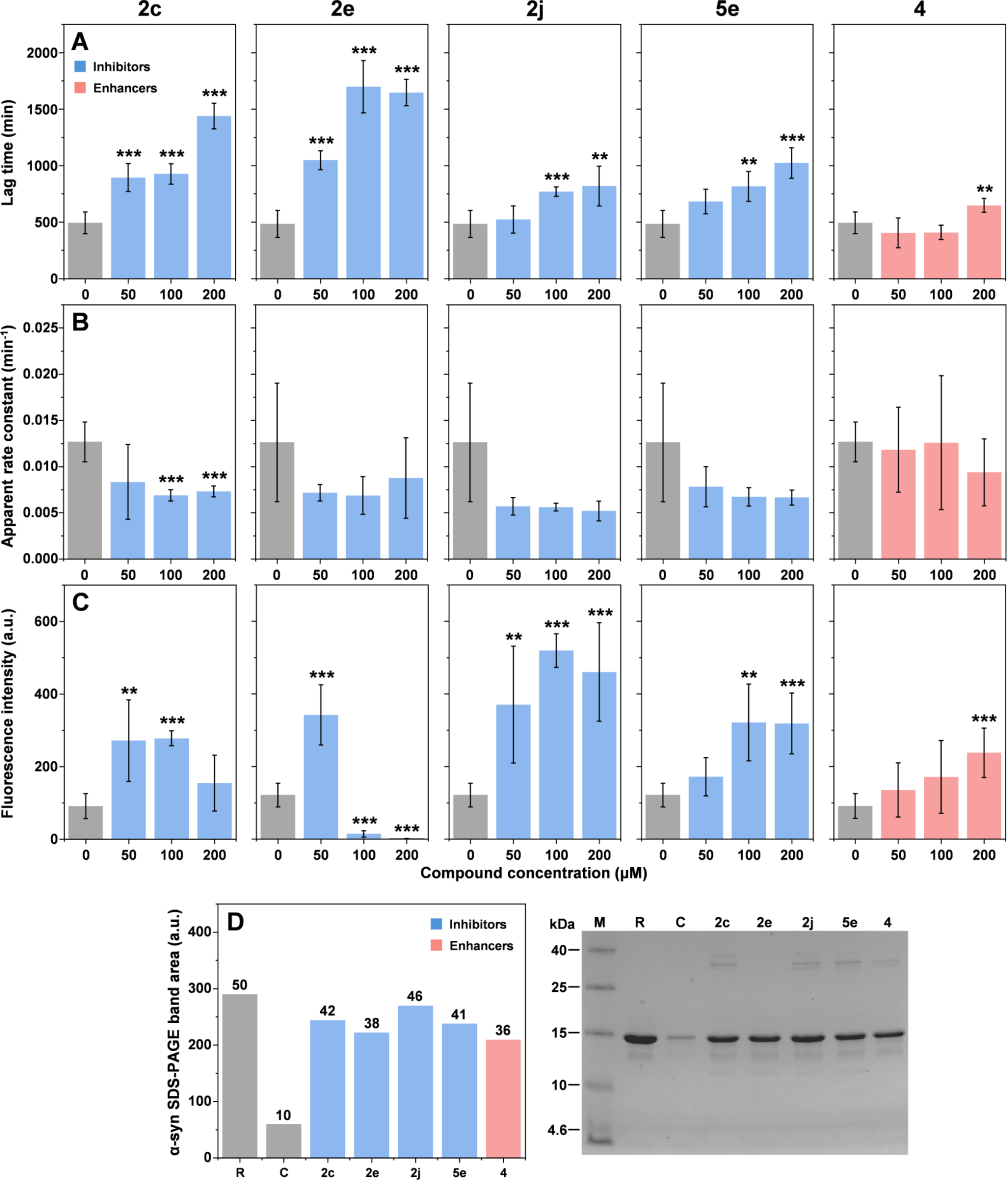
α-Syn aggregation kinetic parameter dependence on different concentrations of compounds. (A–C) Lag time, apparent rate constant, and fluorescence intensity value distribution of α-syn aggregation curves in the presence of different compound concentrations. Significant differences were determined using ANOVA Bonferroni means comparison, *n* = 6, ** *p* < 0.01, *** *p* < 0.001. (D) Sample supernatant SDS-PAGE gel and α-syn band area distribution, “R” – reference 50 μM monomeric α-syn sample, “C” – control fibril sample supernatant, and numbers indicate the calculated concentration of α-syn based on the reference band.

In the case of all four inhibitors, there was an increase in reaction lag times based on the concentration of the compounds (Figure 5A, **2c**, **2e**, **2j**, and **5e**). Interestingly, each compound displayed different tendencies in how the lag times changed. In the case of compound **2c**, both 50 and 100 μM concentrations resulted in an almost identical value, while 200 μM yielded a considerably higher level of inhibition. For compound **2e**, the inhibitory effect became saturated at 100 μM and a higher concentration did not result in any notable effect. Compound **2j** only displayed an inhibitory effect at 100 and 200 μM, which was also similar under both conditions. Out of the four inhibitors, **5e** demonstrated the most linear dependence between the aggregation lag time and compound concentration. The previously detected aggregation-enhancing compound **4** resulted in reduced reaction lag times under 50 and 100 μM concentrations. However, this effect was not statistically significant. Interestingly, 200 μM of the compound yielded a significantly higher reaction lag time, suggesting that it may act as an enhancer or inhibitor based on its concentration.

Examination of the reaction apparent rate constants (Figure 5B) revealed that all four inhibitors (**2c**, **2e**, **2j**, **5e**) induced a concentration-dependent decrease in their average value, where the 200 μM compound concentration samples displayed a 2-fold lower rate constant than the control. In the case of the possible enhancer, the average apparent rate constant decrease was not as intense. However, due to the large stochasticity of this parameter, statistically significant differences could only be determined for compound **2c**.

The end-point fluorescence data presented an even more complicated picture (Figure 5C). Compound **2c** resulted in an arc-shaped fluorescence intensity dependence on its concentration, with 50 and 100 μM condition samples having significantly higher average values, while 200 μM condition samples were similar to the control. In the case of **2e**, the fluorescence intensity was significantly higher under conditions with 50 μM compound, while both higher concentrations yielded samples where the ThT signal was almost completely quenched. Compound **2j** produced samples with highly variable fluorescence intensities, with all condition values within the margin of error. For compound **5e**, both 100 and 200 μM conditions resulted in an almost identical and significantly higher fluorescence intensity. The enhancer compound resulted in a linear dependence between its concentration and the average sample fluorescence intensity values. However, only the 200 μM condition samples were significantly different.

A possible explanation for these highly stochastic results is the previously shown correlation between fibril types and their fluorescence intensities. If lower compound concentrations are not sufficient to redirect all nuclei formation to a certain structure, then each condition results in a different distribution of fibril types, each with its own specific fluorescence intensity. To determine if the significant differences in end-point fluorescence intensity were purely related to distinct fibril structures or a shift in monomer–aggregate equilibrium, the highest compound concentration samples were centrifuged, and their supernatants were examined with SDS-PAGE. As two reference points, a 50 μM α-syn sample (Figure 5D, marked as “R”, 50 μM concentration was used to avoid signal overflow during gel analysis) and a control α-syn fibril sample supernatant (Figure 5D, marked as”C”) were used. The dyed gel was imaged and analyzed by using GelAnalyzer software.

In the case of the control fibril sample, the band was almost nondetectable, indicating an efficient conversion of the majority of α-syn into its aggregated state (90 μM out of 100 μM). Surprisingly, all tested compounds resulted in a similar residual concentration of α-syn regardless of the inhibitory effect or the end-point fluorescence values. In all cases, the nonaggregated α-syn composed approximately 40% of the total protein content in the samples, which was considerably higher than under conditions with no imidazo[2,1-*b*][1,3]thiazine derivatives. This result suggests that the fluorescence signal was almost entirely related to the types of fibrils that were formed and not due to their quantity. Another interesting aspect was the presence of bands between 25 and 40 kDa markers in samples with compounds **2c**, **2j**, **5e**, and **4**. Since this band is not observed in either the monomeric or control sample, it suggests that the compounds may have been capable of stabilizing dimeric and trimeric forms of α-syn.

### Compound Toxicity

**Figure 6:**
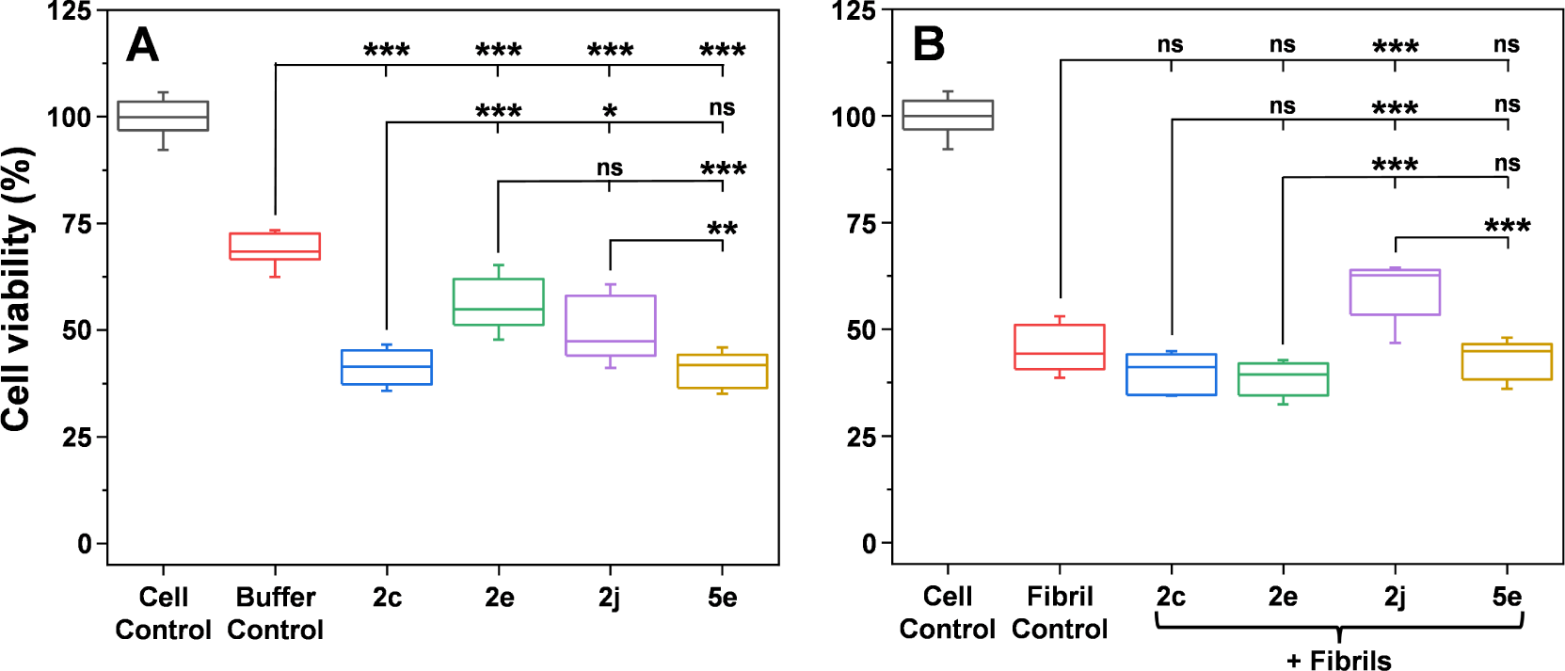
Effect of imidazo[2,1-*b*][1,3]thiazine derivatives on SH-SY5Y human neuroblastoma cells. MTT viability assay of the selected compounds (20 μM) in the absence (A) or presence (B) of α-syn amyloid fibrils (20 μM). For each condition, three independent assays were carried out (each with three sample repeats), and error bars represent one standard deviation (*n* = 9). One-way ANOVA Bonferroni means comparison was conducted to determine differences between samples (ns – not significant, * *p* < 0.05, ** *p* < 0.01, *** *p* < 0.001).

To determine whether the compounds with the highest antiaggregation effects can also counteract the cytotoxic effects of α-syn fibrils, an MTT assay was performed using SH-SY5Y human neuroblastoma cells. When 20 μM of each compound was introduced to the medium without α-syn aggregates, all of them caused a statistically significant reduction in cell viability (Figure 6A, *n* = 9, ANOVA Bonferroni means comparison). The chosen concentration of compounds for the cell viability assay was much higher than the concentration that would likely exist at target locations after compound administration. However, it was required to compare the relative toxicity of each compound, as lower concentrations do not significantly deviate from the buffer solution control (Figure S3).

The negative effect on cell viability was significantly greater for compounds **2c** ((41 ± 4)% cell viability) and **5e** ((41 ± 4)%) when compared to the buffer solution control ((69 ± 4)%). In contrast, the reduction in cell viability was less pronounced for compounds **2e** ((56 ± 6)%) and **2j** ((50 ± 7)%), with **2e** having the smallest impact. When the medium contained both α-syn fibrils and the compounds (Figure 6B), **2j** stood out by markedly reducing aggregate-induced cytotoxicity. The effects of compounds **2c** ((40 ± 4)%), **2e** ((38 ± 4)%), and **5e** ((43 ± 4)%) were similar to the fibril control ((45 ± 5)%), while neuroblastoma cell viability in the presence of **2j** was significantly higher ((59 ± 6)%). Given the compound’s **2j** dual effect in inhibiting aggregation and reducing cytotoxicity, these results suggest that this compound decreases not only the rate of aggregate formation but also the cytotoxic effects of aggregates themselves.

Considering all the aforementioned results, imidazo[2,1-*b*][1,3]thiazine derivatives appear to have multiple effects on α-syn aggregation. Not only do they inhibit primary nucleation and shift the monomer-fibril equilibrium toward the nonaggregated state, but they also can stabilize small oligomeric states of α-syn and redirect the aggregation reaction toward fibrils with distinct secondary structures and morphologies. It is important to note that the best results were achieved with imidazo[2,1-*b*][1,3]thiazines containing phenylcarbamate fragments (**2e**, **2j**). Further modification of the core structure of compound **2e**, resulting in the more substituted benzimidazo[2,1-*b*][1,3]thiazine **2j**, led to a statistically significant reduction in the cytotoxic effects of the aggregates. However, compound **2j** still negatively affected cell viability at relatively high concentrations. Therefore, future studies should focus on further structure modifications to improve compound’s antiamyloid activity and physicochemical properties as potential drug candidates while minimizing their cytotoxicity.

## Conclusions

In summary, we synthesized 18 compounds containing an imidazothiazine framework (**2a**–**j**, **3**, **4**, and **5a**–**f**) under mild conditions using AuCl as a catalyst. A diversity of functional groups was obtained by modifying the formed 5-arylimidazo[2,1-*b*][1,3]thiazines at their *para*-phenyl positions. The significance of the imidazo[2,1-*b*][1,3]thiazine framework was demonstrated through inhibition activity comparison with two synthesized model compounds, **6** and **7**. The highest half-time ratios were achieved with compounds **2e**, **2j**, **5e**, and **2c**, with the best results observed for *Boc*-protected aniline **2e**. However, further adding extra groups to the aniline or its deprotection led to a decreased efficiency. Nonetheless, the benzimidazole moiety helped mitigate the impact of additional groups. Based on the FTIR spectra distribution, it was evident that imidazo[2,1-*b*][1,3]thiazine derivatives influenced the secondary structure of the formed aggregates. The study of the inhibition mechanism revealed that imidazo[2,1-*b*][1,3]thiazine derivatives may have multiple effects on α-syn aggregation. These compounds not only inhibit primary nucleation and shift the monomer-fibril equilibrium toward the nonaggregated state but also stabilize the oligomeric states of α-syn and redirect the aggregation reaction toward fibrils with distinct secondary structures and morphologies. Additionally, one compound (**2j**) could reduce the cytotoxic effects of α-syn fibrils. These findings suggest that compounds containing the imidazo[2,1-*b*][1,3]thiazine framework could serve as potential α-syn aggregation inhibitors.

## Materials and Methods

### Compound Preparation

Detailed compound synthesis protocols and their NMR spectra are provided as Supporting Information. All reagents and solvents were dried commonly before use, according to standard procedures. Commercially available reagents were used without further purification, unless otherwise noted. Oxygen- and moisture-sensitive reactions were carried out under an argon atmosphere. ^1^H and ^13^C NMR spectra were recorded in deuterated solvents on a Bruker Ascened 400 MHz spectrometer. High-resolution mass spectra (HRMS) were recorded on an Agilent LC/MSD TOF mass spectrometer by electrospray ionization time-of-flight (ESI-TOF) reflection experiments. Infrared spectra were recorded on a PERKIN-ELMER 1000 FT-IR spectrometer with a UATR annex. Microwave (MW) irradiation reactions were carried out using a CEM Discover SP microwave synthesizer. Reactions were monitored by thin layer chromatography (TLC) carried out on 0.25 mm Merck silica plates (60 F_254_), using UV light as the visualizing agent and/or vanillin and heat as a developing agent. Column chromatography was performed with Kieselgel 60 (40–63 μm) silica gel. Melting points were measured on a Student SMP10 instrument and were uncorrected. Here are the general methods of the main reactions used for the synthesis of targeted compounds.

### Synthesis of Imidazo[2,1-*b*][1,3]thiazine Framework **2**

The solution of 2-alkynylthioimidazole **1** (1.0 equiv) in DCE was stirred under an argon atmosphere at room temperature in a microwave vial for 15 min, then AuCl (10 mol %) was added, and the sealed vial was subjected to microwave irradiation. The reaction was carried out using a dynamic method at constant temperature (four cycles: three times for 40 min and one time for 20 min). If the reaction was not completed during this time, the cycles were repeated. After reaction completion, monitored by TLC, the solvent was evaporated under reduced pressure, and the product **2** was isolated by column chromatography.

### Synthesis of Amides **5**

To the solution of acetic acid **4** (1.0 equiv) in DCM, EDCI·HCl (1.5 equiv), HOBt (1.5 equiv), and appropriate amine (1.2 equiv) were added. The reaction mixture was stirred at room temperature overnight. After reaction completion, monitored by TLC, the mixture was diluted with H_2_O and extracted with DCM. The combined organic layers were washed once with brine, dried with anhydrous Na_2_SO_4_, and concentrated under reduced pressure. The product **5** was isolated by column chromatography using the eluent CHCl_3_:CH_3_CN (4:1).

### Alpha-Synuclein Aggregation Assay

Alpha-synuclein was purified based on a previously described protocol,^36^ exchanged to PBS (pH 7.4), concentrated to 600 μM, and stored at −20 °C prior to use. The compounds were dissolved in 99.9% dimethyl sulfoxide (DMSO) to a final concentration of 10 mM. Thioflavin-T (ThT) powder (Sigma-Aldrich cat. no. T3516) was dissolved in Milli-Q H_2_O to a concentration of ∼11 mM and filtered through a 0.22 μm pore-size syringe filter. The exact concentration of the dye was determined by diluting a small aliquot (10 μL) to 1 mL of Milli-Q H_2_O and scanning its absorbance at 412 nm using a Shimadzu UV-1800 spectrophotometer. The final concentration of the ThT stock solution was then set to 10 mM. Both the compound and ThT stock solutions were used immediately after preparation, flash frozen, and stored at −20 °C under dark conditions to be used in further experimental procedures.

To conduct the aggregation assays, α-syn, compound, ThT, 10× PBS, and 1× PBS solutions were combined to result in final reaction solutions containing 100 μM α-syn, 100 μM compound, 100 μM ThT, and 1× PBS. The protein concentration of 100 μM was chosen to reduce the aggregation reaction stochasticity.^33^ For the initial assessment, both the dye and compound concentrations were set to be equimolar to α-syn. Control solutions were supplemented with 99.9% DMSO instead of the compound solution. The reaction solutions were then distributed to 96-well nonbinding half-area plates (Fisher Scientific, cat. no 3881, 100 μL volume, 6 repeats for each condition, each well contained one 3 mm glass bead) in an alternating pattern to account for uneven temperature distribution.^37^ The aggregation reactions were monitored by incubating the plates in a ClarioStar Plus plate reader at 37 °C with 600 rpm agitation between readings. ThT fluorescence measurements were taken every 5 min using 440 nm excitation and 480 nm emission wavelengths. For aggregation conditions with different compound concentrations, the samples were prepared as described previously. To account for the different concentrations of DMSO added to the compound stock solution, all reaction solutions were supplemented with DMSO to a final concentration of 2%.

The aggregation reaction curves were fitted using a Boltzmann sigmoidal equation (Origin 2018 software). Reaction half-time (*t*_50_), lag time, apparent rate constant, and end-point fluorescence intensities were determined from the fitted data as shown previously.^33^ Statistical analysis of the reaction parameters was done by using a one-way ANOVA Bonferroni means comparison (Origin 2018 software).

### Compound Fluorescence Assay

Each compound stock solution was diluted to 100 μM using 1× PBS. The resulting solutions were then placed in 96-well nonbinding half-area plates (Fisher Scientific, cat. no 3881, 100 μL volume, 3 repeats for each condition) and incubated at room temperature for 10 min. The sample fluorescence intensities were scanned by using 440 nm excitation and 480 nm emission wavelengths with a ClarioStar Plus plate reader. As a control, the ThT stock solution was diluted with 1× PBS and supplemented with 1% DMSO to a final dye concentration of 100 μM.

### Fourier-Transform Infrared Spectroscopy (FTIR)

After the aggregation assay, the 96-well plates were cooled to 22 °C and fibrils in each well were resuspended by pipetting, after which aliquots of 80 μL were recovered for the FTIR assay. To exchange H_2_O for D_2_O, the fibril solutions were centrifuged at 9000*g* for 15 min, and the pelleted aggregates were resuspended into 200 μL of D_2_O, supplemented with 400 mM NaCl (to improve sedimentation^38^). The centrifugation and resuspension procedure was repeated an additional two times. After the final centrifugation step, the fibrils were resuspended in 30 μL of D_2_O, supplemented with 400 mM NaCl. The sample FTIR spectra were then scanned as described previously,^39^ using a Bruker Invenio S FTIR spectrometer. D_2_O and water vapor spectra were subtracted from the sample spectra, which were then baseline corrected and normalized to the same area between 1700 and 1595 cm^–1^. Data processing was performed using GRAMS software.

To determine the different types of aggregate structures present in the samples, second-order derivatives were calculated for each spectrum, which were then compared and grouped based on similarities in their minimum positions. The grouping procedure was semisubjective due to possible α-syn fibril polymorphism, with some samples containing mixtures of different secondary structure aggregates.

### Transmission Electron Microscopy (TEM)

The α-syn fibril samples were diluted five times using 1× PBS (final concentration 20 μM). Before sample application, 300 mesh Formvar/carbon coated copper grids (Agar Scientific, U.K.) were UV irradiated. 5 μL of diluted α-syn aggregate solutions was then placed on the grids for 1 min, followed by drying with filter paper. The grids were negatively stained with 5 μL of 2% (w/v) uranyl acetate for 1 min, after which the excess solution was dried with filter paper. Finally, the grids were washed with 5 μL of Milli-Q water, and this washing/drying procedure was repeated 3 times. All TEM images were recorded using a Talos 120C (Thermo Fisher) transmission electron microscope operating at 120 V and equipped with 4k × 4k Ceta CMOS Camera. After acquisition, images were analyzed by using ImageJ software.

### Residual Monomer Quantification

α-Syn samples from the 200 μM compound concentration conditions were pooled together (6 × 100 μL) and centrifuged at 14 000*g* for 15 min. Part of the supernatant (100 μL) was carefully removed for analysis by SDS-PAGE electrophoresis. For control samples, the same procedure was carried out with the control fibril and 50 μM nonaggregated α-syn samples. 30 μL of aliquots of each supernatant was combined with a 4 times concentrated SDS-PAGE sample buffer solution (10 μL) and incubated at 98 °C for 10 min. The samples and a Spectra Multicolor Low Range Protein Ladder (ThermoFisher Scientific, cat. no. 26628) were then loaded on a 16% acrylamide gel (5 μL of each). The gel was stained with GelCode Blue Safe Protein Stain. The concentration of residual monomers was calculated with GelAnalyzer 23.1.1 software using the 50 μM nonaggregated α-syn sample band as a reference.

### Cell Viability Assay

The MTT assay was performed as previously described.^40^ In brief, SH-SY5Y cells were seeded in a 96-well plate (∼15 000 cells/well) and were incubated overnight. After incubation, the medium was changed to one containing either 20 μM of each selected compound or 20 μM of α-syn fibrils (from the control aggregation samples) with 20 μM of each compound. The medium also contained 0.2% DMSO from the compound stock solutions. For the control, the medium was supplemented with an equal concentration of PBS solution with DMSO. After 48 h of incubation, 10 μM of 3-(4,5-dimethylthiazol- 2-yl)-2,5-diphenyltetrazolium bromide (MTT) reagent (12.1 mM in PBS) was added to each well, followed by 2 h of incubation. To dissolve formazan crystals, 100 μL of 10% SDS with a 0.01 N HCl solution was added to each well. After 2 h, the absorbance was measured at 570 and 690 nm (as reference wavelength) using a ClarioStar Plus plate reader. Three separately prepared sets of samples with three repeats for each condition were used. Statistical analysis of the results was conducted by using Origin 2018 software one-way analysis of variance (ANOVA) Bonferroni means comparison (*n* = 9).

## Supporting Information Available

Additional experimental details, synthesis methods, materials, and methods, including ^1^H and ^13^C NMR spectra for all new compounds (PDF)
