# Supplementary material for: Imidazo[2,1-b][1,3]thiazine Derivatives as Potential Modulators of Alpha-Synuclein Amyloid Aggregation
Source: ACS Chem Neurosci. 2024 Nov 27;15(24):4418–30. doi: 10.1021/acschemneuro.4c00451 (PMC11660147; doi:10.1021/acschemneuro.4c00451)
Supplement: Supplementary file 1 — cn4c00451_si_001.pdf [file cn4c00451_si_001.pdf]

Supporting information

Imidazo[2,1-*b*][1,3]thiazine derivatives as potential modulators of alpha-synuclein amyloid aggregation

Indrė Misiūnaitė[#1], Kamilė Mikalauskaitė[#2], Martyna Paulauskaitė[1], Rūta Sniečkutė[2], Vytautas Smirnovas[2], Algirdas Brukštus[1], Mantas Žiaunys[2], Ieva Žutautė*[1]

[1]Institute of Chemistry, Faculty of Chemistry and Geosciences, Vilnius University, Naugarduko st. 24, LT-03225, Vilnius, Lithuania

[2]Institute of Biotechnology, Life Sciences Center, Vilnius University, Saulėtekio al. 7, LT-10257, Vilnius, Lithuania

E-mail: ieva.zutaute@chgf.vu.lt

Contents

General information 3

Figures 4

Synthesis of 2-(4-iodophenoxy)-N-isobutylacetamide **III** 6

Synthesis of *tert*-butyl (4-iodophenyl)(2-(isobutylamino)-2-oxoethyl)carbamate **VII** 6

General procedure for synthesis of 2-alkynyltioimidazoles **1** 8

General procedure for AuCl catalyzed cyclization reactions 10

Synthesis of 2-((4-(2*H*-benzo[4,5]imidazo[2,1-*b*][1,3]thiazin-4-yl)phenyl)amino)-N-isobutylacetamide **3** 12

Synthesis of 2-(4-(7H-imidazo[2,1-b][1,3]thiazin-5-yl)phenoxy)acetic acid **4** 12

Modification of 2-(4-(7*H*-imidazo[2,1-*b*][1,3]thiazin-5-yl)phenoxy)acetic acid **4** 13

Synthesis of 2-(4-((1H-imidazol-1-yl)methyl)phenoxy)-N-isobutylacetamide **6** 14

Synthesis of 2-(4-(1H-imidazole-1-carbonyl)phenoxy)-N-isobutylacetamide **7**... 14

References 16

Copies of NMR spectra 17

General information

All reagents and solvents were dried commonly before use according to standard procedures. Commercially available reagents were used without further purification unless otherwise noted. Oxygen- and moisture-sensitive reactions were carried out under an argon atmosphere. ^{1}H and ^{13}C NMR spectra were recorded in deuterated solvents on a Bruker Ascened^{TM} 400 MHz spectrometer. Chemical shifts (δ) are given in ppm with reference to solvent signals ($CDCl_3$: δ = 7.26 ppm for ^{1}H NMR, δ = 77.16 ppm for ^{13}C NMR, DMSO-d_6: δ = 2.50 ppm for ^{1}H NMR, δ = 39.5 ppm for ^{13}C NMR). The following standard abbreviations are used to indicate multiplicity: s – singlet, d – doublet, t – triplet, q – quartet, n – nonet, m – multiplet, and br.s – broad singlet. High-resolution mass spectra (HRMS) were recorded on an Agilent LC/MSD TOF mass spectrometer by electrospray ionization time-of-flight (ESI-TOF) reflection experiments. Infrared spectra were recorded on a PERKIN-ELMER 1000 FT-IR spectrometer with UATR annex. Microwave (MW) irradiation reactions were carried out using a CEM Discover® SP microwave synthesizer. Reactions were monitored by thin layer chromatography (TLC) carried out on 0.25 mm Merck silica plates (60 F_{254}), using UV light as the visualizing agent and/or vanillin and heat as a developing agent. Column chromatography was performed with Kieselgel 60 (40-63µm) silica gel. Melting points were measured on a "Stuart SMP10" and were uncorrected.

Figures

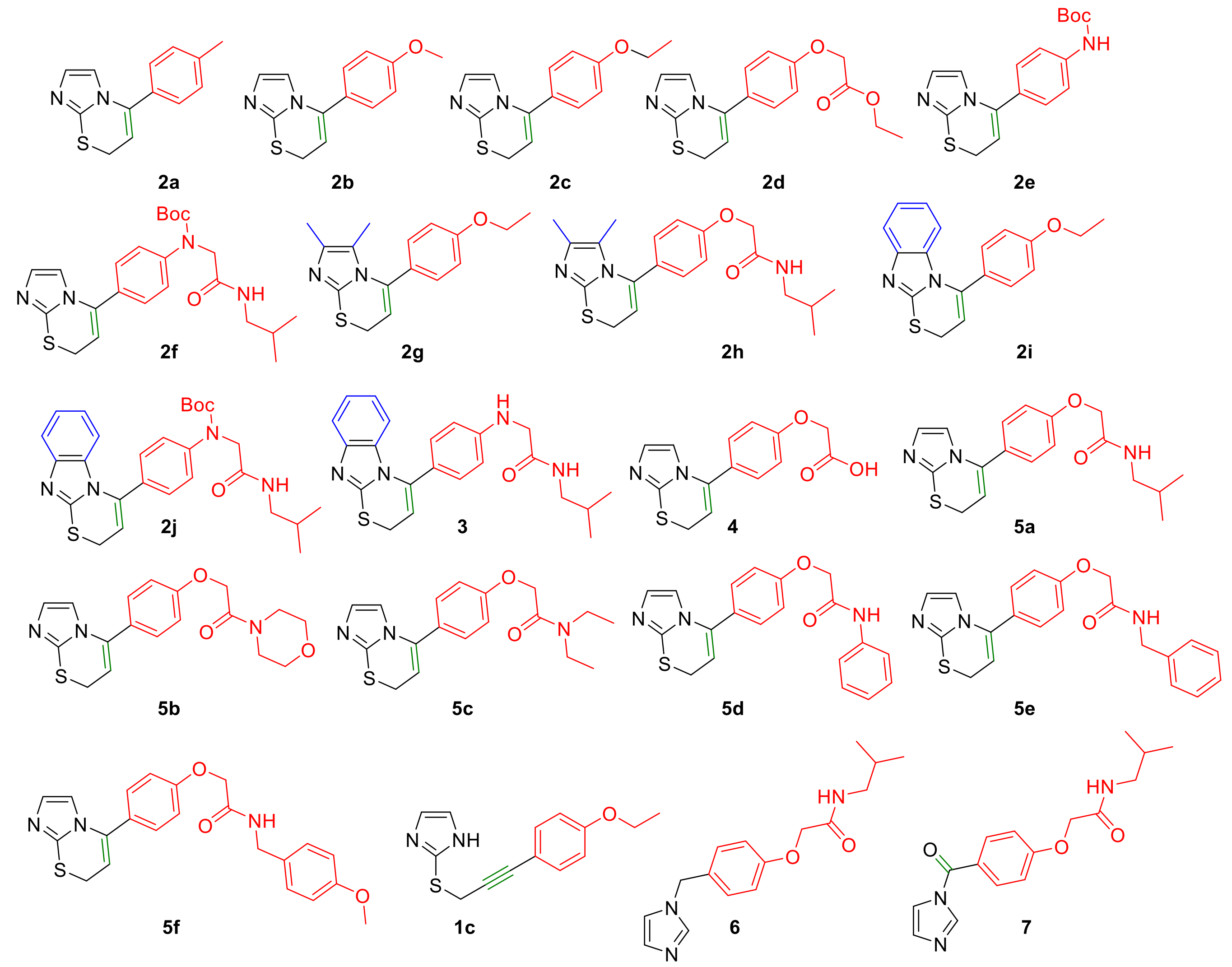

Figure S1. Structures of tested compounds.

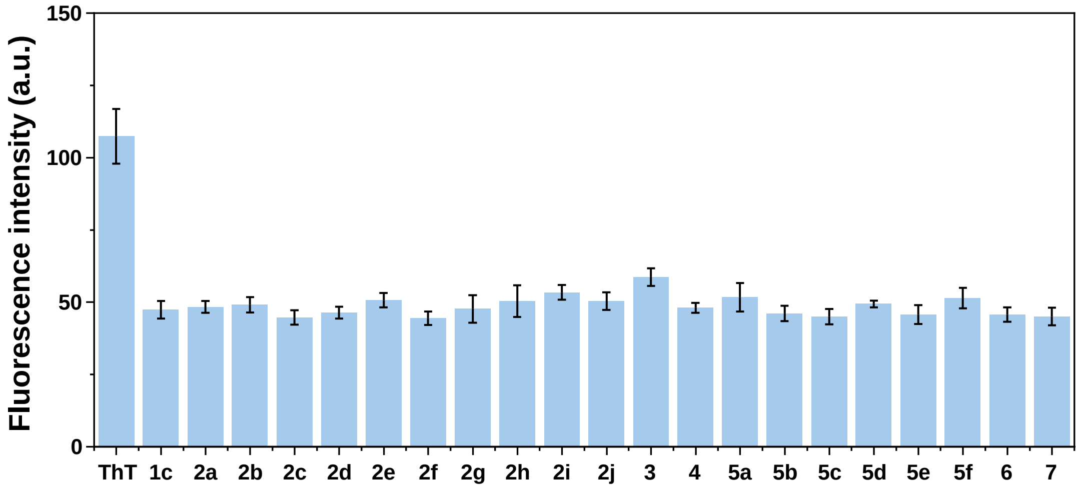

Figure S2. ThT and compound fluorescence intensities were measured using 440 nm excitation and 480 nm emission wavelengths (n=3, error bars are for one standard deviation).

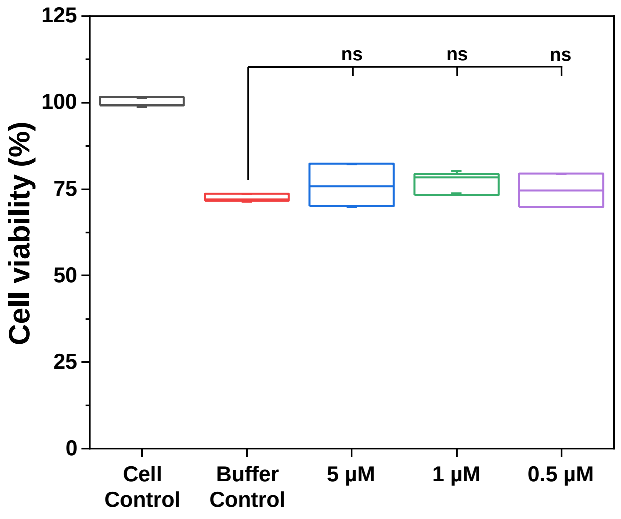

Figure S3. Effect of lower concentrations of compound **2j** on SH-SY5Y human neuroblastoma cells. For each condition, three technical repeats were performed. Box plots indicate the interquartile range; error bars are for one standard deviation. One-way ANOVA Bonferroni means comparison was conducted to determine differences between the buffer control and the samples (ns – not significant).

Synthesis of 2-(4-iodophenoxy)-N-isobutylacetamide **III**

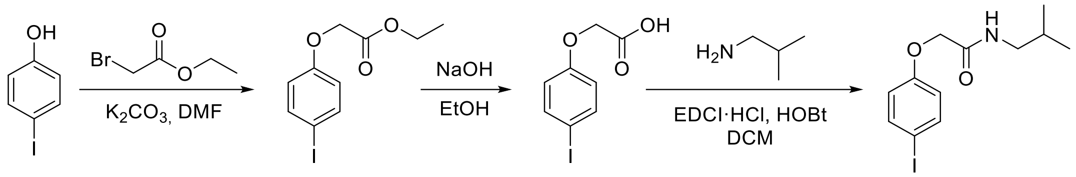

I **II** **III**

To the solution of 2-iodophenol (10 g, 45.40 mmol, 1.0 eq.) in DMF (50 ml) K_2CO_3 (9.42 g, 68.20 mmol, 1.5 eq.) was added. After 5 minutes, dropwise, ethyl 2-bromoacetate (6.04 ml, 54.6 mmol, 1.2 eq) was added. The reaction mixture was stirred at room temperature overnight. After reaction completion, monitored by TLC, DMF was evaporated at reduced pressure, residue was dissolved in ethyl acetate and washed with H_2O (6x350 ml) and brine. Then the organic phase was dried with anhydrous Na_2SO_4 and concentrated in reduced pressure. Without further purification, 13.64 g (98% yield) of yellowish crystals were obtained. The spectral data of compound **I** matched literature values[1]. ^{1}H NMR (400 MHz, $CDCl_3$): δ 7.52 (2H, d, *J* = 9.2 Hz), 6.65 (2H, d, *J* = 8.8 Hz), 4.55 (2H, s), 4.22 (2H, q, *J* = 7.2 Hz), 1.26 (3H, t, *J* = 7.2 Hz) ppm. ^{13}C NMR (100 MHz, $CDCl_3$): δ 168.43, 157.68, 138.28, 117.01, 84.03, 65.30, 61.42; 14.16 ppm.

To the solution of compound **I** (13.64 g, 44.60 mmol, 1.0 eq.) in EtOH (75 ml), NaOH (1.78 g, 44.60 mmol, 1.0 eq.) was added. The reaction mixture was stirred at room temperature overnight. After reaction completion, monitored by TLC, the EtOH was evaporated under reduced pressure, and the residue was dissolved in H_2O and acidified to pH2. Formed crystals were filtrated and washed with H_2O and Et_2O. Without further purification, 9.15 g (74% yield) of white crystals were obtained. The melting point and spectral data of compound **II** matched literature values[2]. M.p. 159-160°C. ^{1}H NMR (400 MHz, $CDCl_3$): δ 7.59 (2H, d, *J* = 8.0 Hz), 6.76 (2H, d, *J* = 8.4 Hz), 4.67 (2H, s) ppm. ^{13}C NMR (100 MHz, $CDCl_3$): δ 170.01, 157.74, 137.97, 117.30, 83.71, 64.47 ppm.

To the solution of compound **II** (2.5 g, 8.99 mmol, 1.0 eq.) in 80 ml of DCM, EDCI·HCl (2.59 g, 13.50 mmol, 1.5 eq.), HOBt (1.82 g, 13.40 mmol, 1.5 eq.) and 2-methylpropan-1-amine (1.07 ml, 10.80 mmol, 1.2 eq.) was added. The reaction mixture was stirred at room temperature overnight. After reaction completion, monitored by TLC, the mixture was diluted with H_2O and extracted with DCM. The combined organic layers were washed once with brine, dried with anhydrous Na_2SO_4, and concentrated at reduced pressure. The product **III** was isolated by column chromatography using eluent $CHCl_3$:CH_3CN (30:1), which afforded 2.43 g (81% yield) of white crystals, m.p. 71-72°C. IR υ_{max}, cm^{-1}: 3333 (-NH), 1662 (C=O). ^{1}H NMR (400 MHz, $CDCl_3$): δ 7.60 (2H, d, *J* = 8.8 Hz), 6.70 (2H, d, *J* = 8.8 Hz), 6.54 (1H, br.s., -NH), 4.46 (2H, s), 3.17 (2H, t, *J* = 6.6 Hz), 1.80 (1H, n, *J* = 6.8 Hz), 0.91 (6H, d, *J* = 6.8 Hz) ppm. ^{13}C NMR (100 MHz, $CDCl_3$): δ 168.27, 157.20, 138.71, 117.17, 84.61, 68.10, 46.44, 28.72, 20.16 ppm. HRMS (ESI-TOF): calc'd for $C_{12}H_{16}INO_2$ $[M+H]^+$: 334.0298, found 344.0302.

Synthesis of *tert*-butyl (4-iodophenyl)(2-(isobutylamino)-2-oxoethyl)carbamate **VII**

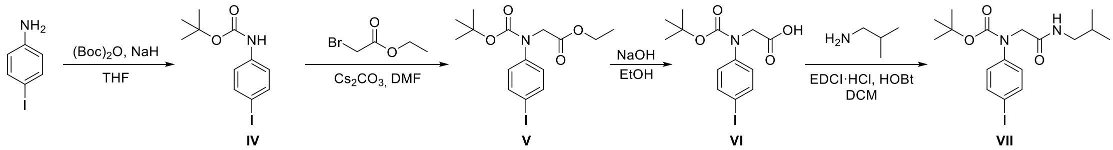

IV **V** **VI** **VII**

To the solution of NaH (1.05 g, 43.8 mmol, 1.2 eq.) in THF (170 ml) 4-iodoaniline (8.0 g, 36.5 mmol, 1 eq.) and Boc_2O (9.57 g, 43.8 mmol, 1.2 eq) was added. After reaction completion, monitored by TLC, the mixture was evaporated at reduced pressure, residue was dissolved in ethyl acetate and washed with H_2O

and brine. Then the organic phase was dried with anhydrous Na_2SO_4 and concentrated in reduced pressure. Then the crude product was dissolved in MeOH (60 ml), and Na_2CO_3 (4.64 g, 33.6 mmol, 2 eq.) was added. The reaction mixture was refluxed. After 3 hours the reaction mixture was evaporated at reduced pressure, residue was dissolved in ethyl acetate and washed with H_2O and brine. Then the organic phase was dried with anhydrous Na_2SO_4 and concentrated in reduced pressure. Without further purification, 5,14 g (44% yield) of white crystals were obtained, with m.p. 144-145 °C. IR υ_{max}, cm^{-1}: 3381 (-NH), 1701 (C=O). ^1H NMR (400 MHz, CDCl3): δ 7.57 (2H, d, *J* = 8.8 Hz), 7.14 (2H, d, *J* = 8.4 Hz), 6.48 (1H, s), 1.51 (9H, s). ^{13}C NMR (100 MHz, CDCl3): δ 152.57, 138.30, 137.94, 120.48, 85.85, 81.05, 28.43. The melting point and spectral data of compound **IV** matched literature values[3].

Cs_2CO_3 (6.67 g, 20.50 mmol, 1.3 eq.) was added to the solution of compound **IV** (5.03 g, 15.80 mmol, 1.0 eq.) in DMF. The reaction mixture was mixed for 6 days. After reaction completion, monitored by TLC, DMF was evaporated at reduced pressure, residue was dissolved in EA and washed with water and brine. Then the organic phase was dried with anhydrous Na_2SO_4 and concentrated in reduced pressure. The product **V** was isolated by column chromatography using eluent PE:Tol (20:1), which afforded 5.17 g (81% yield) of white crystals, m.p. 98-100 °C. IR υ_{max}, cm^{-1}: 1736 (C=O), 1695 (C=O). ^1H NMR (400 MHz, $CDCl_3$): δ 7.63 (2H, d, *J* = 8.6 Hz), 7.06 (2H, s), 4.26 – 4.16 (4H, m), 1.43 (9H, S), 1.28 (3H, t, *J* = 7.1 Hz) ppm. ^{13}C NMR (100 MHz, $CDCl_3$): δ 169.83, 154.17, 142.71, 137.97, 128.56, 90.96, 81.58, 61.41, 52.15, 28.29, 14.33 ppm. HRMS (ESI-TOF): calc'd for $C_{15}H_{20}INO_4$ $[M+H]^+$: 406.0510, $[M+H\text{-}Boc]^+$: 305.9985, found 305.9988.

To the solution of compound **V** (4.32 g, 10.67 mmol, 1.0 eq.) in EtOH (50 ml) and water (10 ml), NaOH (0.64 g, 16.0 mmol, 1.5 eq.) was added. The reaction mixture was stirred at room temperature overnight. After reaction completion, monitored by TLC, EtOH was evaporated at reduced pressure, residue was dissolved in H_2O and acidified to pH2. The formed crystals were filtrated and washed with H_2O. Yielding 67% (2,71 g) of white crystals (compound **VI**) with a m.p. 148-149°C. IR υ_{max}, cm^{-1}: 2929 (-OH), 1731 (C=O), 1690 (C=O). ^1H NMR (400 MHz, $CDCl_3$) δ 10.85 (1H, s, -OH), 7.64 (2H, d, *J* = 8.4 Hz), 7.04 (2H, d, *J* = 8.0 Hz), 4.31 (2H, s), 1.43 (9H, s) ppm. ^{13}C NMR (100 MHz, $CDCl_3$): δ 175.61, 154.22, 142.37, 138.09, 128.50, 91.26, 82.05, 51.87, 28.29 ppm. HRMS (ESI-TOF): calc'd for $C_{13}H_{16}INO_4$ $[M+H]^+$: 378.0197, $[M+H\text{-}Boc]^+$: 277.9672, found 277.9676.

To the solution of compound **VI** (2.5 g, 6.60 mmol, 1.0 eq.) in 30 ml of DCM, EDCI·HCl (1.91 g, 9.90 mmol, 1.5 eq.), HOBt (1.34 g, 9.90 mmol, 1.5 eq.) and 2-methylpropan-1-amine (0.66 ml, 6.60 mmol, 1.0 eq.) was added. The reaction mixture was stirred at room temperature overnight. After reaction completion, monitored by TLC, the mixture was diluted with H_2O and extracted with DCM. The combined organic layers were washed once with brine, dried with anhydrous Na_2SO_4, and concentrated at reduced pressure. Yielding, 90% (2.69 g) of white crystals (compound **VII**), m.p. 132-133°C. IR υ_{max}, cm^{-1}: 3297 (-NH), 1705 (C=O), 1651 (C=O). ^1H NMR (400 MHz, CDCl3): δ 7.63 (2H, d, *J* = 8.8 Hz), 7.04 (2H, d, *J* = 8.4 Hz), 6.25 (1H, s, -NH), 4.18 (2H, s), 3.12 (2H, t, *J* = 6.4 Hz), 1.76 (1H, n, J = 6.7 Hz), 1.44 (9H, s), 0.89 (6H, d, *J* = 6.8 Hz) ppm. ^{13}C NMR (100 MHz, CDCl3): δ 169.24, 154.52, 142.49, 138.04, 127.72, 90.83, 82.19, 54.37, 46.89, 28.62, 28.29, 20.13 ppm. HRMS (ESI-TOF): calc'd for $C_{17}H_{25}IN_2O_3$ $[M+H]^+$: 433.3099, $[M+H\text{-}Boc]^+$: 333.0458, found 333.0503.

General procedure for synthesis of 2-alkynyltioimidazoles **1**

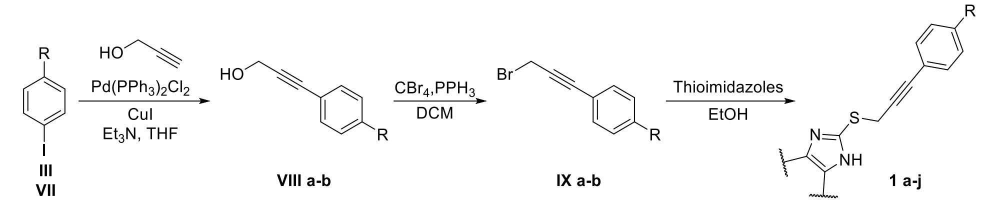

Sonogashira coupling: Solution of appropriate phenyl iodide (2.4 g (7.20 mmol) or 2.5 g (5.80 mmol), 1.0 eq) in Et_3N/THF (15ml/5ml) was stirred under argon atmosphere at room temperature. After 15 minutes, Pd(PPh3)2Cl2 (3mol%), prop-2-yn-1-ol (1.2 eq.), and CuI (1.5 mol%) were added. The reaction mixture was stirred at room temperature overnight. After reaction completion, monitored by TLC, Et_3N/THF was evaporated at reduced pressure, residue was dissolved in ethyl acetate and washed with saturated NH_4Cl aqueous solution and brine. Then the organic phase was dried with anhydrous Na_2SO_4 and concentrated in reduced pressure. The product was isolated by column chromatography.

2-(4-(3-hydroxyprop-1-yn-1-yl)phenoxy)-N-isobutylacetamide ***VIIIa***

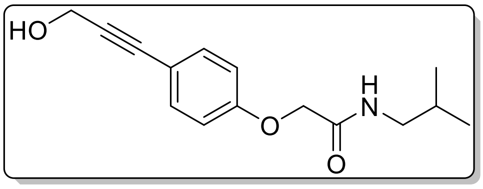

Yellowish crystals, m.p. 91-92°C, 1.64 g, 87% yield. Eluent – $CHCl_3$:CH_3CN (15:1→4:1). IR υ_{max}, cm^{-1}: 3361 (-NH, -OH), 2230 (C≡C), 1646 (C=O). ^{1}H NMR (400 MHz, $CDCl_3$): δ 7.35 (2H, d, *J* = 8.0 Hz), 6.82 (2H, d, *J* = 8.0 Hz), 6.61 (1H, br.s., -NH), 4.50 (2H, s), 4.47 (2H, s), 3.17 (2H, t, *J* = 6.2 Hz), 2.24 (1H, br.s., -OH), 1.81 (1H, n, *J* = 6.7 Hz), 0.90 (6H, d, *J* = 6.4 Hz) ppm. ^{13}C NMR (100 MHz, $CDCl_3$): δ 168.23; 157.17, 133.49, 116.43, 114.78, 86.99, 84.86, 67.46, 51.58; 46.43, 28.59; 20.11 ppm. HRMS (ESI-TOF): calc'd for $C_{15}H_{19}NO_3$ $[M+H]^+$: 262.1438, found 262.1440.

Tert-butyl (4-(3-hydroxyprop-1-yn-1-yl)phenyl)(2-(isobutylamino)-2-oxoethyl)carbamate ***VIIIb***

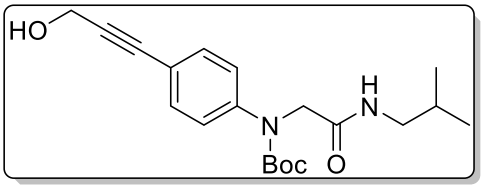

Light brown crystals, m.p. 52-53°C, 1.8 g, 84% yield. Eluent – $CHCl_3$:CH_3CN (4:1). IR υ_{max}, cm^{-1}: 3313 (-NH, -OH), 2249 (C≡C), 1701 (C=O), 1663 (C=O). ^{1}H NMR (400 MHz, CDCl3): δ 7.36 (2H, d, *J* = 8.4 Hz), 7.22 (2H, d, *J* = 8.4 Hz), 6.30 (1H, t, *J* = 6.0 Hz, -NH), 4.46 (2H, s), 4.21 (2H, s), 3.12 (2H, t, *J* = 6.4 Hz), 2.34 (1H, br.s., -OH), 1.76 (1H, n, *J* = 6.7 Hz), 1.44 (9H, s), 0.89 (6H, d, *J* = 6.4 Hz). ^{13}C NMR (100 MHz, CDCl3): δ 169.35, 154.54, 142.64, 132.33, 125.42, 120.42, 87.88, 84.94, 82.21, 54.32, 51.61, 46.90, 28.62, 28.28, 20.13. HRMS (ESI-TOF): calc'd for $C_{20}H_{28}N_2O_4$ $[M+H]^+$: 361.2122, $[M+H-Boc]^+$: 261.1598, found 261.1628.

Nucleophilic bromination: Solution of appropriate propargyl alcohol (0.75-1.6 g, 2.87-4.4 mmol, 1.0 eq) in DCM (15-40ml) was stirred at 0°C temperature. After 10 minutes, CBr_4 (1.14-1.77 g, 3.44-5.30 mmol, 1.2 eq.) was added. After another 15 minutes, PPh_3 (0.98-1.51 g, 3.73-5.80 mmol, 1.3 eq.) was added at 0°C temperature. The reaction mixture was stirred at room temperature overnight. After reaction completion, monitored by TLC, DCM was evaporated at reduced pressure, and the crude material was purified by column chromatography.

2-(4-(3-bromoprop-1-yn-1-yl)phenoxy)-N-isobutylacetamide ***IXa***

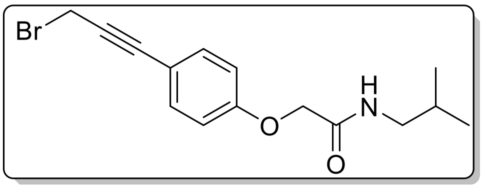

Yellowish crystals, m.p. 88-90°C, 911 mg, 98% yield. Eluent – $CHCl_3$:CH_3CN (30:1). IR $υ_{max}$, cm^{-1}: 3278 (-NH), 2218 (C≡C), 1658 (C=O). ^1H NMR (400 MHz, $CDCl_3$): δ 7.40 (2H, d, *J* = 7.2 Hz), 6.86 (2H, d, *J* = 7.6 Hz), 6.55 (1H, br.s., -NH), 4.49 (2H, s), 4.15 (2H, s), 3.16 (2H, t, *J* = 6.2 Hz), 1.84-1.78 (1H, m), 0.90 (6H, d, *J* = 6.4 Hz) ppm. ^{13}C NMR (100 MHz, $CDCl_3$): δ 167.72; 157.57, 133.79, 115.95, 114.79, 86.31, 83.72, 67.41, 46.40, 28.62; 20.13, 15.65 ppm. HRMS (ESI-TOF): calc'd for $C_{15}H_{18}BrNO_2$ $[M+H]^+$: 324.0594 and 326.0575, found 324.0592 and 326.0573.

Tert-butyl (4-(3-bromoprop-1-yn-1-yl)phenyl)(2-(isobutylamino)-2-oxoethyl)carbamate ***IXb***

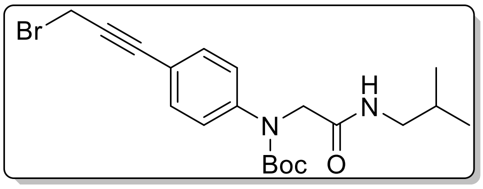

Dark brown crystals, m.p. 98-99°C, 1.6 g, 85% yield. Eluent – $CHCl_3$:CH_3CN (15:1). IR $υ_{max}$, cm^{-1}: 3290 (-NH), 2214 (C≡C), 1706 (C=O), 1665 (C=O). ^1H NMR (400 MHz, CDCl3): δ 7.39 (2H, d, *J* = 8.4 Hz), 7.24 (2H, d, *J* = 8.8 Hz), 6.25 (1H, t, *J* = 6.2 Hz, -NH), 4.21 (2H, s), 4.15 (2H, s), 3.12 (2H, t, *J* = 6.4 Hz), 1.76 (1H, n, *J* = 6.7 Hz), 1.45 (9H, s), 0.89 (6H, d, *J* = 6.8 Hz). ^{13}C NMR (100 MHz, CDCl3): δ 169.20, 154.47, 143.07, 132.55, 125.41, 119.83, 86.18, 84.64, 82.25, 54.33, 46.88, 28.64, 28.29, 20.14, 15.36. HRMS (ESI-TOF): calc'd for $C_{20}H_{27}BrN_2O_3$ $[M+H]^+$: 423.1278 and 425.1259, $[M+H-Boc]^+$: 323.0754 and 325.0735, found 323.0754 and 325.0735.

Nucleophilic substitution: To the solution of 2-thioimidazole/4,5-dimethyl-2-thioimidazole/2-thiobenzimidazole (from 50 mg to 1.45 g used, 1.0 eq.) in EtOH (5-40 ml) appropriate propargyl bromide (1.0 eq.) was added. The reaction mixture was stirred at room temperature overnight. After reaction completion, monitored by TLC, EtOH was evaporated at reduced pressure, residue was dissolved in chloroform and washed with saturated $NaHCO_3$ aqueous solution and brine. Then the organic phase was dried with anhydrous Na_2SO_4 and concentrated in reduced pressure. In necessity, the product was additionally purified.

The spectral data of compounds ***1a***, ***1c***, ***1d***, ***1g, 1i*** matched literature[4] values.

2-((3-(4-Methoxyphenyl)prop-2-yn-1-yl)thio)-1H-imidazole ***(1b)***

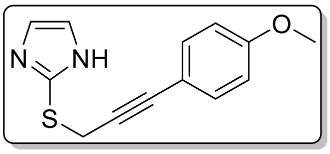

Yellowish crystals, m.p. 139-140°C, 222.8 mg, 83% yield. Eluent – $CHCl_3$:CH_3CN (4:1). IR $υ_{max}$, cm^{-1}: 2214 (C≡C). ^1H NMR (400 MHz, $CDCl_3$): δ 7.27 (2H, d, *J* = 8.8 Hz), 7.16 (2H, s), 6.80 (2H, d, *J* = 8.4 Hz), 3.89 (2H, s), 3.79 (3H, s) ppm. ^{13}C NMR (100 MHz, $CDCl_3$): δ 159.85, 138.62, 133.27, 124.56, 114.73, 114.07, 114.00, 84.46, 83.62, 55.43, 25.00 ppm. HRMS (ESI-TOF): calc'd for $C_{13}H_{12}N_2OS$ $[M+H]^+$: 245.0743, found 245.0744.

Tert-butyl (4-(3-((1H-imidazol-2-yl)thio)prop-1-yn-1-yl)phenyl)carbamate ***(1e)***

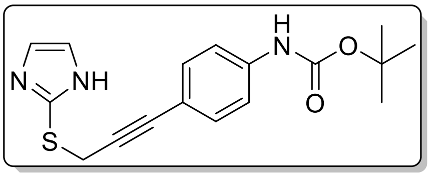

Yellowish crystals, m.p. 156°C, 316.5 mg, 58% yield. Eluent – $CHCl_3$:CH_3CN (15:1). IR $υ_{max}$, cm^{-1}: 3238 (-NH), 2222 (C≡C), 1695 (C=O). ^1H NMR (400 MHz, DMSO-d_6): δ 9.51 (1H, s, -NH), 7.40 (2H, d, *J* = 8.4 Hz), 7.18 (2H, d, *J* = 8.8 Hz), 7.12 (2H, s), 4.01 (2H, s), 1.41 (9H, s) ppm. ^{13}C NMR (100 MHz, DMSO-d_6): δ 152.54, 139.86, 137.19, 131.94,

124.16, 117.79, 115.27, 84.42, 83.39, 79.40, 28.05, 23.34 ppm. HRMS (ESI-TOF): calc'd for $C_{17}H_{19}N_3O_2S$ $[M+H]^+$: 330.1271, found 330.1265.

Tert-butyl (4-(3-((1H-imidazol-2-yl)thio)prop-1-yn-1-yl)phenyl)(2-(isobutylamino)-2-oxoethyl)carbamate ***(1f)***

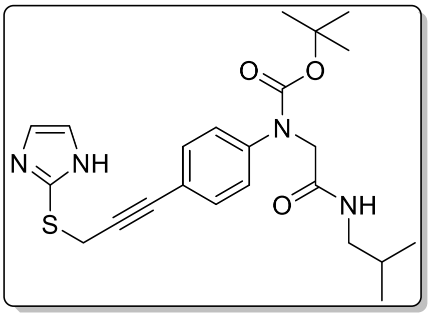

Light brown crystals, m.p. 81-82°C, 400.0 mg, 95% yield. IR υ_{max}, cm^{-1}: 3315 (-NH), 2207 (C≡C), 1701 (C=O), 1662 (C=O). ^1H NMR (400 MHz, $CDCl_3$): δ 7.23 (2H, d, *J* = 8.8 Hz), 7.15 (2H, d, *J* = 8.8 Hz), 7.13 (2H, s), 6.43 (1H, t, *J* = 6.2 Hz, -NH), 4.19 (2H, s), 3.85 (2H, s), 3.11 (2H, t, *J* = 6.4 Hz), 1,77 (1H, n, *J* = 6.7 Hz), 1.43 (9H, s), 0.89 (6H, d, *J* = 6.8 Hz) ppm. ^{13}C NMR (100 MHz, $CDCl_3$) δ 169.47, 154.65, 142.49, 138.04, 132.31, 125.53, 124.37, 120.57, 85.48, 83.77, 82.18, 54.36, 46.95, 28.64, 28.29, 24.96, 20.16 ppm. HRMS (ESI-TOF): calc'd for $C_{23}H_{30}N_4O_3S$ $[M+H]^+$: 443.2111, found 443.2120.

2-(4-(3-((4,5-Dimethyl-1H-imidazol-2-yl)thio)prop-1-yn-1-yl)phenoxy)-N-isobutylacetamide ***(1h)***

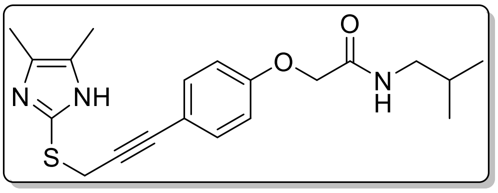

Yellowish crystals, m.p. 136-137°C, 208.7 mg, 91% yield. IR υ_{max}, cm^{-1}: 3283 (-NH), 2219 (C≡C), 1658 (C=O). ^1H NMR (400 MHz, $CDCl_3$): δ 7.29 (2H, d, *J* = 8.0 Hz), 6.82 (2H, d, *J* = 7.6 Hz), 6.61 (1H, t, *J* = 6.4 Hz, -NH), 4.48 (2H, s), 3.82 (2H, s), 3.17 (2H, t, *J* = 6.2 Hz), 2.16 (6H, s), 1.80 (1H, n, *J* = 6.6 Hz), 0.90 (6H, d, *J* = 6.4 Hz) ppm. ^{13}C NMR (100 MHz, $CDCl_3$): δ 167.90; 157.19, 134.35, 133.46, 129.39; 116.56, 114.75, 84.61, 83.84, 67.41, 46.44; 28.62; 25.44, 20.15; 11.01 ppm. HRMS (ESI-TOF): calc'd for $C_{20}H_{25}N_3O_2S$ $[M+H]^+$: 372.1740, found 372.1743.

Tert-butyl (4-(3-((1H-benzo[d]imidazol-2-yl)thio)prop-1-yn-1-yl)phenyl)(2-(isobutylamino)-2-oxoethyl)carbamate ***(1j)***

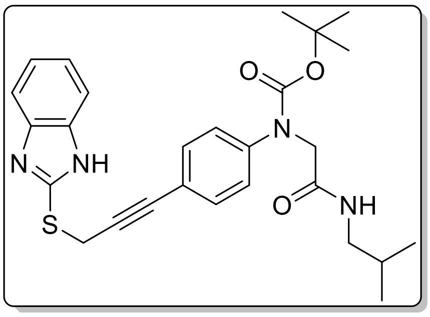

Yellowish crystals, m.p. 105-106°C, 447.8 mg, 96% yield. IR υ_{max}, cm^{-1}: 3307 (-NH), 2225 (C≡C), 1703 (C=O), 1662 (C=O). ^1H NMR (400 MHz, $CDCl_3$): δ 11.04 (1H, br.s., -NH), 7.74 – 7.36 (2H, m), 7.25 (2H, d, *J* = 9.6 Hz), 7.21 – 7.18 (2H, m), 7.16 (2H, d, *J* = 8.4 Hz), 6.39 (1H, t, *J* = 6.2 Hz, -NH), 4.25 (2H, s), 4.21 (2H, s), 3.13 (2H, t, *J* = 6.4 Hz), 1.77 (1H, n, *J* = 6.8 Hz), 1.43 (9H, s), 0.89 (6H, d, *J* = 6.4 Hz) ppm. ^{13}C NMR (100 MHz, $CDCl_3$) δ 169.48, 154.50, 148.73, 142.57, 139.04, 132.24, 125.40, 122.55, 120.16, 114.37, 84.39, 83.56, 81.99, 53.96, 46.96, 28.51, 28.17, 22.57, 20.07 ppm. HRMS (ESI-TOF): calc'd for $C_{27}H_{33}N_4O_3S$ $[M+H]^+$: 493.2268, found 493.2272.

General procedure for AuCl catalyzed cyclization reactions

The solution of compound ***1*** (from 50 mg to 200 mg, 0.17-0.45 mmol, 1.0 eq.) in 5 ml of DCE was stirred under an argon atmosphere at room temperature in a microwave vial for 15 min, then AuCl (10 mol%) was added, and the sealed vial was subjected to microwave irradiation. The reaction was carried out using a dynamic method at constant temperature (four cycles: three times for 40 min and one time for 20 min). The cycles were repeated if the reaction was not completed during this time. After reaction completion, the solvent was evaporated under reduced pressure, and the product was isolated by column chromatography.

The spectral data of compounds ***2a***, ***2c***, ***2d, 2g, 2i*** matched literature[4] values.

5-(4-Methoxyphenyl)-7H-imidazo[2,1-b][1,3]thiazine ***(2b)***

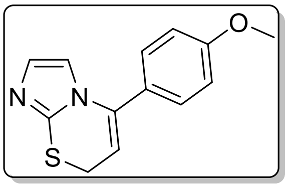

Brownish oil, 40.9 mg, 82% yield. One set of cycles (3x40 min + 20 min) at 50°C, eluent – $CHCl_3$:CH_3CN (4:1). ^1H NMR (400 MHz, $CDCl_3$): δ 7.29 (2H, d, *J* = 8.8 Hz), 6.99 (1H, br.s.), 6.92 (2H, d, *J* = 8.8 Hz), 6.84 (1H, br.s.), 5.57 (1H, t, *J* = 5.6 Hz), 3.84 (3H, s), 3.54 (2H, d, *J* = 5.6 Hz) ppm. ^{13}C NMR (100 MHz, $CDCl_3$): δ 160.66, 140.43, 139.08, 129.70, 127.60, 126.92, 118.44, 114.15, 105.93, 55.50, 24.45 ppm. HRMS (ESI-TOF): calc'd for $C_{13}H_{12}N_2OS$ $[M+H]^+$: 245.0743, found 245.0743.

Tert-butyl (4-(7H-imidazo[2,1-b][1,3]thiazin-5-yl)phenyl)carbamate ***(2e)***

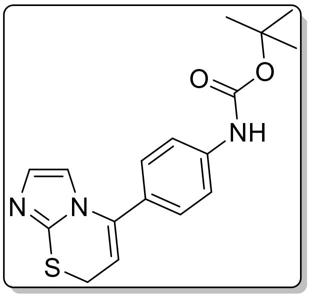

Yellowish crystals, m.p. deg. 138°C, 91.5 mg, 92% yield. One set of cycles (3x4 h + 20 min) at 50°C, eluent – $CHCl_3$:CH_3CN (4:1), IR $υ_{max}$, cm^{-1}: 3263 (-NH), 1711 (C=O). ^1H NMR (400 MHz, $CDCl_3$): δ 7.43 (2H, d, *J* = 8.4 Hz), 7.30 (2H, d, *J* = 8.4 Hz), 7.02 (1H, s), 6.85 (1H, s), 6.68 (1H, br.s., -NH), 5.61 (1H, t, *J* = 5.6 Hz), 3.56 (2H, d, *J* = 5.6 Hz), 1.53 (9H, s) ppm. ^{13}C NMR (100 MHz, $CDCl_3$): δ 152.66, 140.47, 139.82, 139.05, 129.16, 129.03, 127.71, 118.45 (3C), 106.39, 81.21, 28.44, 24.49 ppm. HRMS (ESI-TOF): calc'd for $C_{17}H_{19}N_3O_2S$ $[M+H]^+$: 330.1271, found 330.1268.

Tert-butyl (4-(7H-imidazo[2,1-b][1,3]thiazin-5-yl)phenyl)(2-(isobutylamino)-2-oxoethyl)carbamate ***(2f)***

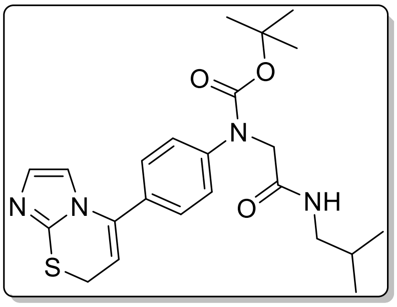

Yellowish crystals, m.p. deg. 157°C, 151.8 mg, 76% yield. One set of cycles (3x40 min + 20 min) at 50°C, eluent – $CHCl_3$:CH_3CN (7:1), IR $υ_{max}$, cm^{-1}: 3312 (-NH), 1703 (C=O), 1668 (C=O). ^1H NMR (400 MHz, $CDCl_3$): δ 7.36 (2H, d, *J* = 8.8 Hz), 7.33 (2H, d, *J* = 8.8 Hz), 7.00 (1H, s), 6.84 (1H, s), 6.31 (1H, t, *J* = 6.2 Hz, -NH), 5.63 (1H, t, *J* = 5.6 Hz), 4.25 (2H, s), 3.56 (2H, d, *J* = 5.6 Hz), 3.13 (2H, t, *J* = 6.4 Hz), 1.78 (1H, n, *J* = 6.7 Hz), 1.47 (9H, s), 0.90 (6H, d, *J* = 6.8 Hz) ppm. ^{13}C NMR (100 MHz, $CDCl_3$) δ 169.17, 154.50, 143.80, 138.72, 132.20, 128.81, 128.04, 125.75, 118.30, 107.26, 82.30, 54.31, 46.90, 28.65, 28.30, 24.45, 20.15. ppm. HRMS (ESI-TOF): calc'd for $C_{23}H_{30}N_4O_3S$ $[M+H]^+$: 443.2111, found 443.2112.

2-(4-(2,3-Dimethyl-7H-imidazo[2,1-b][1,3]thiazin-5-yl)phenoxy)-N-isobutylacetamide ***(2h)***

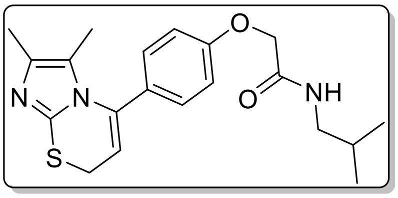

Yellowish crystals, m.p. 115-116°C, 39.3 mg, 52% yield. One set of cycles (3x40 min + 20 min) at 80°C, eluent – $CHCl_3$:CH_3CN (15:1→4:1). IR $υ_{max}$, cm^{-1}: 3314 (-NH), 1669 (C=O). ^1H NMR (400 MHz, $CDCl_3$): δ 7.23 (2H, d, *J* = 8.4 Hz), 7.01 (1H, s), 6.93 (2H, d, *J* = 8.4 Hz), 6.60 (1H, br.s., -NH), 5.61 (1H, t, *J* = 6.2 Hz), 4.52 (2H, s), 3.44 (2H, d, *J* = 6.0 Hz), 3.18 (2H, t, *J* = 6.4 Hz), 2.11 (3H, s), 1.81 (1H, n, *J* = 6.7 Hz), 1.54 (3H, s), 0.91 (6H, d, *J* = 6.8 Hz) ppm. ^{13}C NMR (100 MHz, $CDCl_3$): δ 167.79; 157.76; 140.07; 139.29; 134.41; 129.75; 129.28; 122.37; 114.82; 109.59; 67.47; 46.41; 28.64; 25.26; 20.15; 12.65; 11.98 ppm. HRMS (ESI-TOF): calc'd for $C_{20}H_{25}N_3O_2S$ $[M+H]^+$: 372.1740, found 372.1742.

*Tert-butyl (4-(2H-benzo[4,5]imidazo[2,1-b][1,3]thiazin-4-yl)phenyl)(2-(isobutylamino)-2-oxoethyl)carbamate **(2j)***

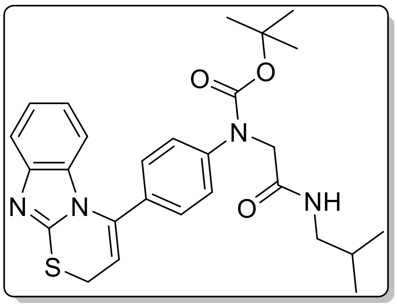

White crystals, m.p. 109-111°C, 175.0 mg, 88% yield. One set of cycles (3x40 min + 20 min) at 50°C, eluent – $CHCl_3$:CH_3CN (3:1), IR υ_{max}, cm^{-1}: 3318 (-NH), 1702 (C=O), 1669 (C=O). ^1H NMR (400 MHz, $CDCl_3$): δ 7.66 (1H, d, *J* = 8.0 Hz), 7.33 (2H, d, *J* = 8.0 Hz), 7.25 (2H, d, *J* = 8.8 Hz), 7.16 (1H, t, *J* = 7.6 Hz), 6.88 (1H, t, *J* = 7.6 Hz), 6.35 (1H, t, *J* = 6.2 Hz, -NH), 6.21 (1H, d, *J* = 8.0 Hz), 5.74 (1H, t, *J* = 6.2 Hz), 4.28 (2H, s), 3.62 (2H, d, *J* = 6.4 Hz), 3.15 (2H, t, *J* = 6.4 Hz), 1.79 (1H, n, *J* = 6.7 Hz), 1.47 (9H, s), 0.91 (6H, d, *J* = 6.8 Hz) ppm. ^{13}C NMR (100 MHz, $CDCl_3$): δ 169.25, 154.65, 150.50, 143.84, 142.89, 139.22, 132.39, 131.87, 128.81, 125.90, 123.01, 122.25, 119.10, 112.69, 108.72, 82.19, 54.42, 46.91, 28.66, 28.30, 24.80, 20.16 ppm. HRMS (ESI-TOF): calc'd for $C_{27}H_{32}N_4O_3S$ $[M+H]^+$: 493.2268, found 493.2273.

Synthesis of 2-((4-(2*H*-benzo[4,5]imidazo[2,1-*b*][1,3]thiazin-4-yl)phenyl)amino)-N-isobutylacetamide **3**

To the solution of **2j** (140 mg, 0,28 mmol, 1.0 eq.) in DCM (5 ml), TFA (1 ml) was added dropwise. The reaction mixture was stirred at room temperature overnight. After reaction completion, monitored by TLC, the mixture was diluted with $NaHCO_3$ aqueous solution and extracted with DCM. The combined organic layers were washed once with brine, dried with anhydrous Na_2SO_4, and concentrated at reduced pressure. The product was isolated by column chromatography using eluent $CHCl_3$:CH_3CN (4:1).

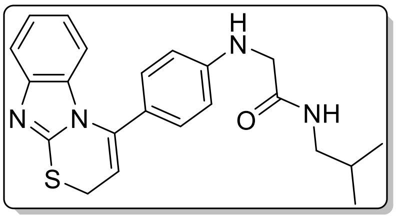

Yellowish crystals, m.p. 234°C, 68.5 mg, 61% yield. IR υ_{max}, cm^{-1}: 3260 (-NH), 1647 (C=O). ^1H NMR (400 MHz, DMSO-d_6): δ 7.89 (1H, t, *J* = 6.0 Hz, -NH), 7.55 (1H, d, *J* = 8.0 Hz), 7.13 (1H, t, *J* = 7.8 Hz), 7.00 (2H, d, *J* = 8.4 Hz), 6.91 (1H, t, *J* = 7.8 Hz), 6.59 (2H, d, *J* = 8.4 Hz), 6.34 (1H, br. s., -NH), 6.24 (1H, d, *J* = 8.4 Hz), 5.71 (1H, t, *J* = 6.2 Hz), 3.73 (2H, d, *J* = 6.0 Hz), 3.70 (2H, s), 2.93 (2H, t, *J* = 6.4 Hz), 1.69 (1H, n, *J* = 6.7 Hz), 0.80 (6H, d, *J* = 6.8 Hz) ppm. ^{13}C NMR (100 MHz, DMSO-d_6): δ 169.71, 150.07, 149.29, 142.42, 139.05, 132.29, 128.85, 122.34, 121.69, 121.51, 118.35, 112.58, 111.92, 106.98, 46.52, 45.95, 28.12, 24.10, 20.08 ppm. HRMS (ESI-TOF): calc'd for $C_{22}H_{24}N_4OS$ $[M+H]^+$: 393.1744, found 393.1745.

Synthesis of 2-(4-(7H-imidazo[2,1-b][1,3]thiazin-5-yl)phenoxy)acetic acid **4**

To a solution of **2d** (883.2 mg, 2.79 mmol, 1.0 eq.) in EtOH (20 ml), NaOH (111.7 mg, 2.79 mmol, 1.0 eq.) was added. The reaction mixture was stirred at room temperature overnight. After reaction completion, monitored by TLC, EtOH was evaporated at reduced pressure, and residue was dissolved in H_2O and acidified to pH2. Formed crystals were filtrated and washed with H_2O and Et_2O.

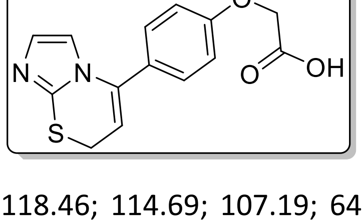

White crystals, m.p. 227-228°C, 579.5 mg, 72% yield. IR υ_{max}, cm^{-1}: 3135 (-OH), 1734 (C=O). ^1H NMR (400 MHz, DMSO-d_6): δ 13.08 (1H, br.s., -OH), 7.32 (2H, d, *J* = 8.4 Hz), 7.00 (2H, d, *J* = 8.4 Hz), 6.98 (1H, s), 6.96 (1H, s), 5.75 (1H, t, *J* = 5.6 Hz), 4.74 (2H, s), 3.65 (2H, d, *J* = 5.6 Hz) ppm. ^{13}C NMR (100 MHz, DMSO-d_6): δ 170.01; 158.55; 139.24; 137.71; 129.29; 127.55; 127.00; 118.46; 114.69; 107.19; 64.52; 23.63 ppm. HRMS (ESI-TOF): calc'd for $C_{14}H_{12}N_2O_3S$ $[M+H]^+$: 289.0641, found 289.0641.

Modification of 2-(4-(7*H*-imidazo[2,1-*b*][1,3]thiazin-5-yl)phenoxy)acetic acid **4**

To the solution of compound ***4*** (50 mg, 0.17 mmol, 1.0 eq.) in 5 ml of DCM, EDCI·HCl (49.9 mg, 0.26 mmol, 1.5 eq.), HOBt (35.2 mg, 0.26 mmol, 1.5 eq.) and appropriate amine (0.21 mmol, 1.2 eq.) was added. The reaction mixture was stirred at room temperature overnight. After reaction completion, monitored by TLC, the mixture was diluted with H_2O and extracted with DCM. The combined organic layers were washed once with brine, dried with anhydrous Na_2SO_4, and concentrated at reduced pressure. The product was isolated by column chromatography using eluent $CHCl_3$:CH_3CN (4:1).

*2-(4-(7H-imidazo[2,1-b][1,3]thiazin-5-yl)phenoxy)-N-isobutylacetamide **(5a)***

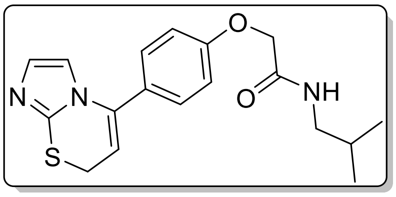

Brow crystals, m.p. 79-80°C, 28.0 mg, 47% yield. IR υ_{max}, cm^{-1}: 3270 (-NH), 1658 (C=O). ^1H NMR (400 MHz, $CDCl_3$): δ 7.33 (2H, d, *J* = 8.8 Hz), 7.01 (1H, s), 6.99 (1H, d, *J* = 1.6 Hz), 6.96 (2H, d, *J* = 8.8 Hz), 6.80 (1H, d, *J* = 1.6 Hz), 6.62 (br. s., 1H, -NH), 5.59 (1H, t, *J* = 5.8 Hz), 4.53 (2H, s), 3.55 (2H, d, *J* = 5.6 Hz), 3.18 (2H, t, *J* = 6.6 Hz), 1.81 (1H, n, *J* = 6.7 Hz), 0.90 (6H, d, *J* = 6.8 Hz) ppm. ^{13}C NMR (100 MHz, $CDCl_3$): δ 167.71; 158.19; 140.49; 138.69; 130.00; 128.48; 127.81; 118.24; 114.96; 106.56; 67.54; 46.42; 28.61; 24.42; 20.11 ppm. HRMS (ESI-TOF): calc'd for $C_{18}H_{21}N_3O_2S$ $[M+H]^+$: 344.1427, found 344.1426.

*2-(4-(7H-imidazo[2,1-b][1,3]thiazin-5-yl)phenoxy)-1-morpholinoethan-1-one **(5b)***

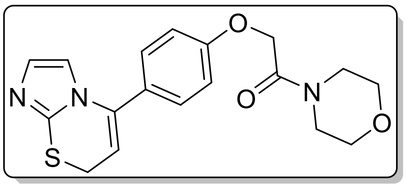

Yellow crystals, m.p. 163-164°C, 49.2 mg, 79% yield. IR υ_{max}, cm^{-1}: 1657 (C=O). ^1H NMR (400 MHz, $CDCl_3$): δ 7.29 (2H, d, *J* = 8.4 Hz), 6.99 (1H, s), 6.97 (2H, d, *J* = 8.8 Hz), 6.82 (1H, d, *J* = 1.6 Hz), 5.57 (1H, t, *J* = 5.8 Hz), 4.74 (2H, s), 3.69-3.58 (8H, m), 3.54 (2H, d, *J* = 5.6 Hz) ppm. ^{13}C NMR (100 MHz, $CDCl_3$): δ 166.23; 158.82; 140.45; 138.81; 129.87; 128.06; 127.67; 118.35; 114.85; 106.37; 67.54; 66.90; 66.78; 45.95; 42.53; 24.42 ppm. HRMS (ESI-TOF): calc'd for $C_{18}H_{19}N_3O_3S$ $[M+H]^+$: 358.1220, found 358.1220.

*2-(4-(7H-imidazo[2,1-b][1,3]thiazin-5-yl)phenoxy)-N,N-diethylacetamide **(5c)***

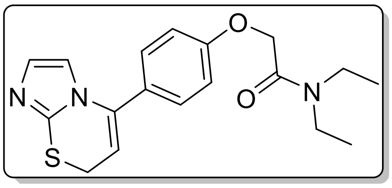

Brownish oil, 43.5 mg, 73% yield. IR υ_{max}, cm^{-1}: 1639 (C=O). ^1H NMR (400 MHz, $CDCl_3$): δ 7.27 (2H, d, *J* = 9.2 Hz), 6.98 (1H, d, *J* = 1.6 Hz), 6.96 (2H, d, *J* = 8.8 Hz), 6.83 (1H, d, *J* = 1.6 Hz), 5.56 (1H, t, *J* = 5.6 Hz), 4.71 (2H, s), 3.53 (2H, d, *J* = 6.0 Hz), 3.43-3.35 (4H, m), 1.22 (3H, t, *J* = 7.2 Hz), 1.13 (3H, t, *J* = 7.2 Hz) ppm. ^{13}C NMR (100 MHz, $CDCl_3$): δ 166.62; 159.23; 140.40; 138.92; 129.74; 127.68; 127.61; 118.42; 114.90; 106.19; 67.33; 41.61; 40.48; 24.42; 14.45; 12.91 ppm. HRMS (ESI-TOF): calc'd for $C_{18}H_{21}N_3O_2S$ $[M+H]^+$: 344.1427, found 344.1427.

*2-(4-(7H-imidazo[2,1-b][1,3]thiazin-5-yl)phenoxy)-N-phenylacetamide **(5d)***

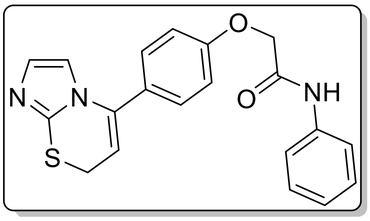

Brownish crystals, m.p. 95-96°C, 47.4 mg, 75% yield. IR υ_{max}, cm^{-1}: 3296 (-NH), 1667 (C=O). ^1H NMR (400 MHz, $CDCl_3$): δ 8.32 (1H, br.s.), 7.60 (2H, d, *J* = 7.6 Hz), 7.38-7.34 (2H, m), 7.36 (2H, d, *J* = 8.8 Hz), 7.16 (1H, t, *J* = 7.4 Hz), 7.04 (2H, d, *J* = 8.8 Hz), 7.01 (1H, s), 6.83 (1H, d, *J* = 1.6 Hz), 5.61 (1H, t, *J* = 5.8 Hz), 4.66 (2H, s), 3.56 (2H, d, *J* = 5.6 Hz) ppm. ^{13}C NMR (100 MHz, $CDCl_3$): δ 165.80; 158.00; 140.55; 138.66; 136.84; 130.10; 129.26; 128.83; 127.88; 125.14; 120.27; 118.29; 115.12; 106.70; 67.75; 24.44 ppm. HRMS (ESI-TOF): calc'd for $C_{20}H_{17}N_3O_2S$ $[M+H]^+$: 364.1114, found 364.1114.

2-(4-(7H-imidazo[2,1-b][1,3]thiazin-5-yl)phenoxy)-N-benzylacetamide ***(5e)***

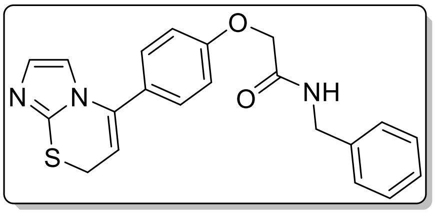

Brown crystals, m.p. 95-96°C, 48.1 mg, 73% yield. IR υ_{max}, cm^{-1}: 3311 (-NH), 1661 (C=O). ^1H NMR (400 MHz, $CDCl_3$): δ 7.35-7.27 (7H, m), 6.99 (2H, br.s.), 6.94 (2H, d, *J* = 8.4 Hz), 6.81 (1H, s), 5.57 (1H, t, *J* = 5.6 Hz), 4.58 (2H, s), 4.55 (2H, d, *J* = 6.0 Hz), 3.53 (2H, d, *J* = 5.6 Hz) ppm. ^{13}C NMR (100 MHz, $CDCl_3$): δ 167.67; 158.07; 138.78; 137.76; 129.84; 129.52; 128.84; 128.60; 128.15; 127.82; 127.77; 118.46; 114.82; 106.47; 67.50; 43.09; 24.38 ppm. HRMS (ESI-TOF): calc'd for $C_{21}H_{19}N_3O_2S$ $[M+H]^+$: 378.1271, found 378.1272.

2-(4-(7H-imidazo[2,1-b][1,3]thiazin-5-yl)phenoxy)-N-(4-methoxybenzyl)acetamide ***(5f)***

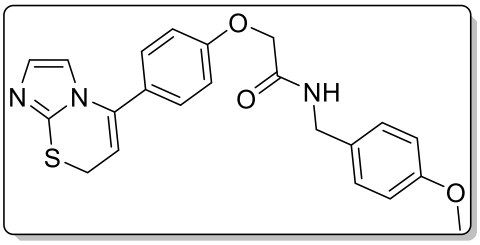

Orange crystals, m.p. 75-76°C, 70.7 mg, 98% yield. IR υ_{max}, cm^{-1}: 3135 (-NH), 1654 (C=O). ^1H NMR (400 MHz, $CDCl_3$): δ 7.30 (2H, d, *J* = 8.4 Hz), 7.21 (2H, d, *J* = 8.4 Hz), 7.00 (1H, s), 6.93 (2H, d, *J* = 8.4 Hz), 6.90-6.88 (1H, m, -NH), 6.85 (2H, d, *J* = 8.8 Hz), 6.80 (1H, s), 5.59 (1H, t, *J* = 5.6 Hz), 4.56 (2H, s), 4.47 (2H, d, *J* = 6.0 Hz), 3.78 (3H, s), 3.55 (2H, d, *J* = 5.6 Hz) ppm. ^{13}C NMR (100 MHz, $CDCl_3$): δ 167.54; 159.25; 158.15; 140.55; 138.63; 129.96; 129.82; 129.26; 128.33; 127.46; 118.30; 114.97; 114.22; 106.65; 67.47; 55.41; 42.63; 24.39 ppm. HRMS (ESI-TOF): calc'd for $C_{22}H_{21}N_3O_3S$ $[M+H]^+$: 408.1376, found 408.1361.

Synthesis of 2-(4-((1H-imidazol-1-yl)methyl)phenoxy)-N-isobutylacetamide **6**

To a solution of imidazole (22 mg, 0.33 mmol, 1.0 eq.) in DMF (2 ml), K_2CO_3 (55.2 mg, 0.40 mmol, 1.2 eq.) was added. After 5 minutes, 2-(4-(bromomethyl)phenoxy)-N-isobutyl acetamide (100 mg, 0.33 mmol, 1.0 eq) was added. The reaction mixture was stirred at room temperature overnight. After reaction completion, monitored by TLC, DMF was evaporated at reduced pressure. Residue was dissolved in MTBE and washed with H_2O (3x350 ml) and brine. Then the organic phase was dried with anhydrous Na_2SO_4 and concentrated in reduced pressure. The product was isolated by column chromatography using eluent $CHCl_3$:CH_3CN (4:1→1:1).

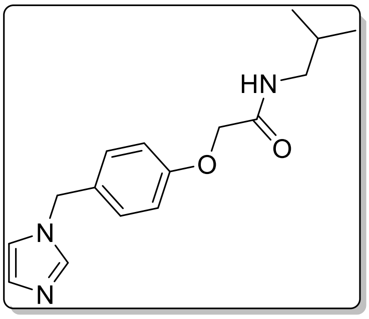

White crystals, 11.6 mg, 12% yield. ^1H NMR (400 MHz, $CDCl_3$): δ 7.54 (1H, br.s.), 7.14 (2H, d, *J* = 8.8 Hz), 7.09 (1H, br.s.), 6.90 (3H, m), 6.57 (1H, br.s., -NH), 5.06 (2H, s), 4.49 (2H, s), 3.17 (2H, t, *J* = 6.6 Hz), 4.52 (2H, s), 1.80 (1H, n, *J* = 6.7 Hz), 1.54 (3H, s), 0.90 (6H, d, *J* = 6.4 Hz) ppm. ^{13}C NMR (100 MHz, $CDCl_3$): δ 167.89; 157.27; 129.90; 129.22; 115.26; 67.57; 50.36; 46.40; 28.65; 20.14 ppm. HRMS (ESI-TOF): calc'd for $C_{16}H_{21}N_3O_2$ $[M+H]^+$: 288.1707, found 288.1707.

Synthesis of 2-(4-(1H-imidazole-1-carbonyl)phenoxy)-N-isobutylacetamide **7**

To a solution of imidazole (18.4 mg, 0.28 mmol, 1.0 eq.) in DCM (7 ml) at 0°C, Et_3N (0.05 ml, 0.36 mmol, 1.3 eq.) was added dropwise. After 5 minutes, a solution of 4-(2-(isobutylamino)-2-oxoethoxy)benzoyl chloride (75 mg, 0.28 mmol, 1.0 eq) in DCM (2.5 ml) was added dropwise. The reaction mixture was stirred overnight, allowing the temperature to rise from 0°C to room temperature. After reaction completion,

monitored by TLC, the reaction was quenched with 10% HCl solution and extracted with DCM. Then the organic phase was dried with anhydrous Na_2SO_4 and concentrated in reduced pressure. The product was isolated by column chromatography using eluent $CHCl_3$:CH_3CN (4:1→1:1).

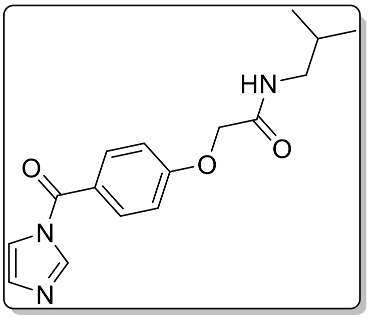

White crystals, 11.0 mg, 13% yield. Eluent – $CHCl_3$:CH_3CN (4:1→1:1). ^1H NMR (400 MHz, $CDCl_3$): δ 8.06 (1H, s), 7.83 (2H, d, *J* = 8.8 Hz), 7.52 (1H, s), 7.16 (1H, s), 7.08 (2H, d, *J* = 8.8 Hz), 6.56 (1H, br.s., -NH), 4.60 (2H, s), 3.19 (2H, t, *J* = 6.6 Hz), 1.82 (1H, n, *J* = 6.7 Hz), 0.92 (6H, d, *J* = 6.4 Hz) ppm. ^{13}C NMR (100 MHz, $CDCl_3$): δ 167.20; 165.28; 161.38; 138.20; 132.61; 131.00; 125.57; 118.28; 115.13; 67.55; 46.49; 28.63; 20.14 ppm. HRMS (ESI-TOF): calc'd for $C_{16}H_{19}N_3O_3$ $[M+H]^+$: 302.1499, found 302.1519.

References

1 H. S. Ban, H. Minegishi, K. Shimizu, M. Maruyama, Y. Yasui and H. Nakamura, *ChemMedChem*, 2010, **5**, 1236–1241.

2 X. He, Z. Yu, S. Jiang, P. Zhang, Z. Shang, Y. Lou and J. Wu, *Bioorganic & Medicinal Chemistry Letters*, 2015, **25**, 5601–5603.

3 A. Yokoyama, T. Maruyama, K. Tagami, H. Masu, K. Katagiri, I. Azumaya and T. Yokozawa, *Org. Lett.*, 2008, **10**, 3207–3210.

4 I. Misiūnaitė, R. Bajarūnaitė, R. Bukšnaitienė, A. Brukštus and I. Žutautė, *Synthesis*, 2023, **55**, 4213–4223.

Copies of NMR spectra

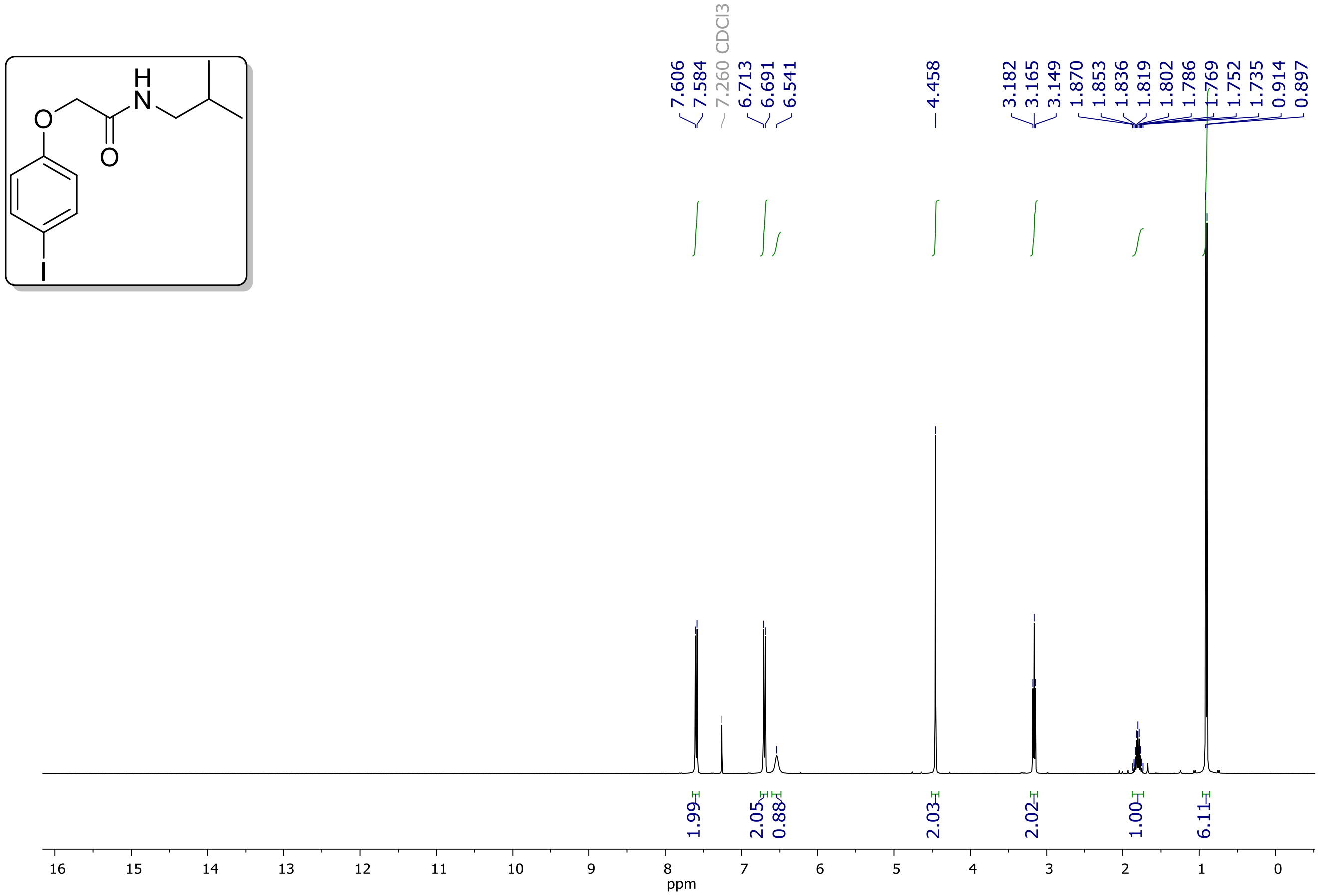

Figure S4. ^{1}H NMR (400 MHz, $CDCl_3$) spectrum of **III**.

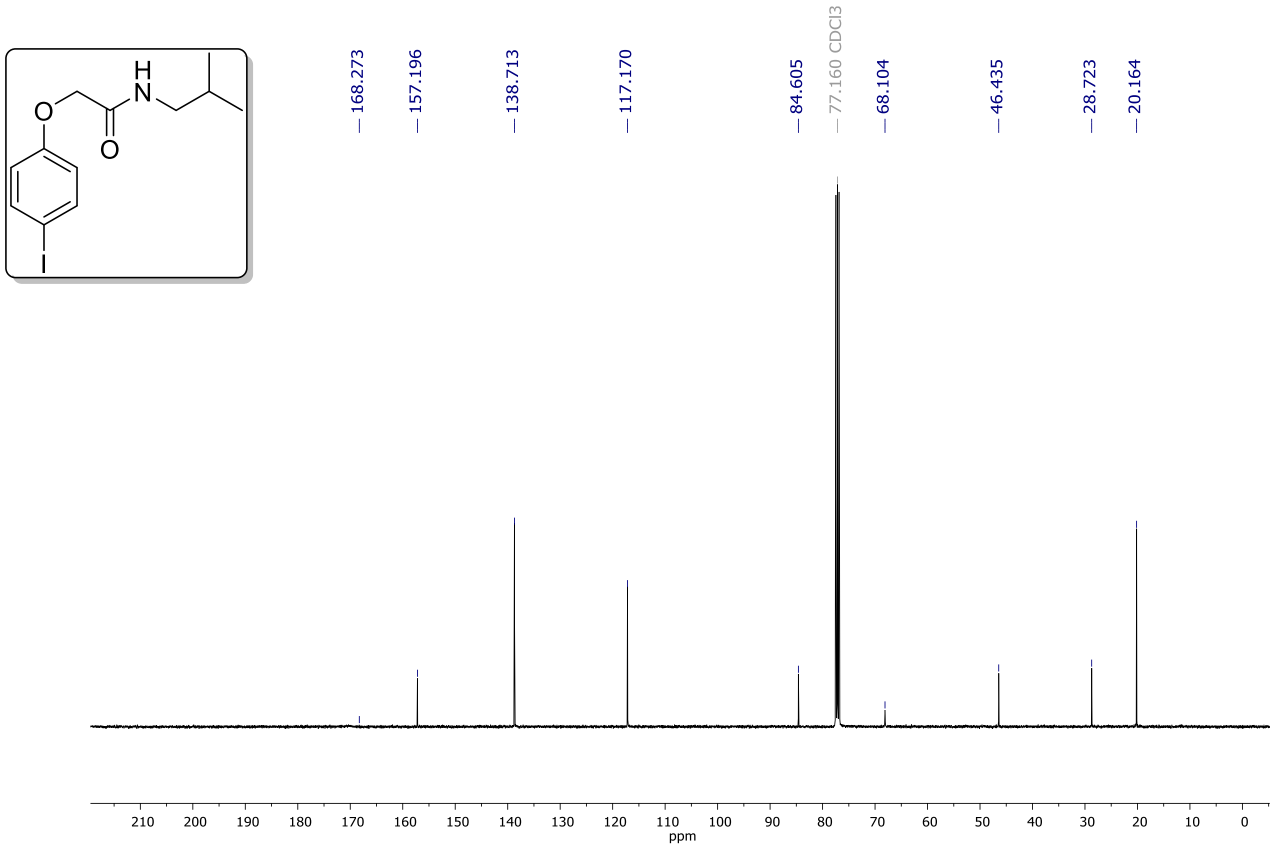

Figure S5. ^{13}C NMR (100 MHz, $CDCl_3$) spectrum of **III**.

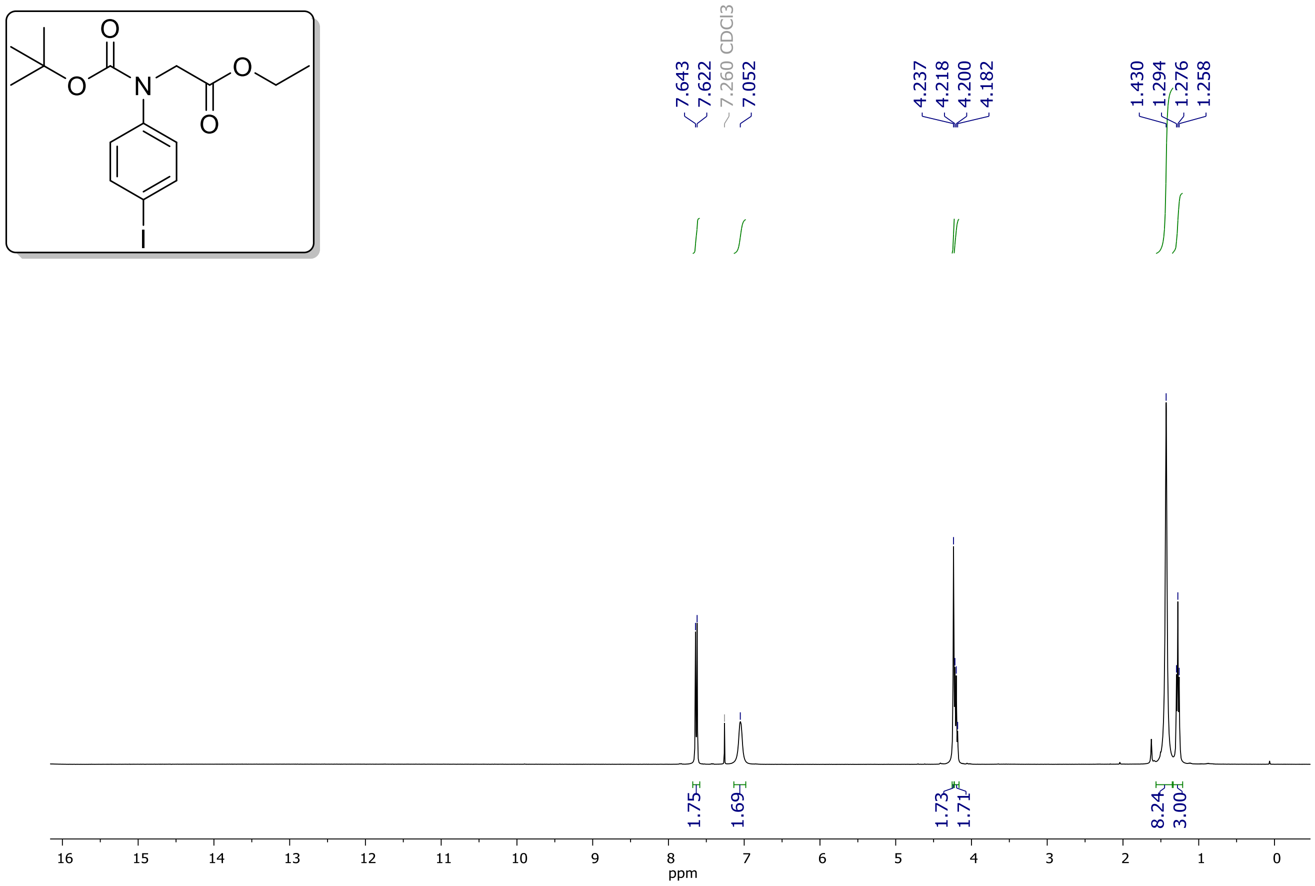

Figure S6. ^{1}H NMR (400 MHz, $CDCl_3$) spectrum of **V**.

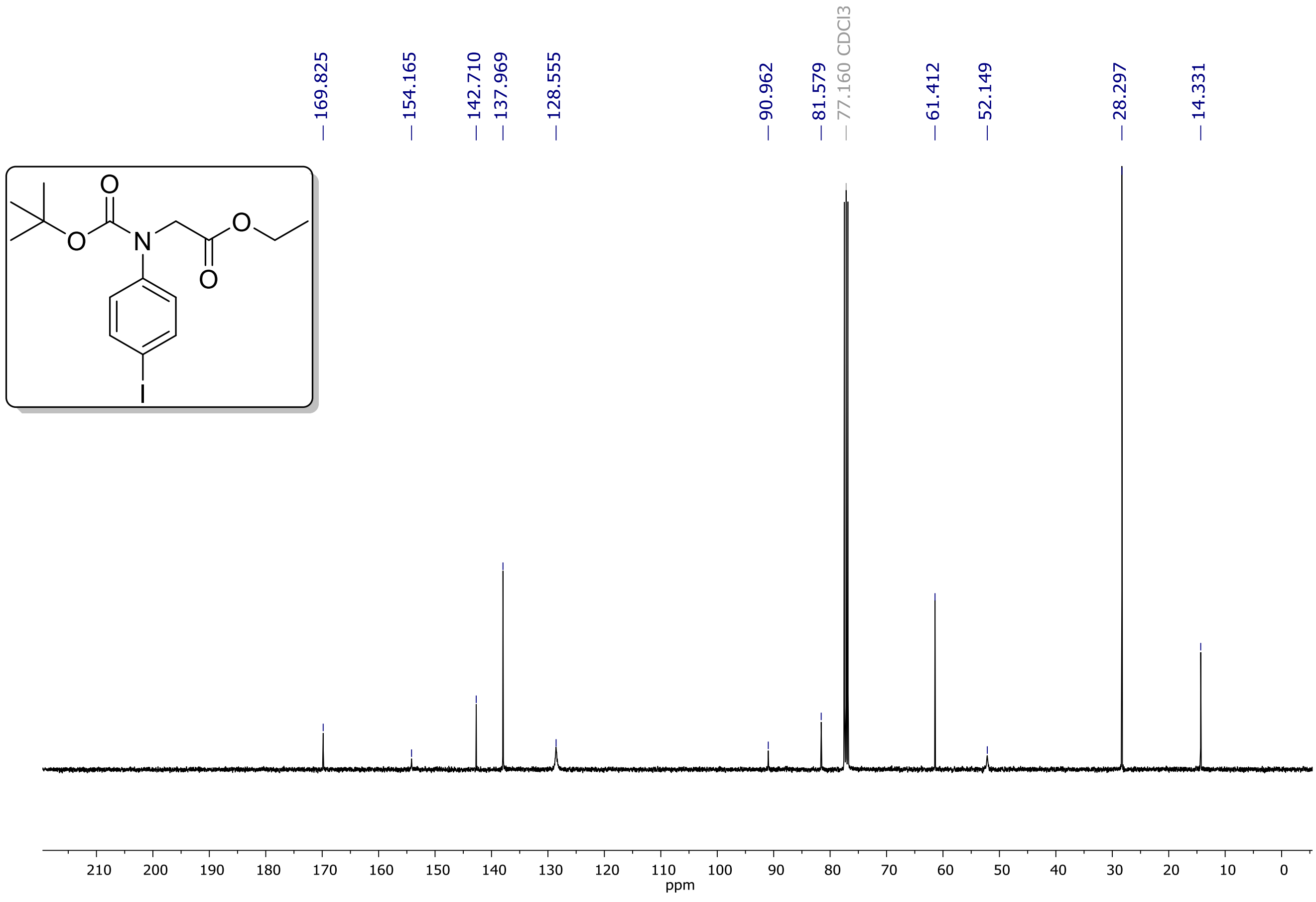

Figure S7. ^{13}C NMR (100 MHz, $CDCl_3$) spectrum of **V**.

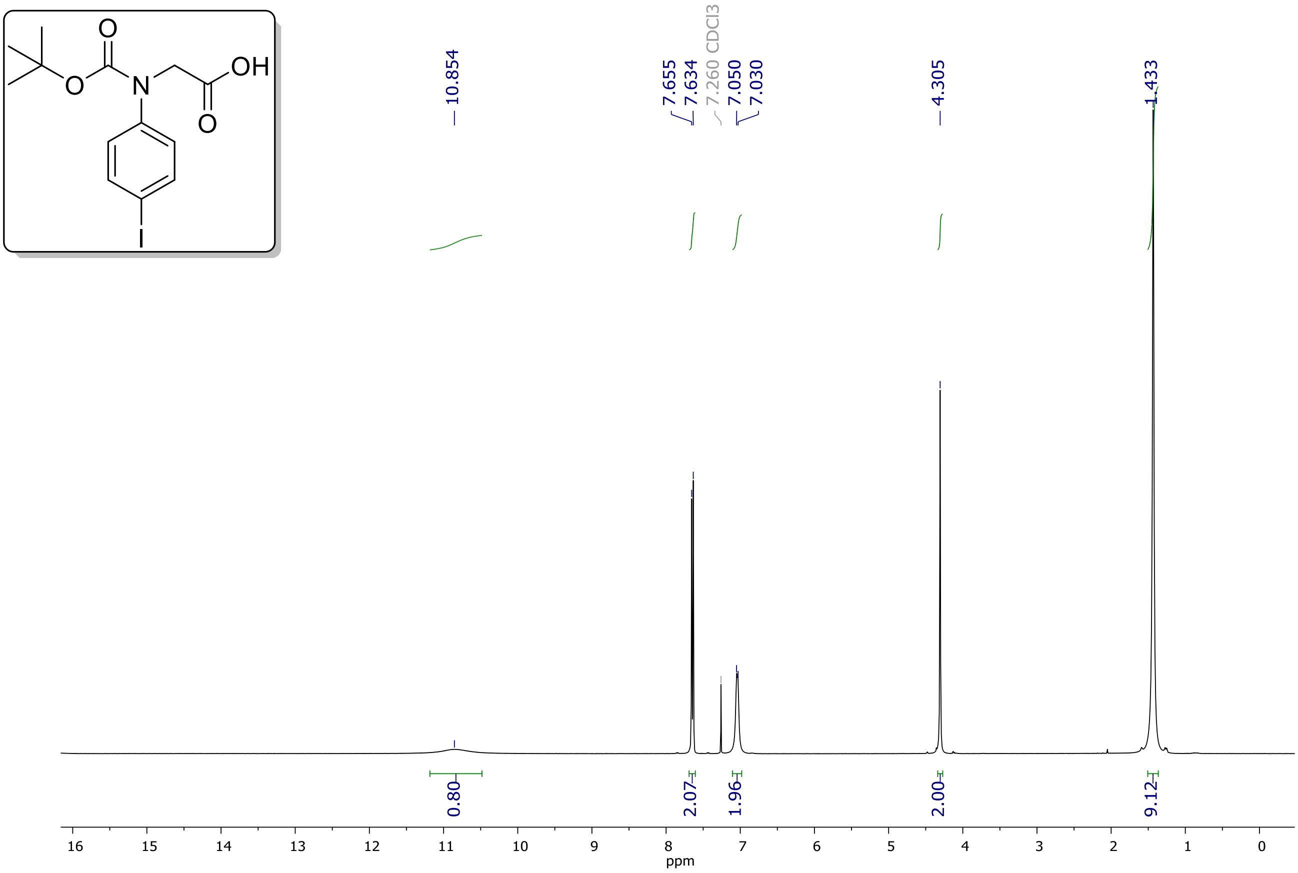

Figure S8. ^{1}H NMR (400 MHz, $CDCl_3$) spectrum of **VI**.

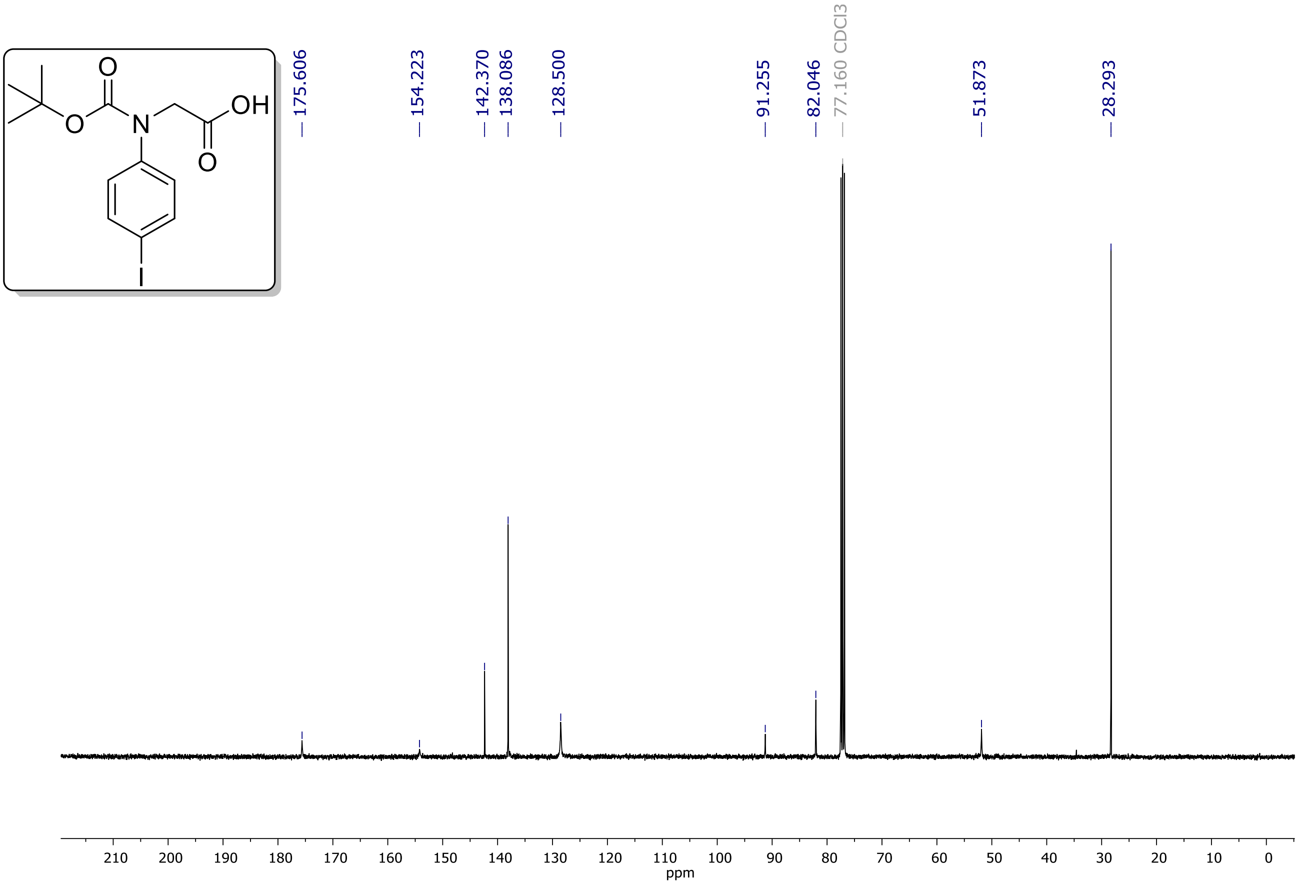

Figure S9. ^{13}C NMR (100 MHz, $CDCl_3$) spectrum of **VI**.

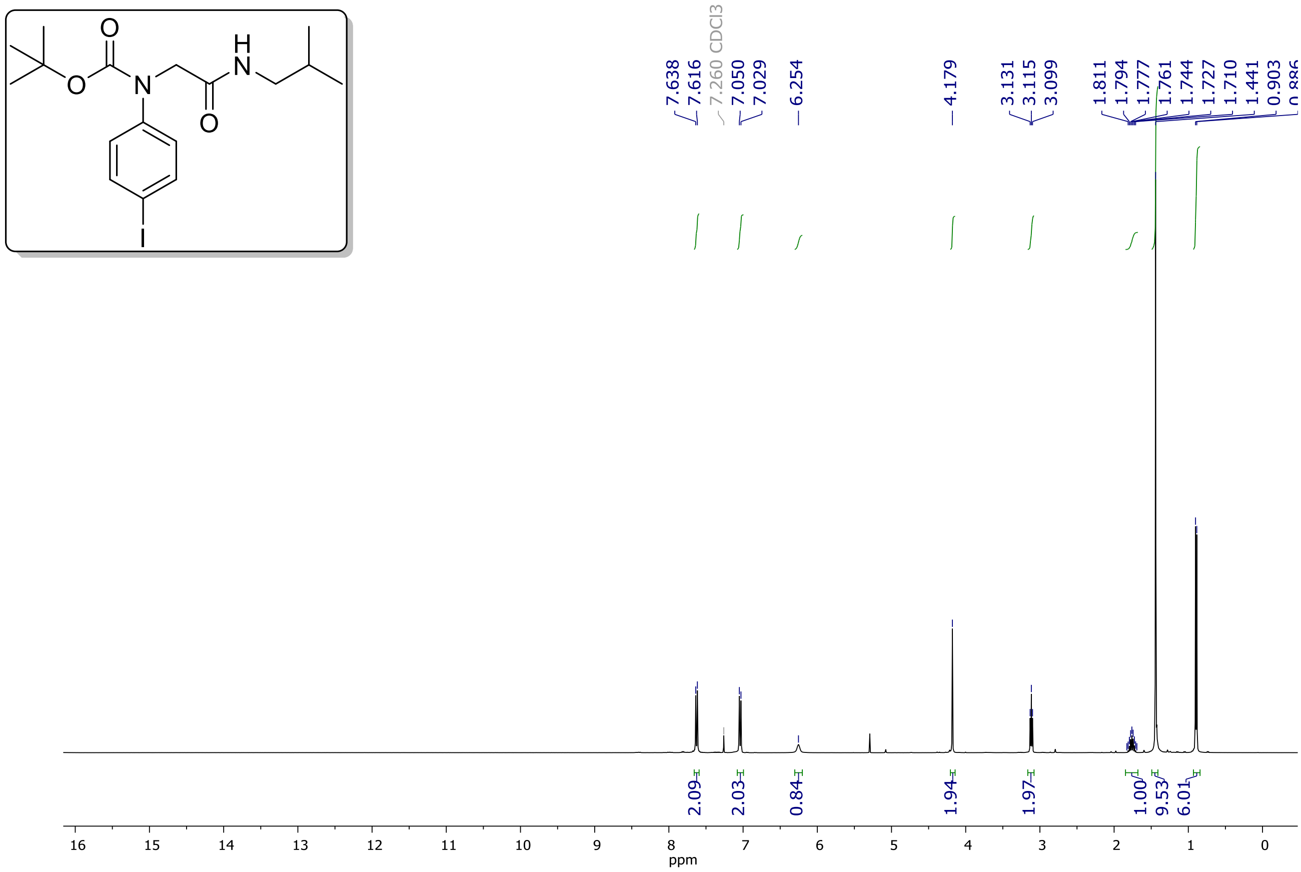

Figure S10. ^{1}H NMR (400 MHz, $CDCl_3$) spectrum of **VII**.

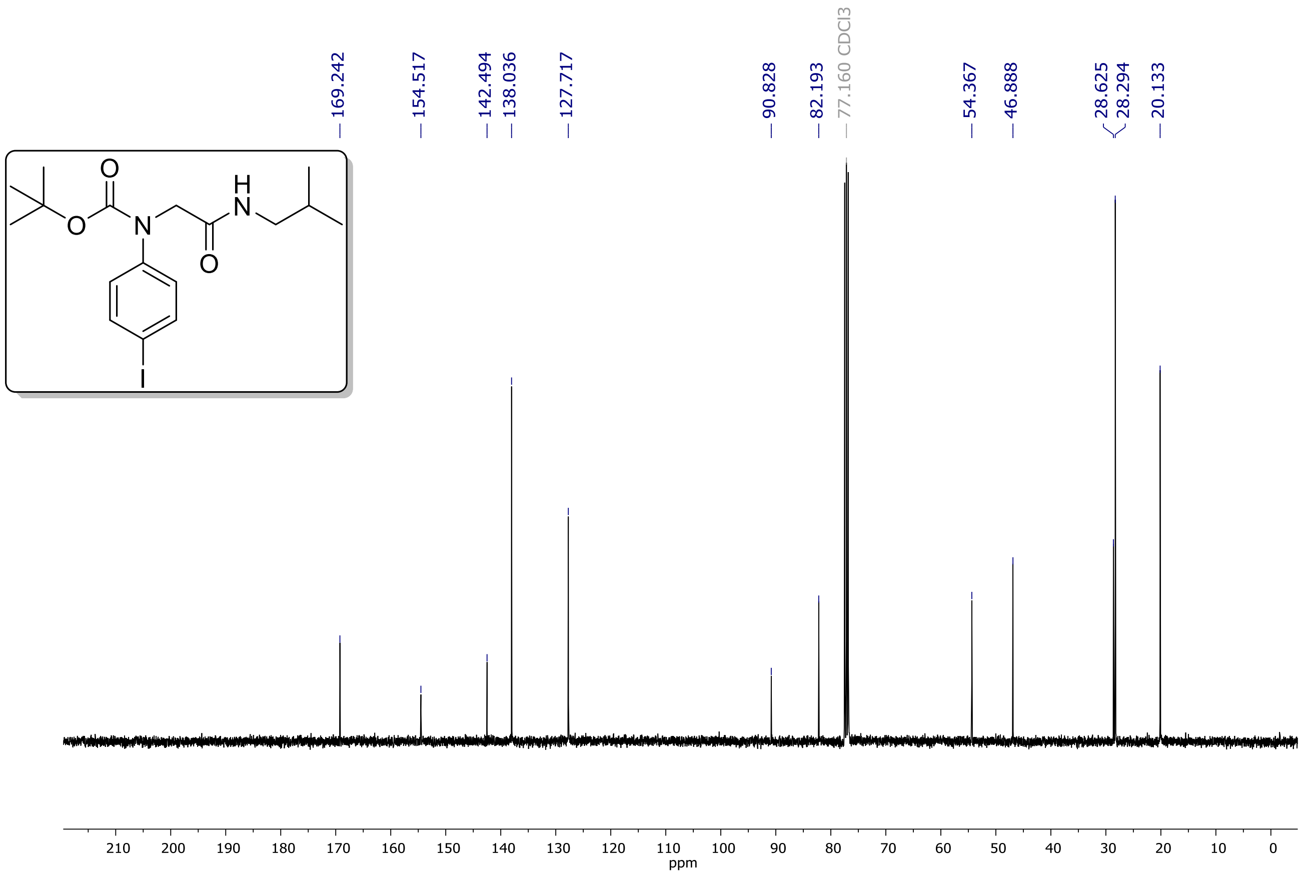

Figure S11. ^{13}C NMR (100 MHz, $CDCl_3$) spectrum of **VII**.

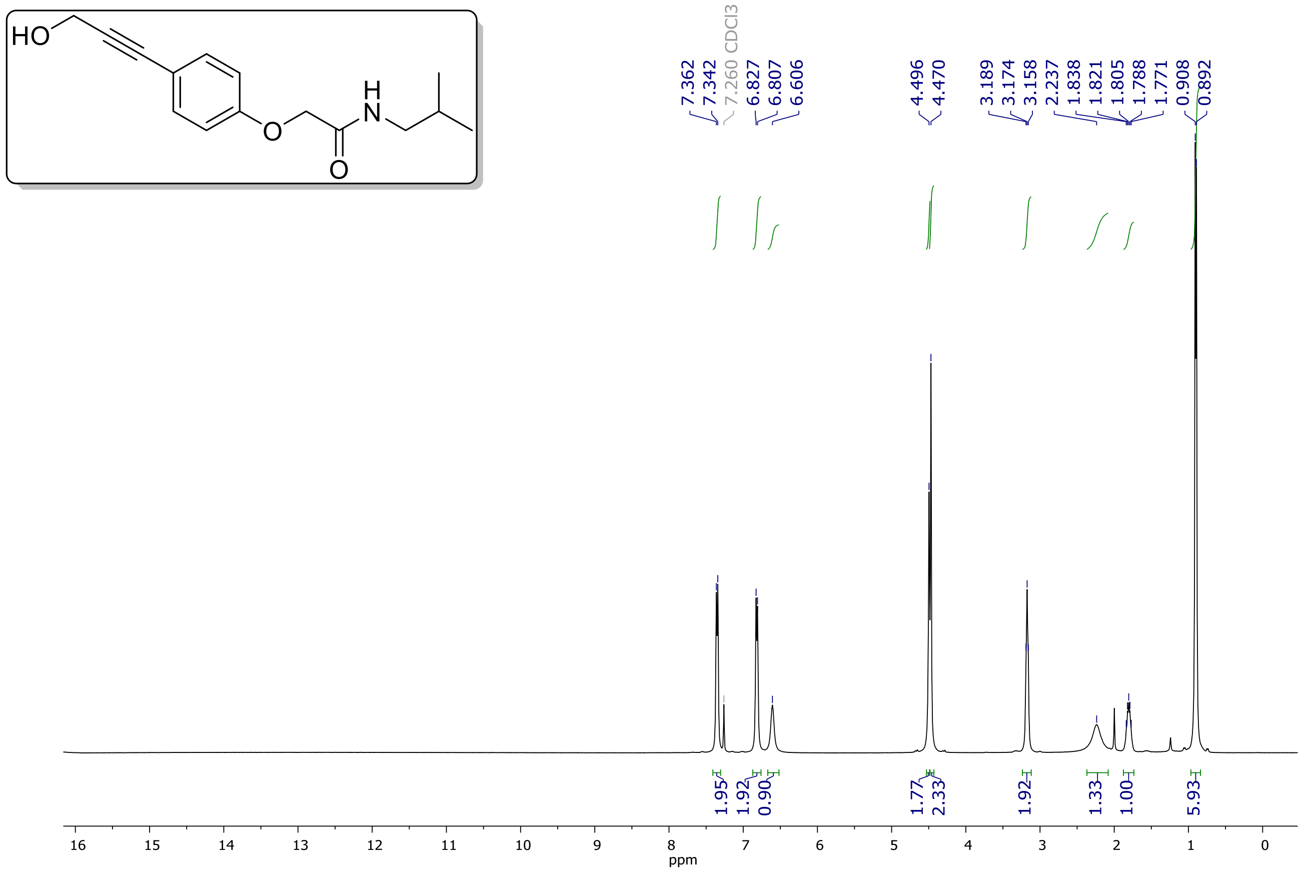

Figure S12. ^{1}H NMR (400 MHz, $CDCl_3$) spectrum of **VIIIa**.

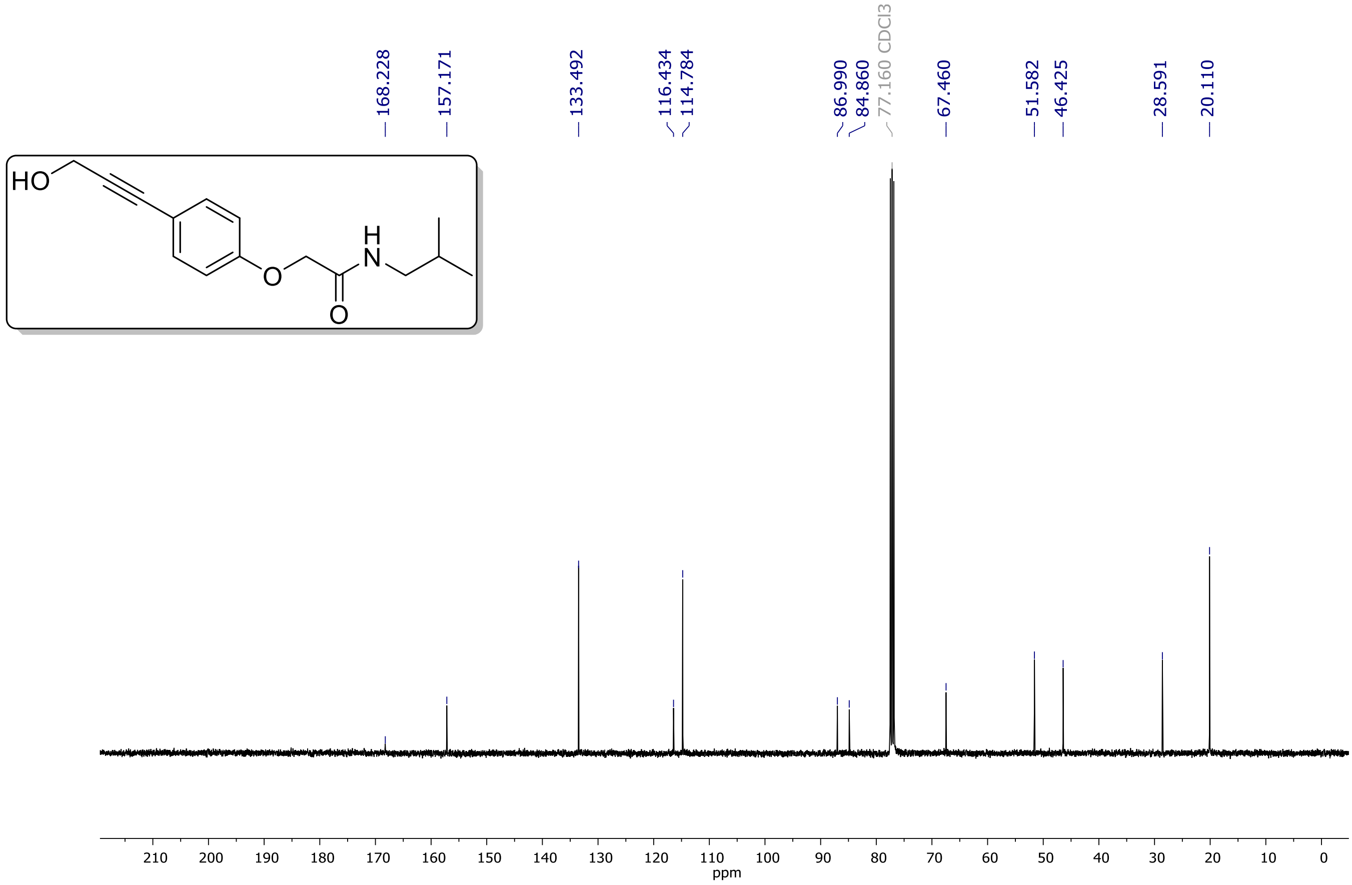

Figure S13. ^{13}C NMR (100 MHz, $CDCl_3$) spectrum of **VIIa**.

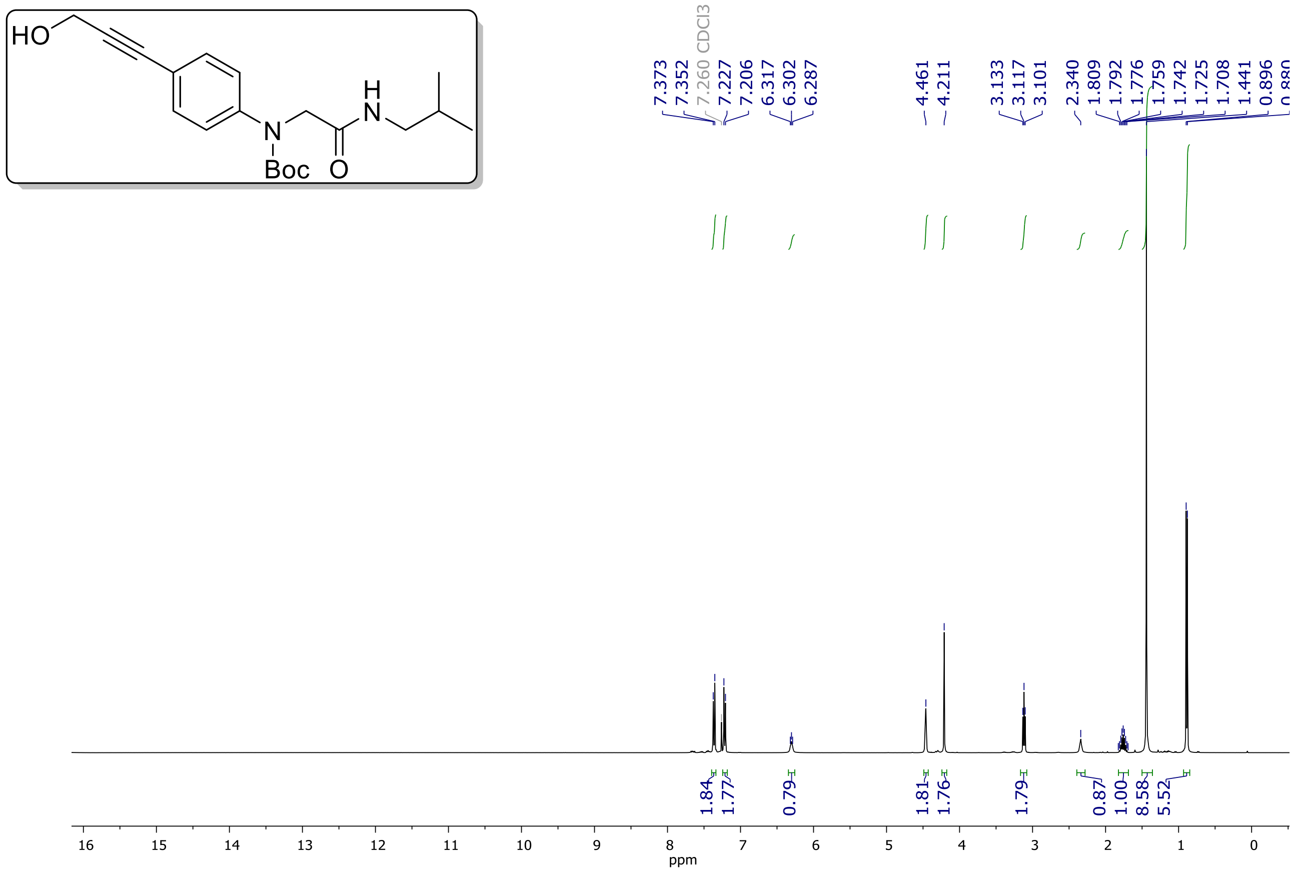

Figure S14. ^{1}H NMR (400 MHz, $CDCl_3$) spectrum of **VIIIb**.

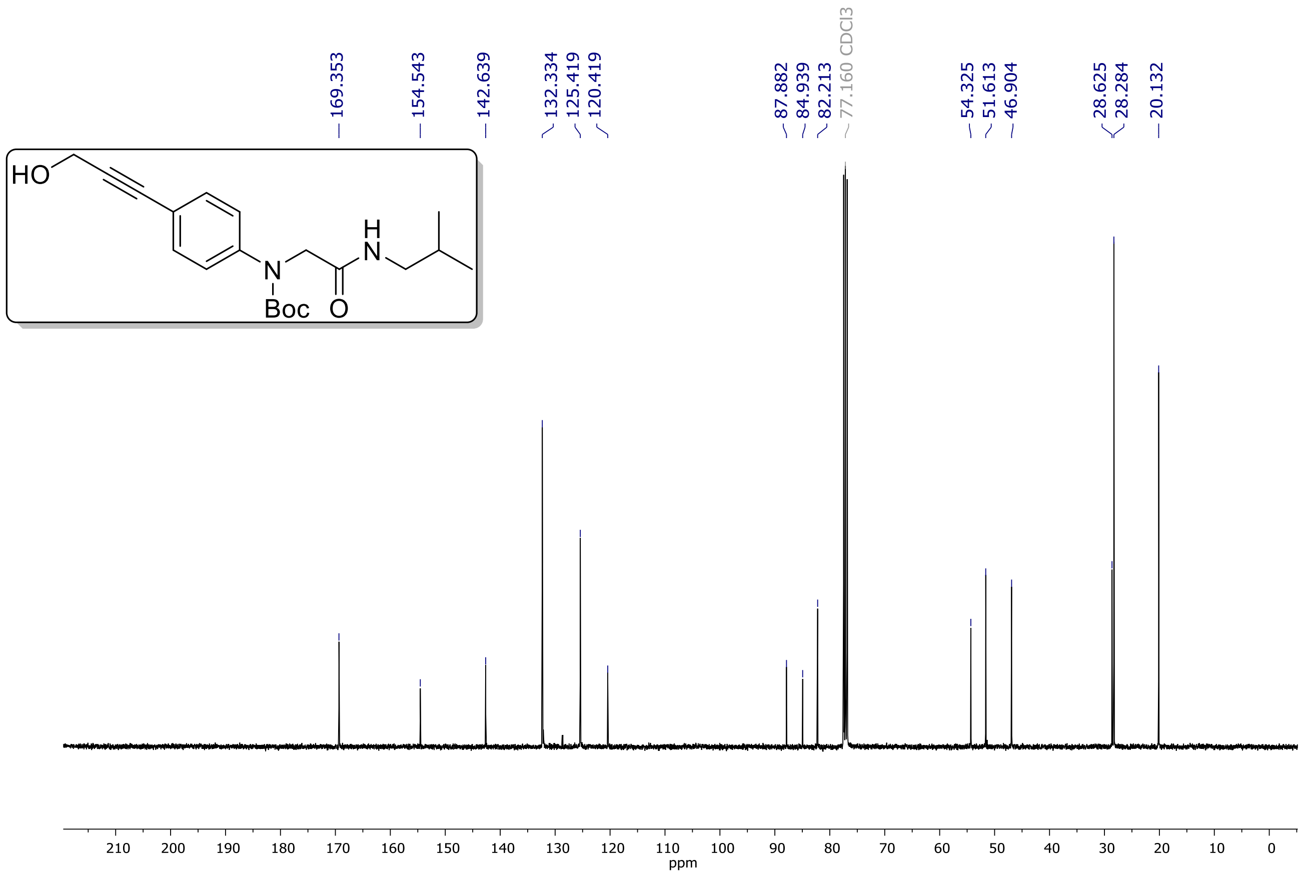

Figure S15. ^{13}C NMR (100 MHz, $CDCl_3$) spectrum of **VIIb**.

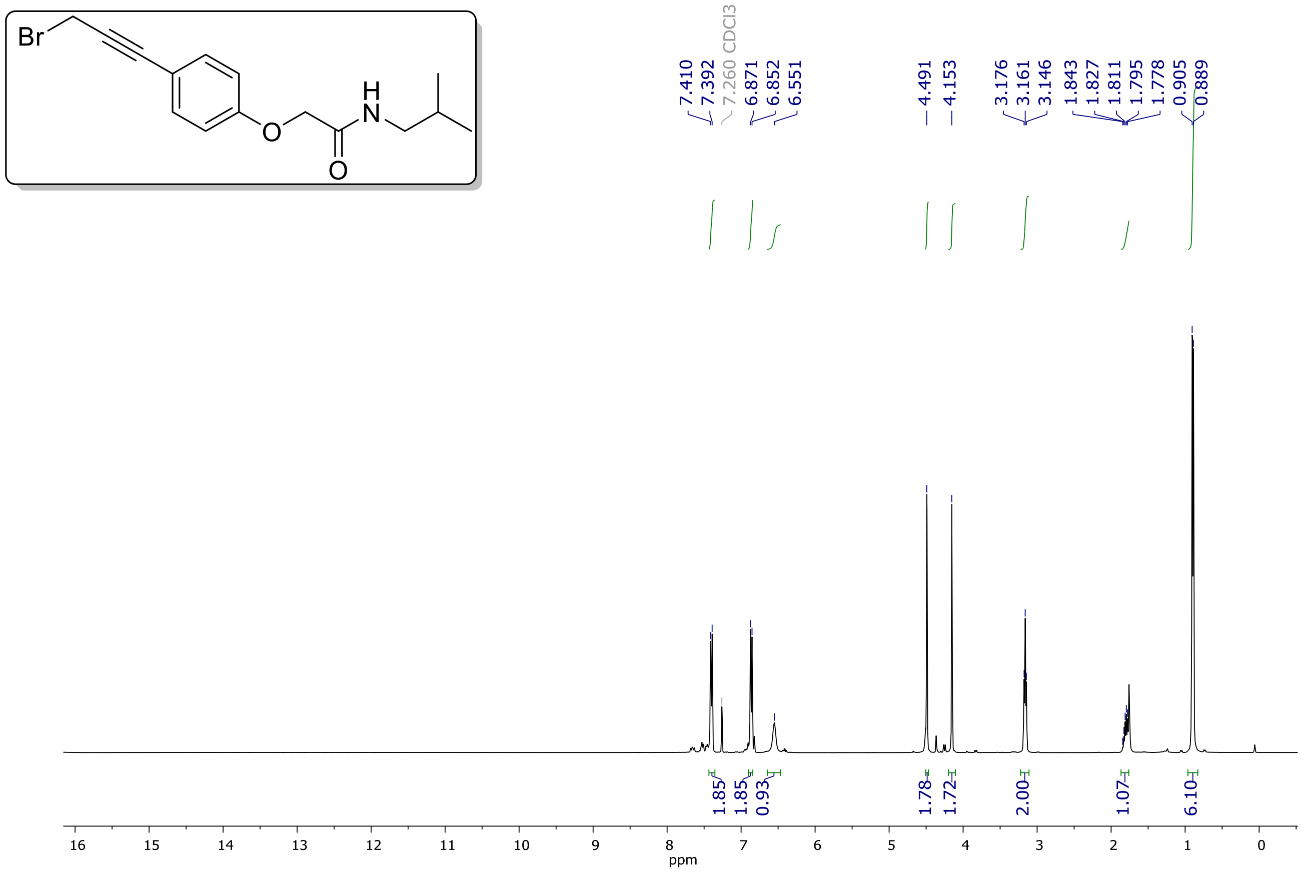

Figure S16. ^{1}H NMR (400 MHz, $CDCl_3$) spectrum of **IXa**.

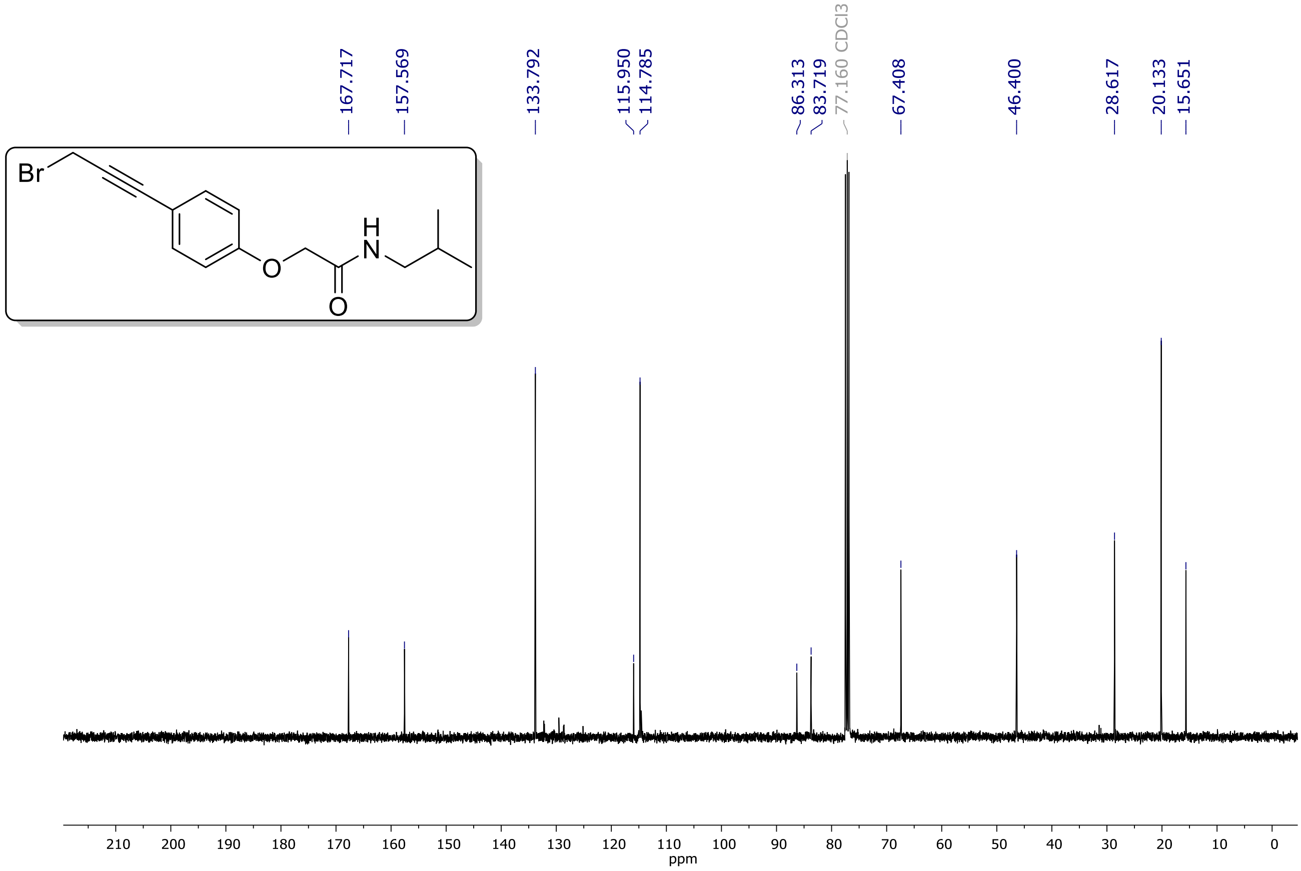

Figure S17. ^{13}C NMR (100 MHz, $CDCl_3$) spectrum of **IXa**.

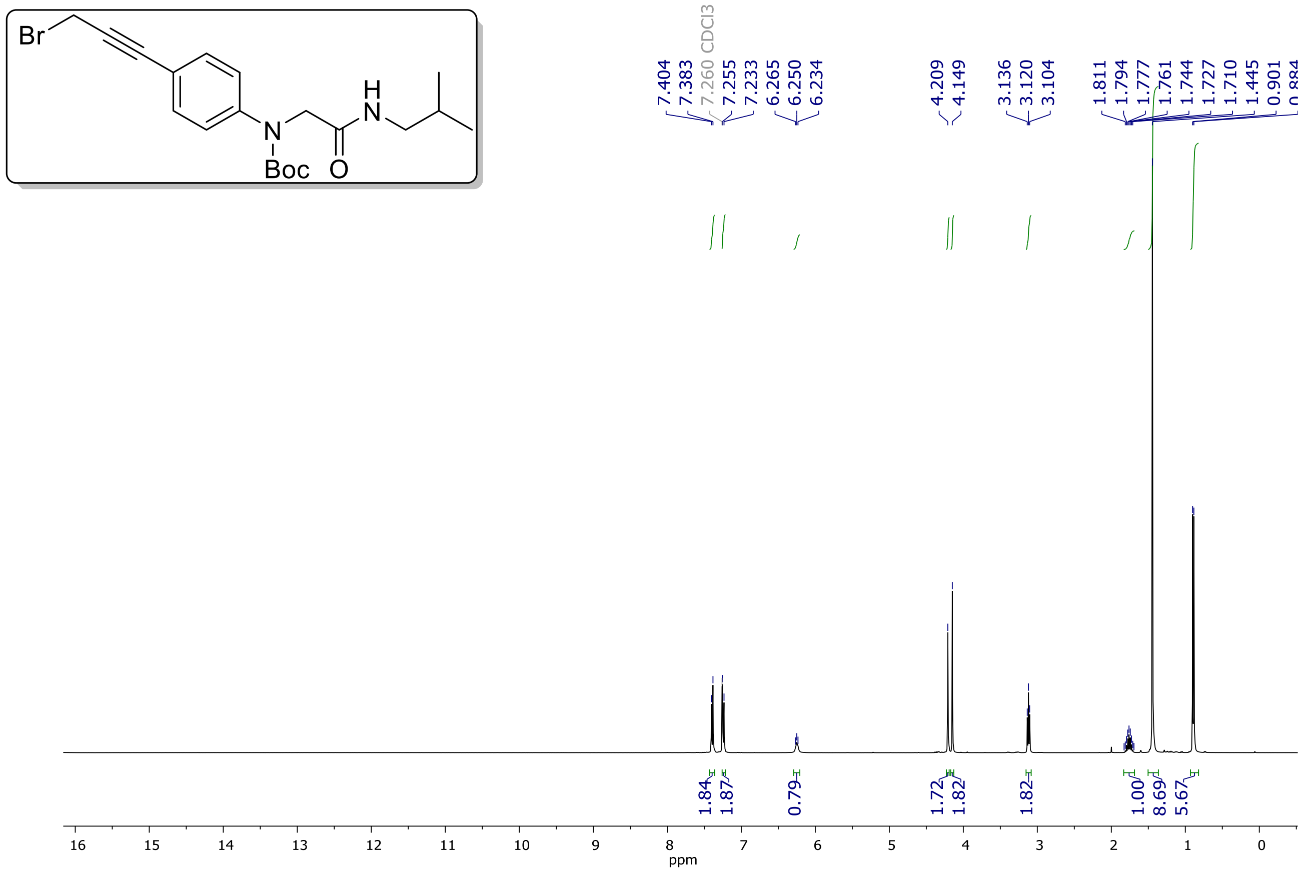

Figure S18. ^{1}H NMR (400 MHz, $CDCl_3$) spectrum of **IXb**.

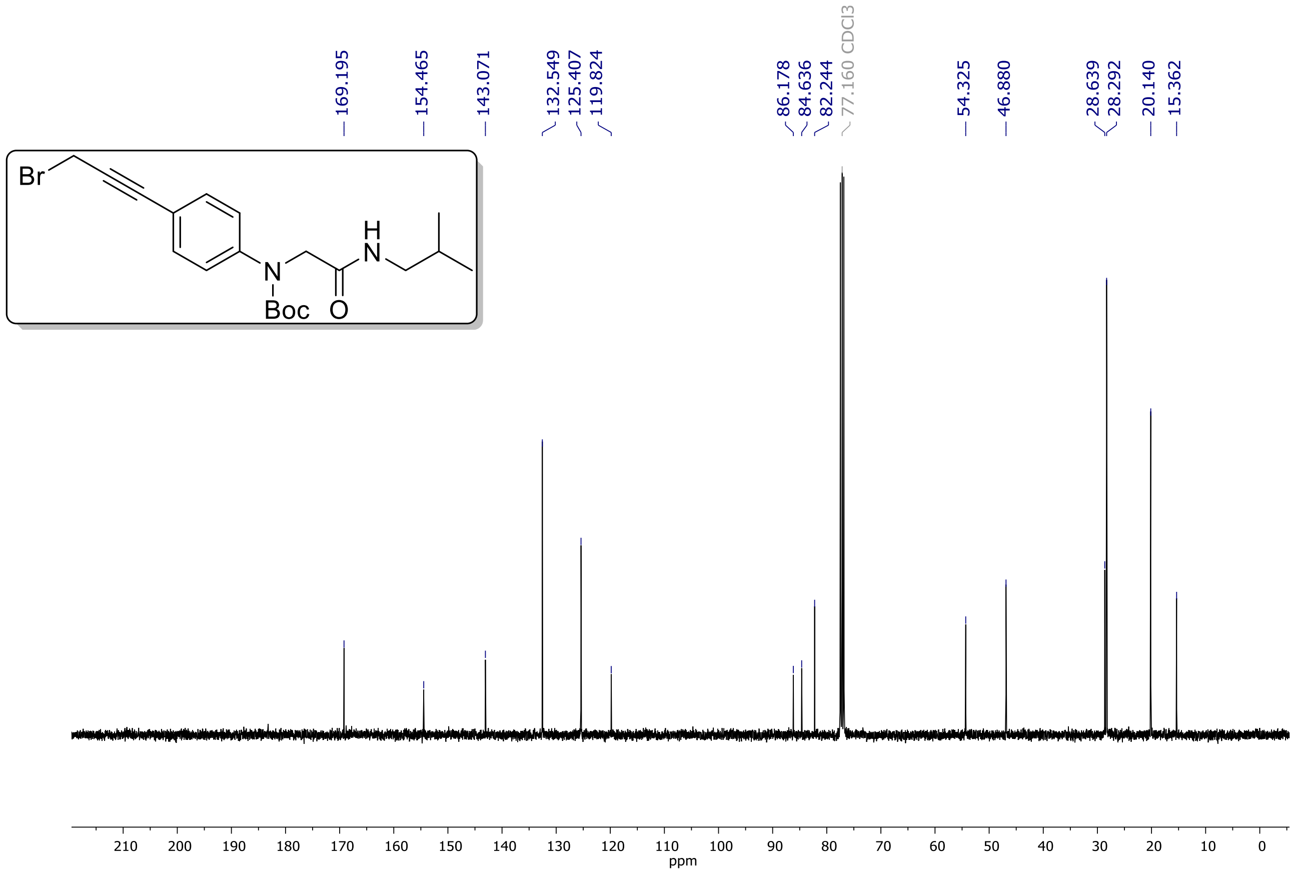

Figure S19. ^{13}C NMR (100 MHz, $CDCl_3$) spectrum of **IXb**.

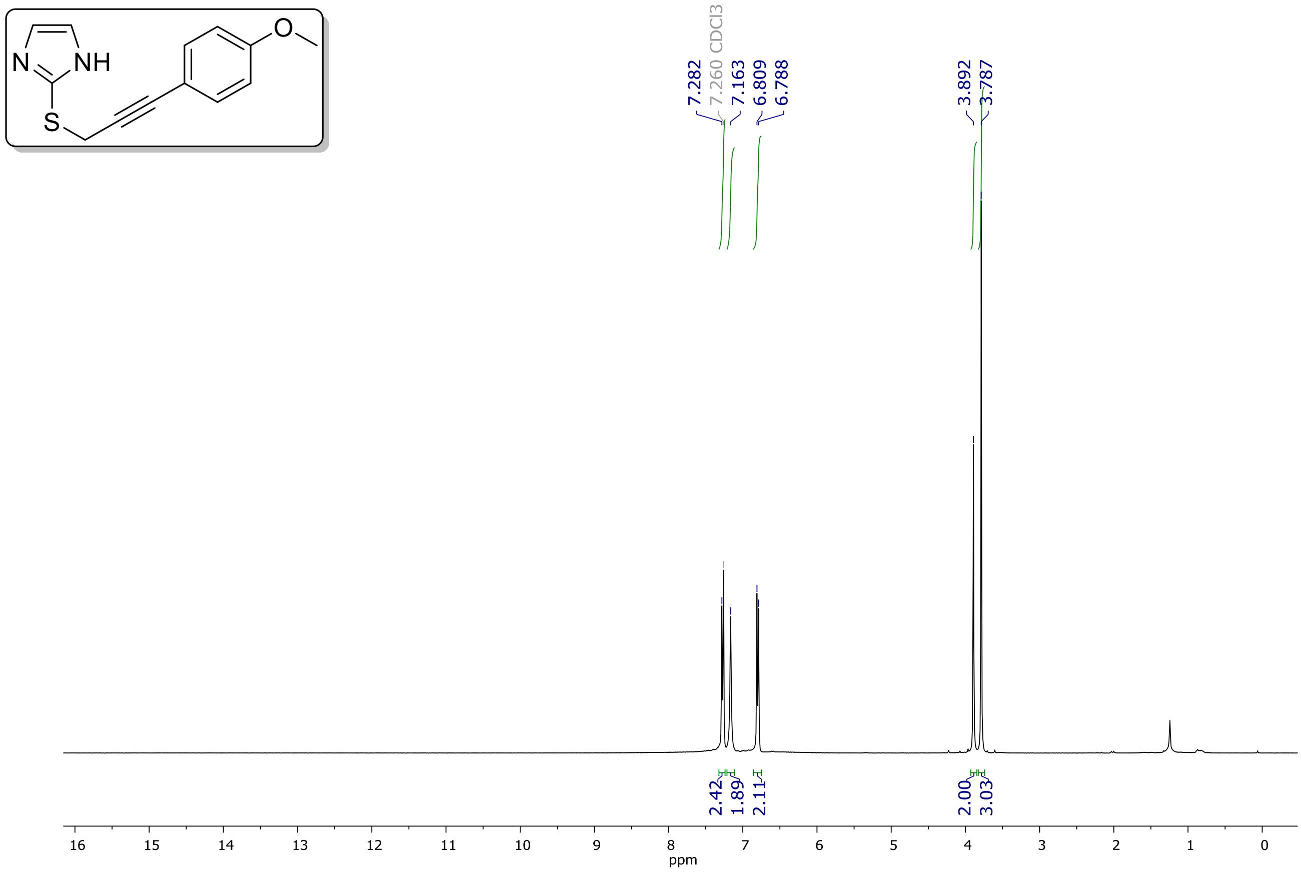

Figure S20. ^{1}H NMR (400 MHz, $CDCl_3$) spectrum of **1b**.

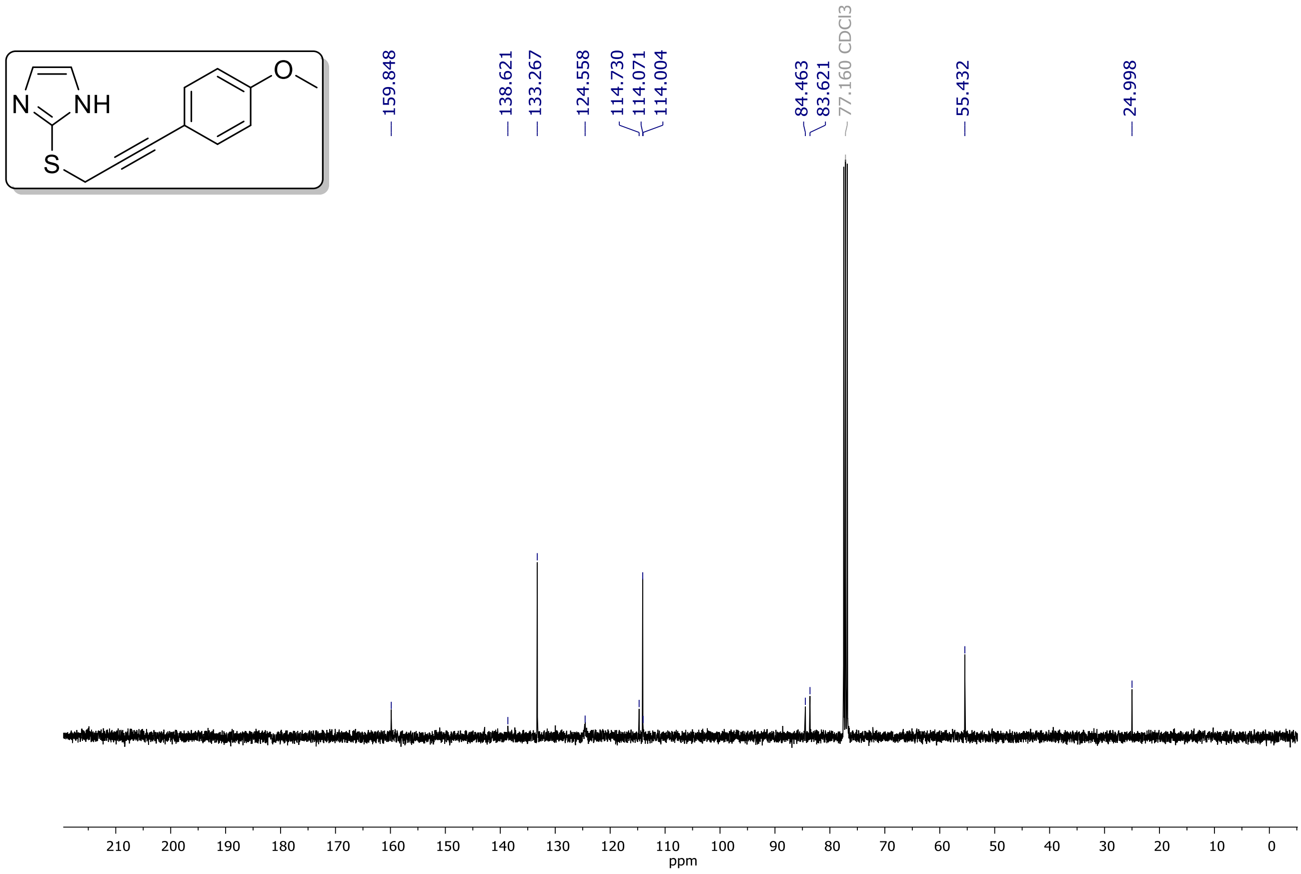

Figure S21. ^{13}C NMR (100 MHz, $CDCl_3$) spectrum of **1b**.

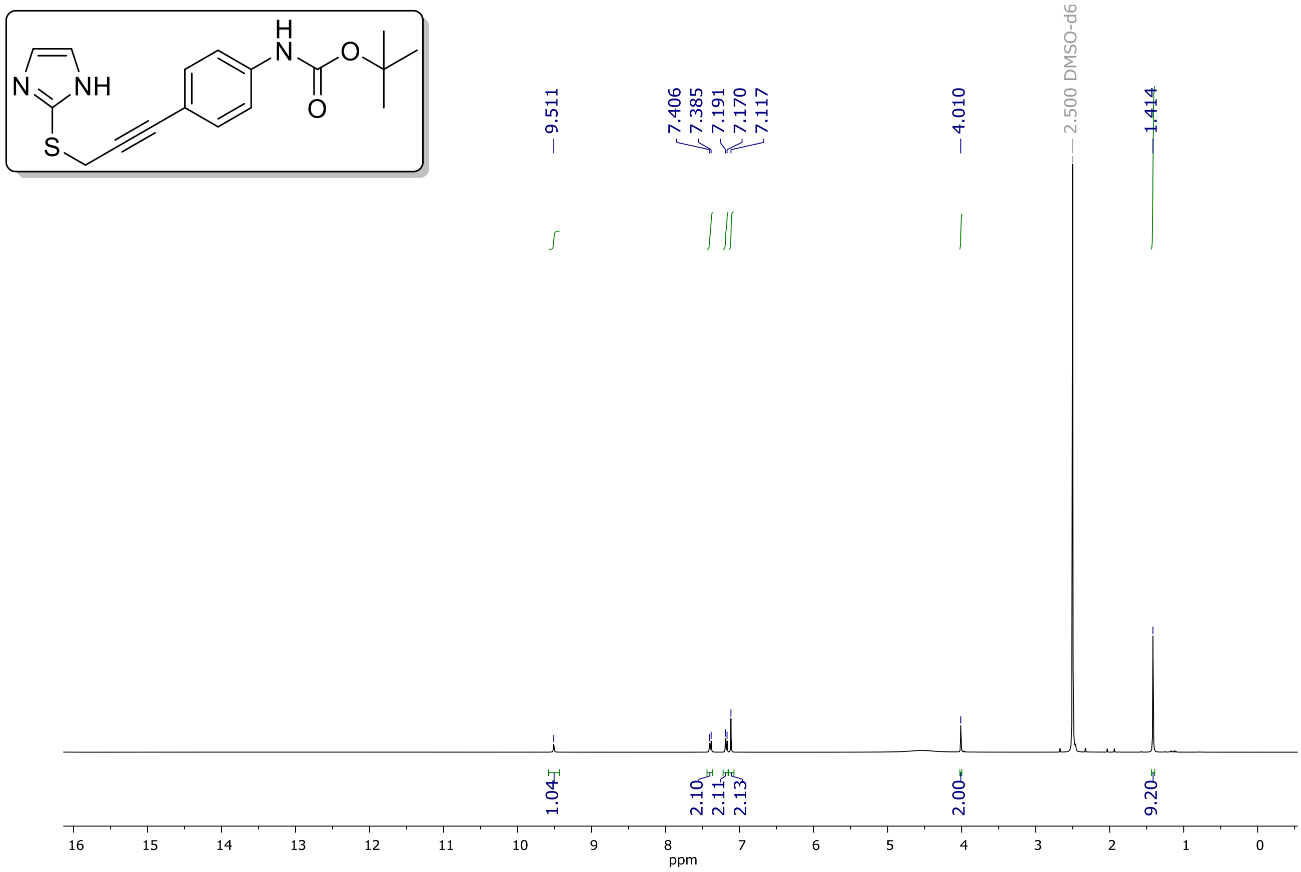

Figure S22. ^{1}H NMR (400 MHz, DMSO-d_6) spectrum of **1e**.

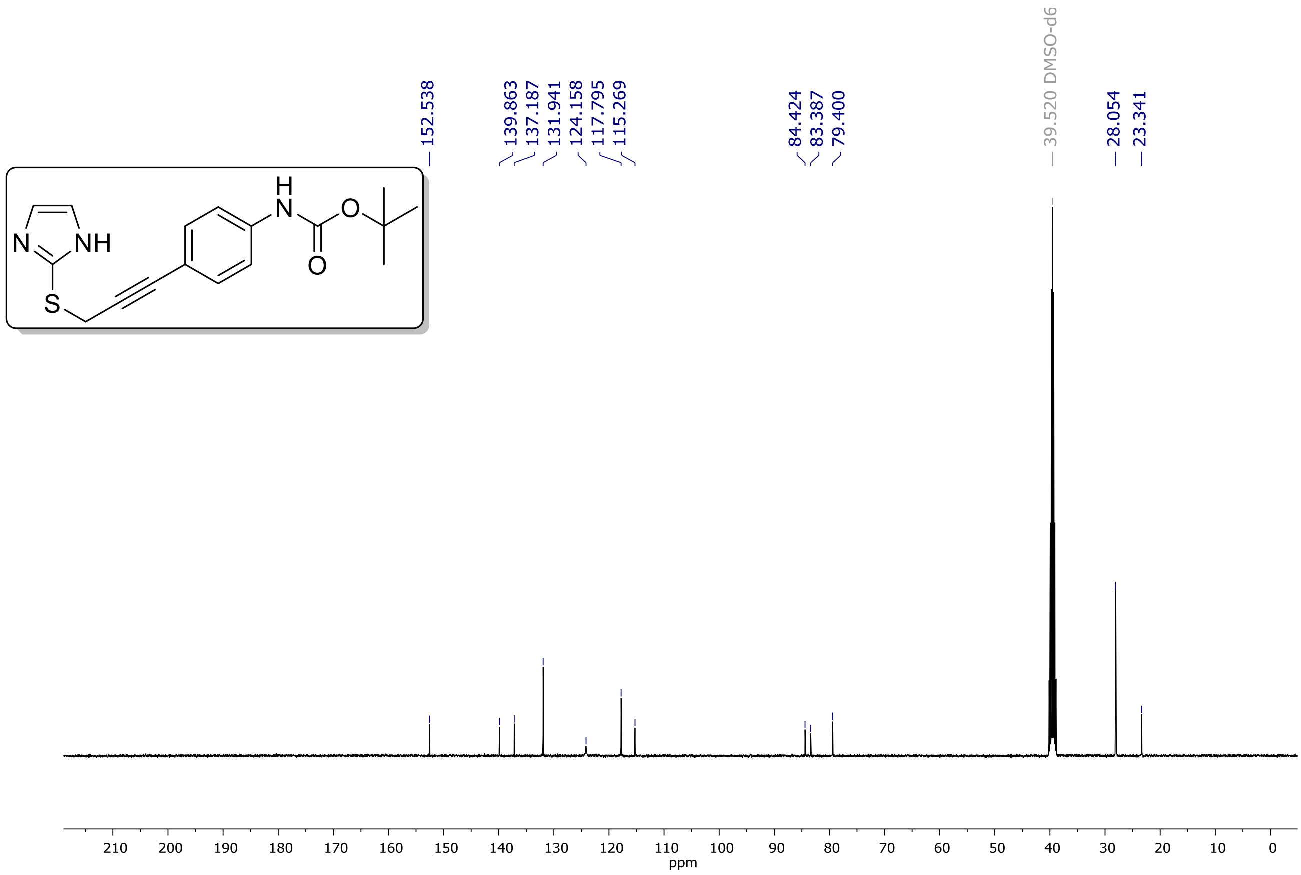

Figure S23. ^{13}C NMR (100 MHz, DMSO-d_6) spectrum of **1e**.

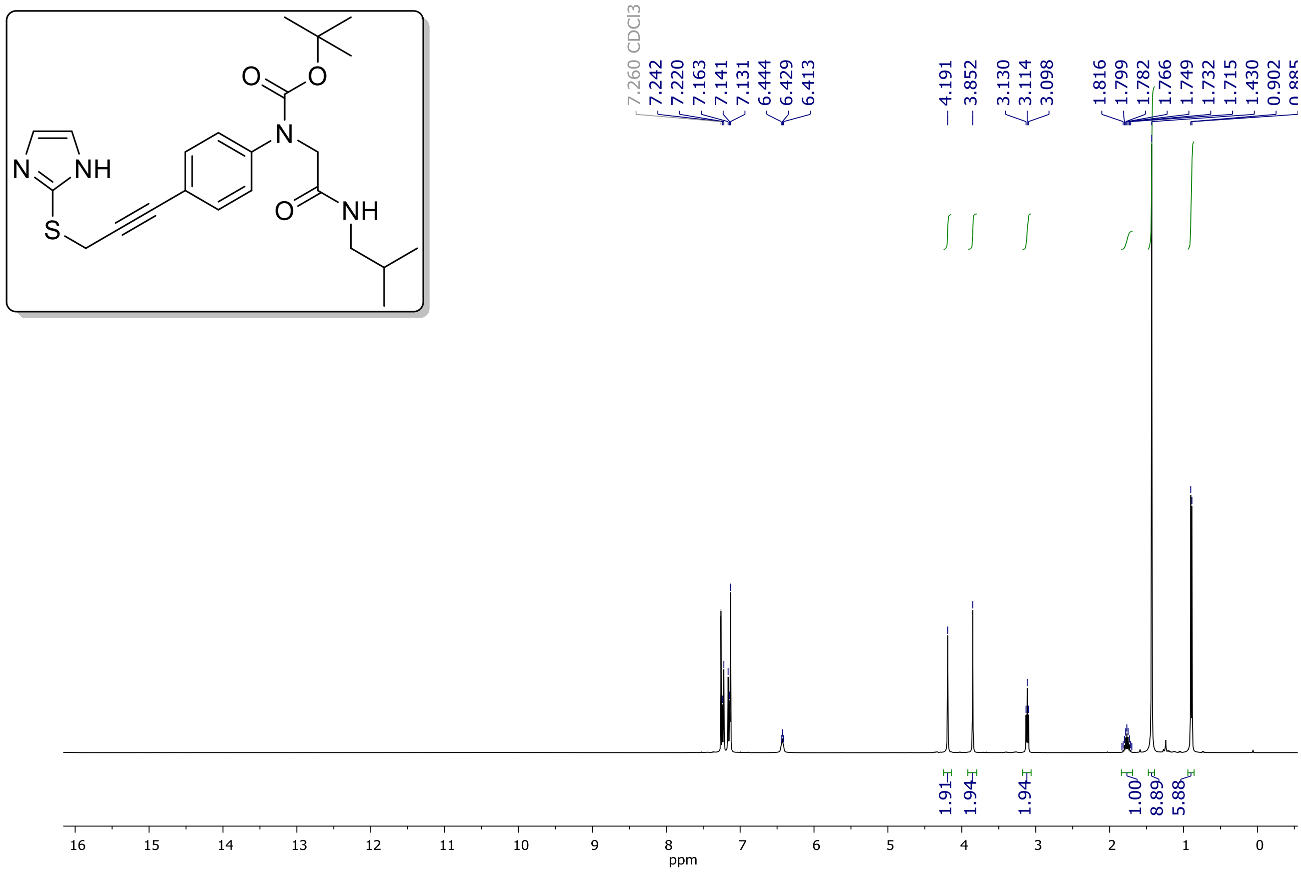

Figure S24. ^{1}H NMR (400 MHz, $CDCl_3$) spectrum of **1f**.

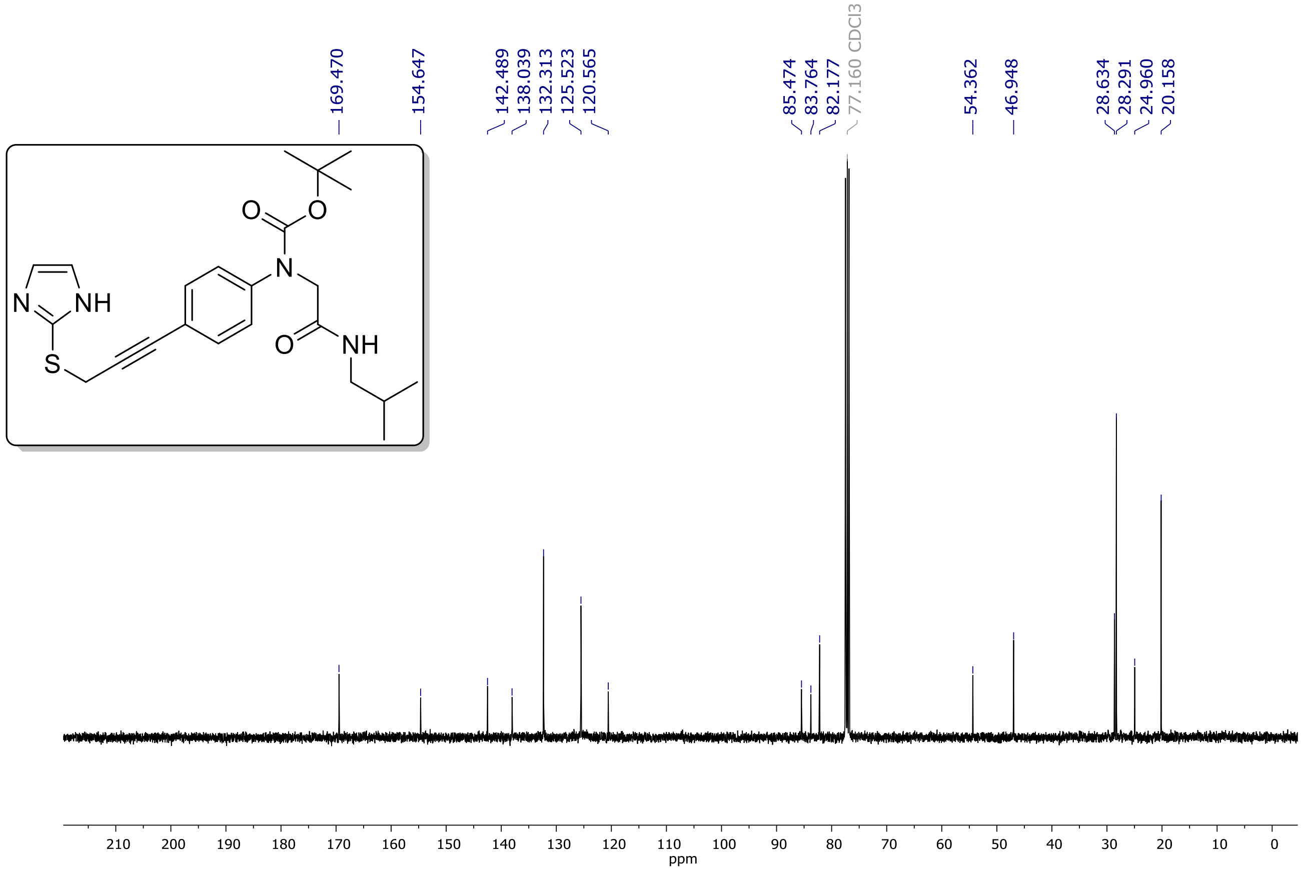

Figure S25. ^{13}C NMR (100 MHz, $CDCl_3$) spectrum of **1f**.

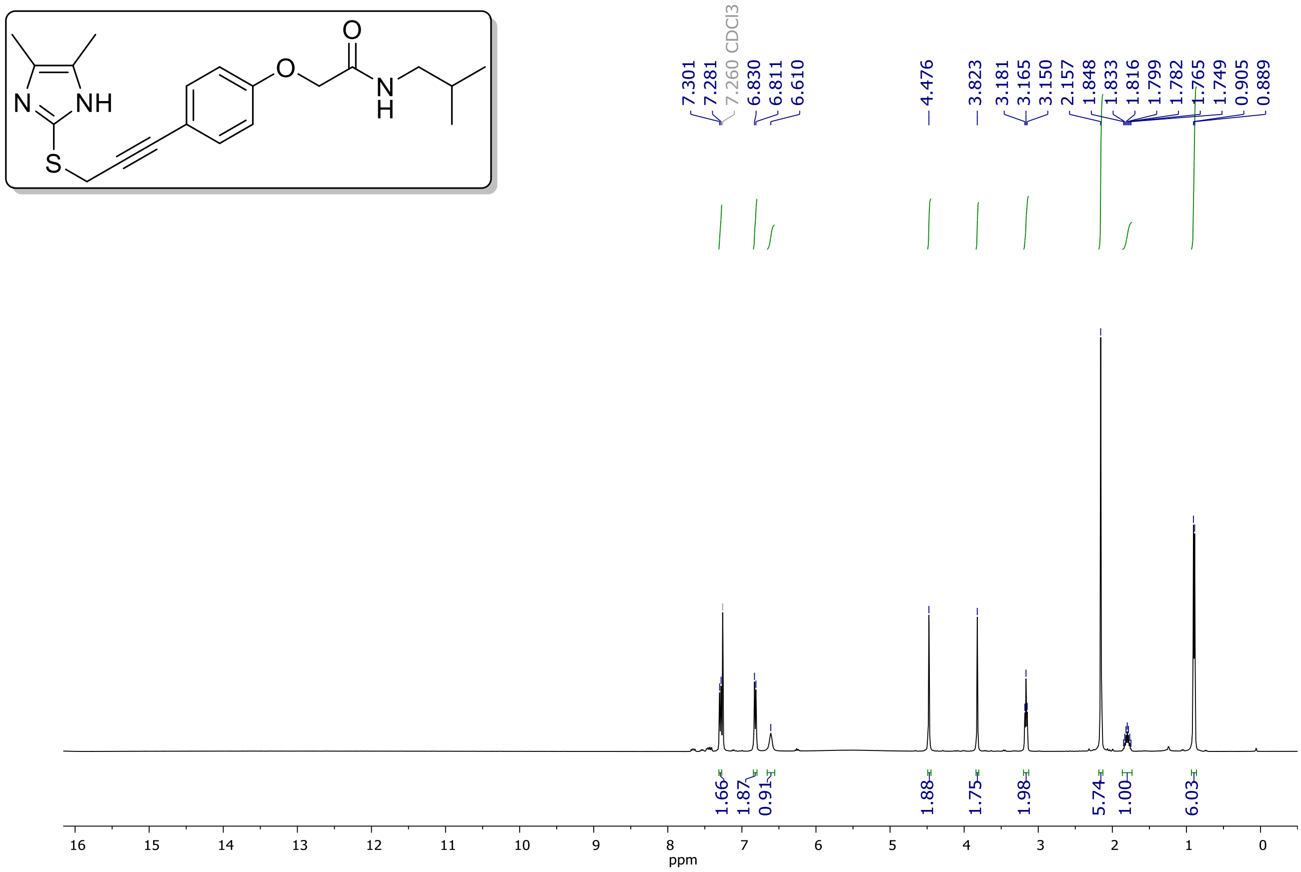

Figure S26. ^{1}H NMR (400 MHz, $CDCl_3$) spectrum of **1h**.

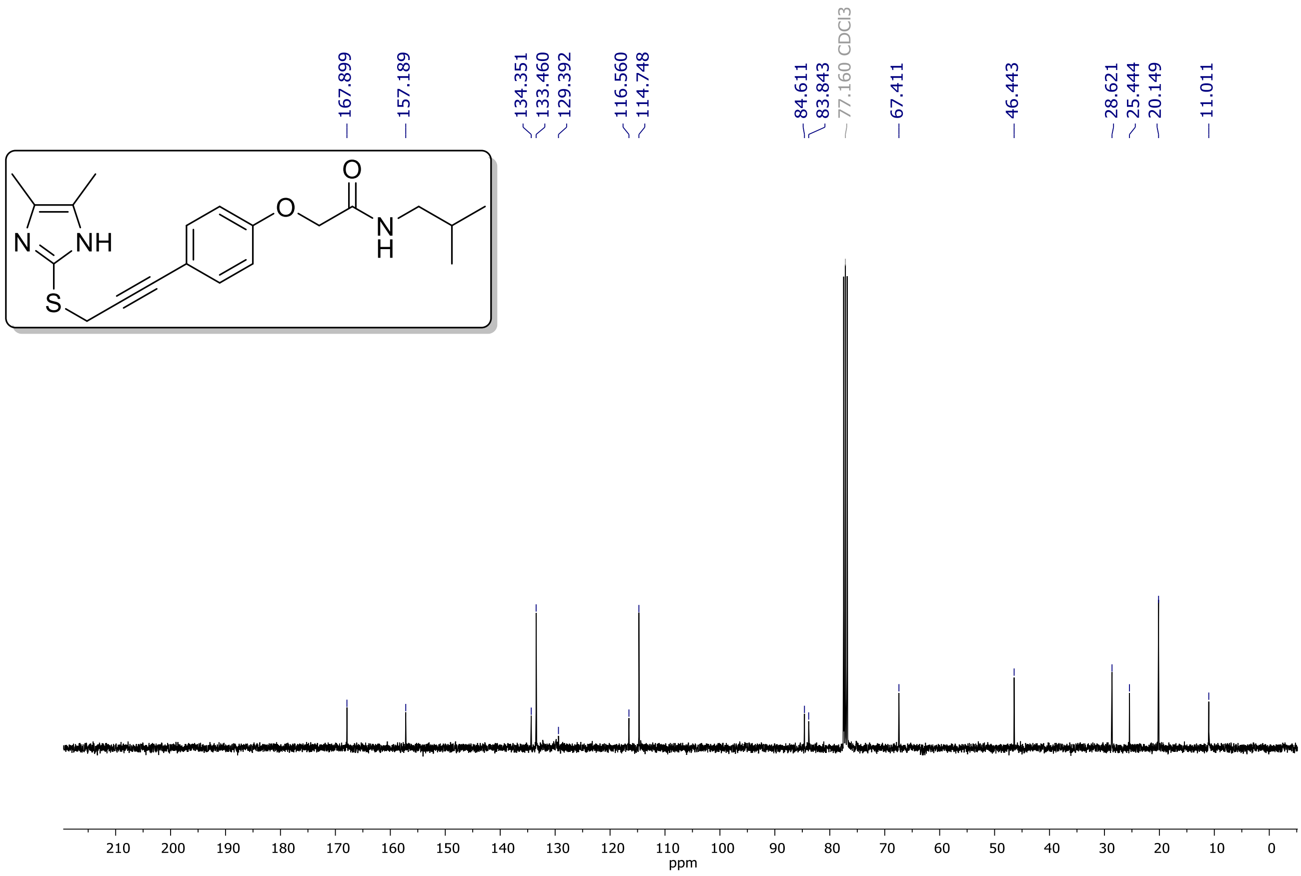

Figure S27. ^{13}C NMR (100 MHz, $CDCl_3$) spectrum of **1h**.

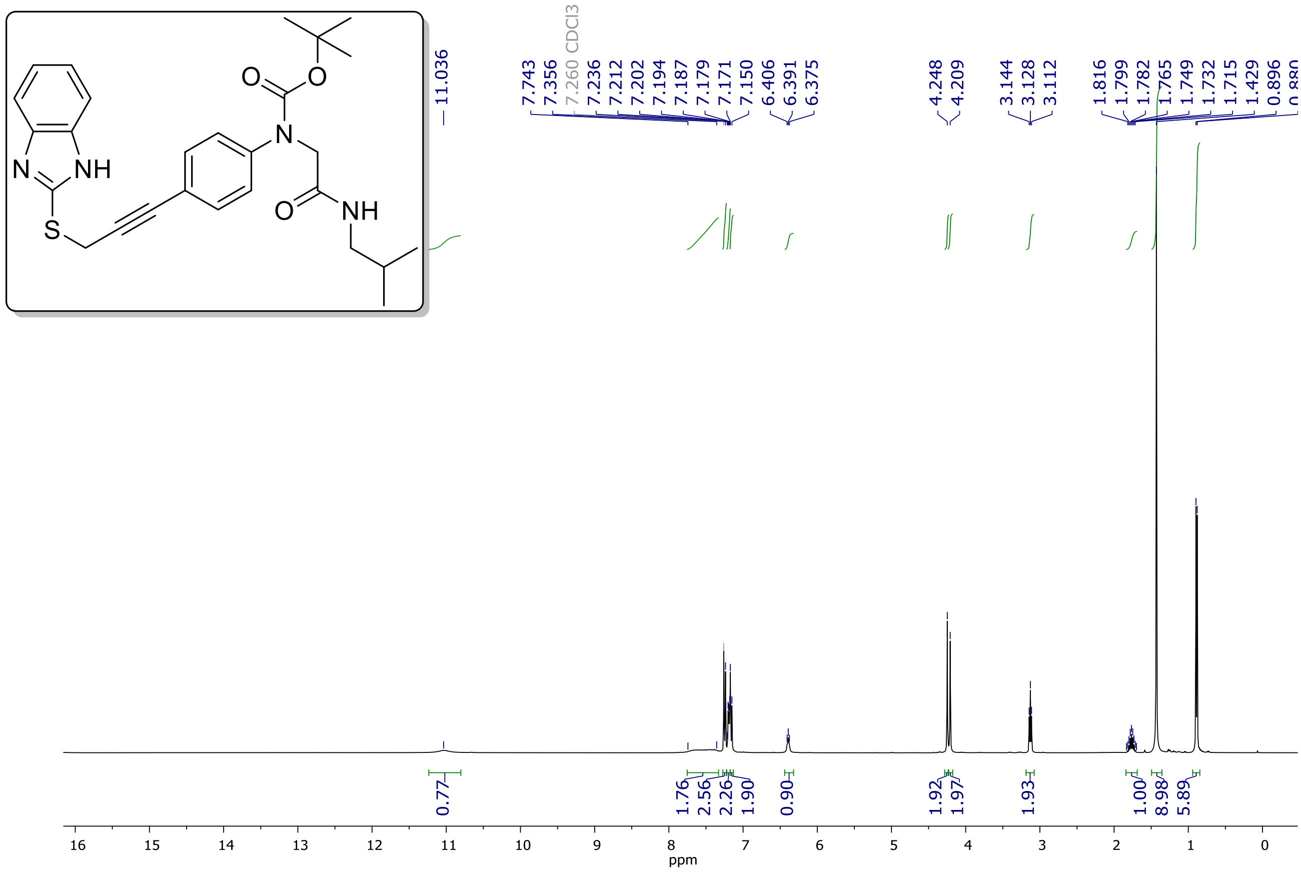

Figure S28. ^{1}H NMR (400 MHz, $CDCl_3$) spectrum of **1j**.

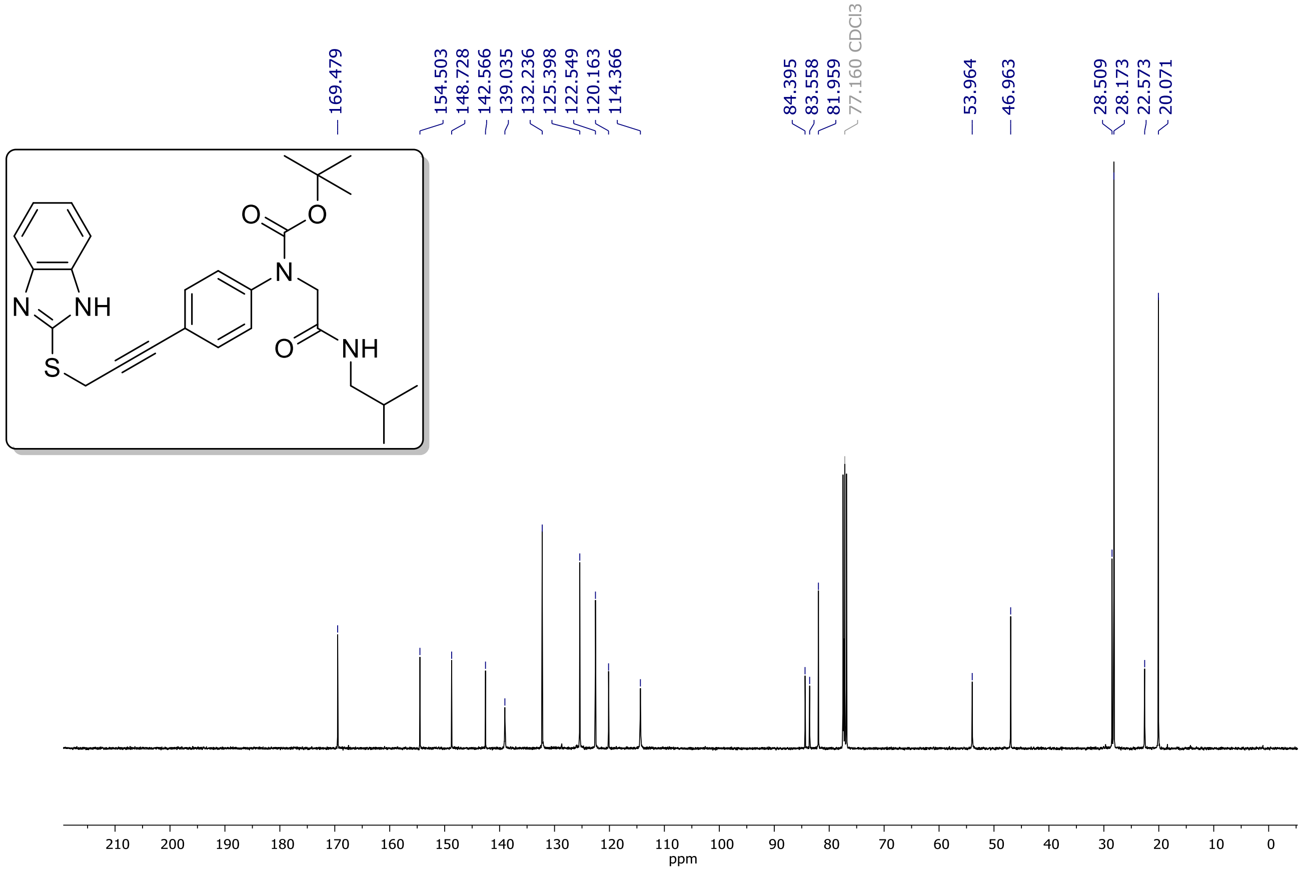

Figure S29. ^{13}C NMR (100 MHz, $CDCl_3$) spectrum of **1j**.

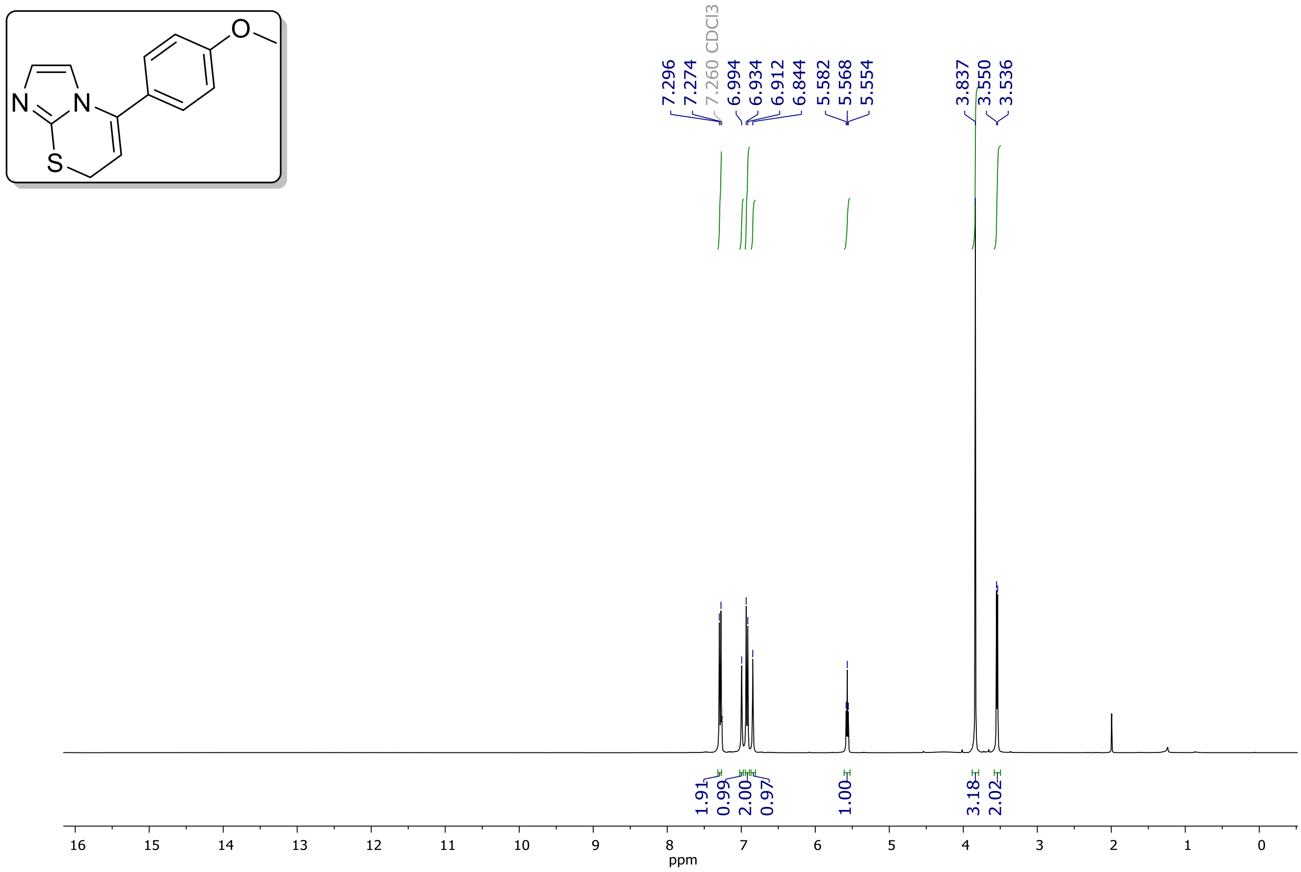

Figure S30. ^{1}H NMR (400 MHz, $CDCl_3$) spectrum of **2b**.

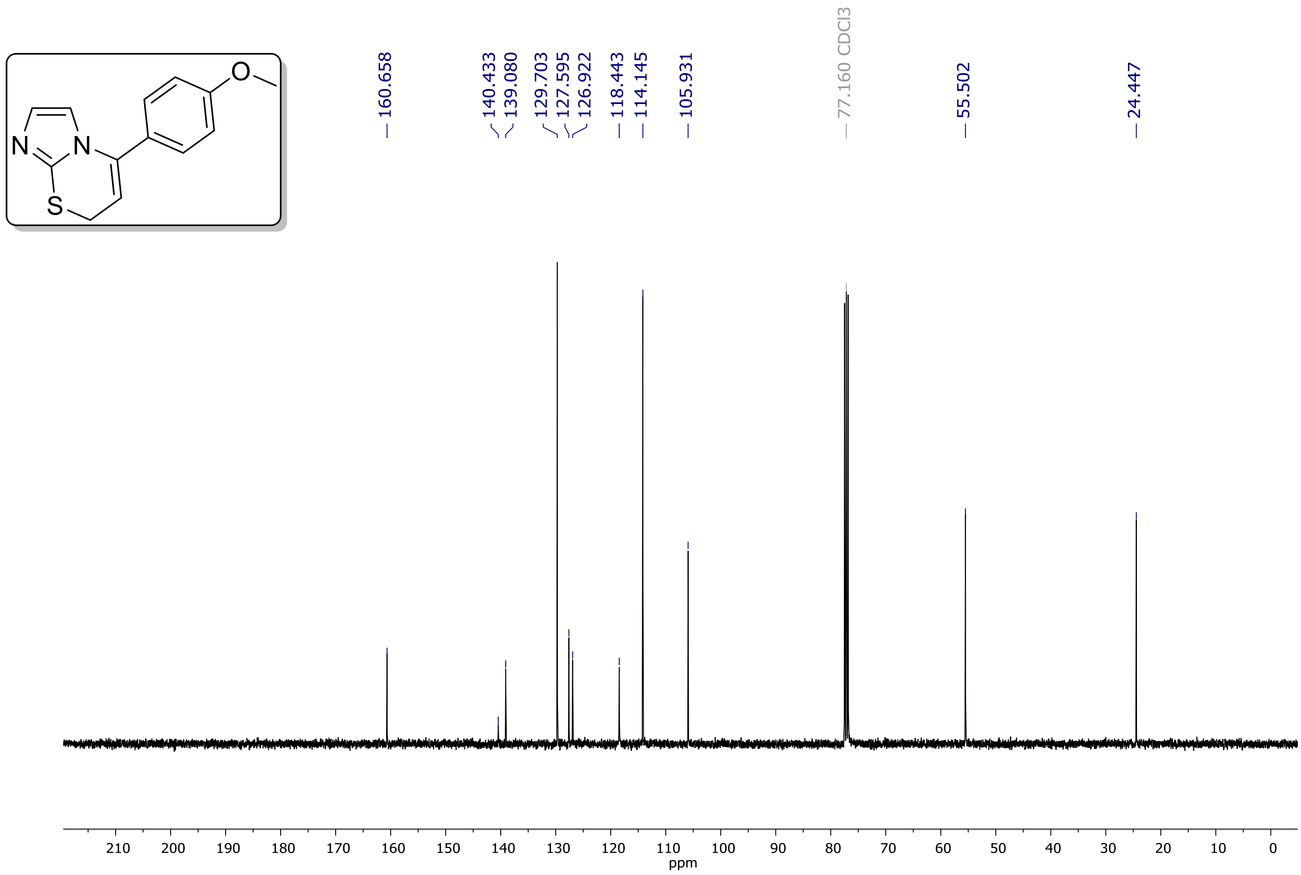

Figure S31. ^{13}C NMR (100 MHz, $CDCl_3$) spectrum of **2b**.

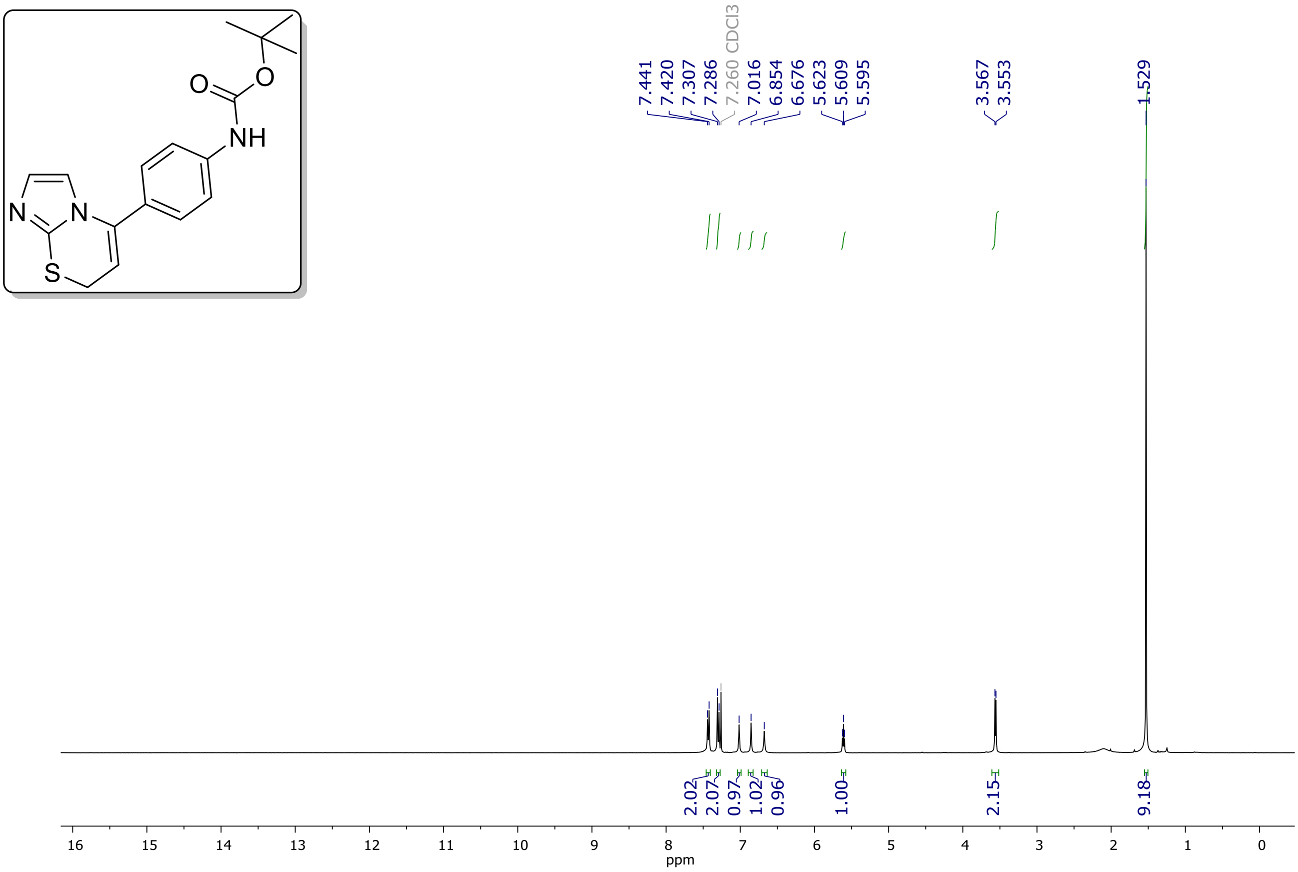

Figure S32. ^{1}H NMR (400 MHz, $CDCl_3$) spectrum of **2e**.

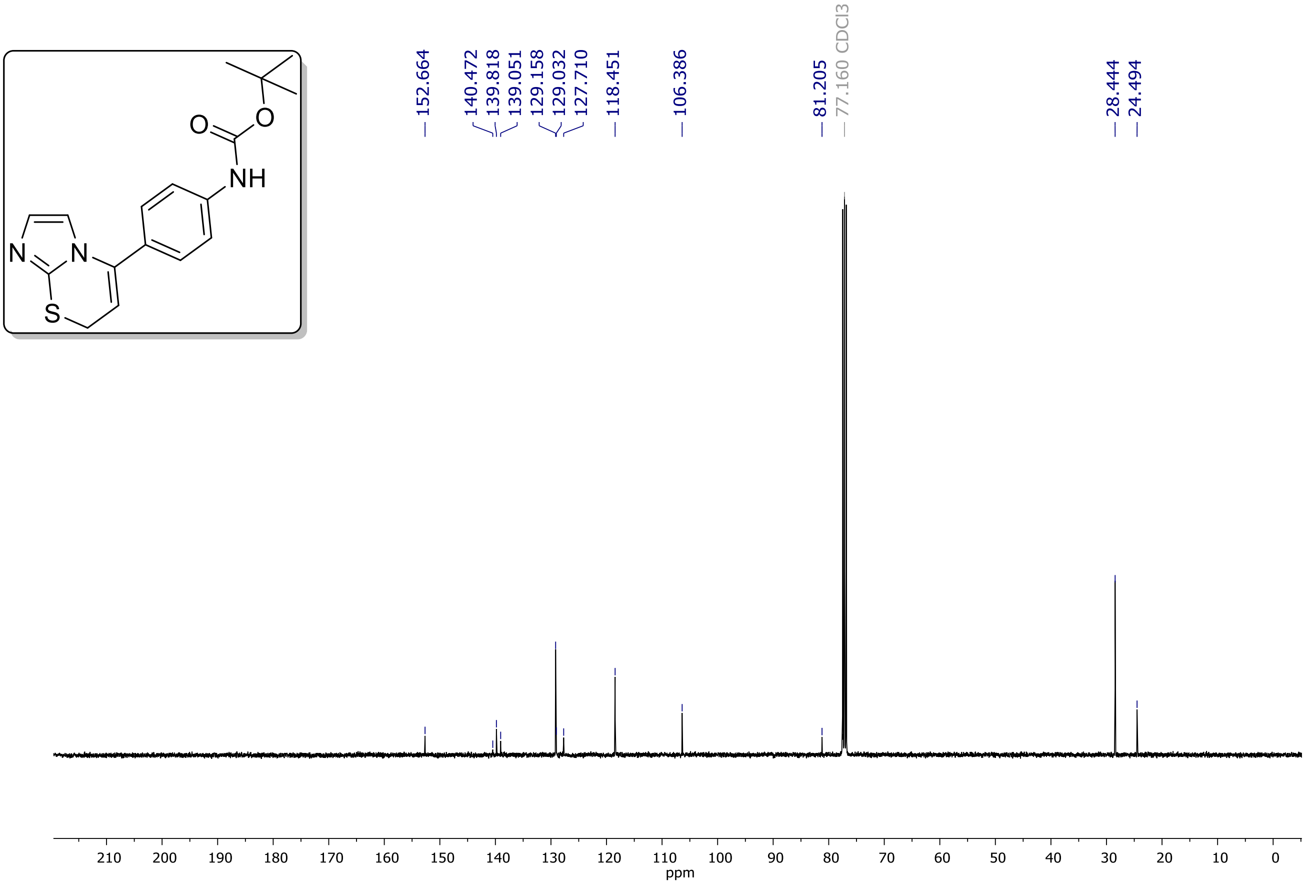

Figure S33. ^{13}C NMR (100 MHz, $CDCl_3$) spectrum of **2e**.

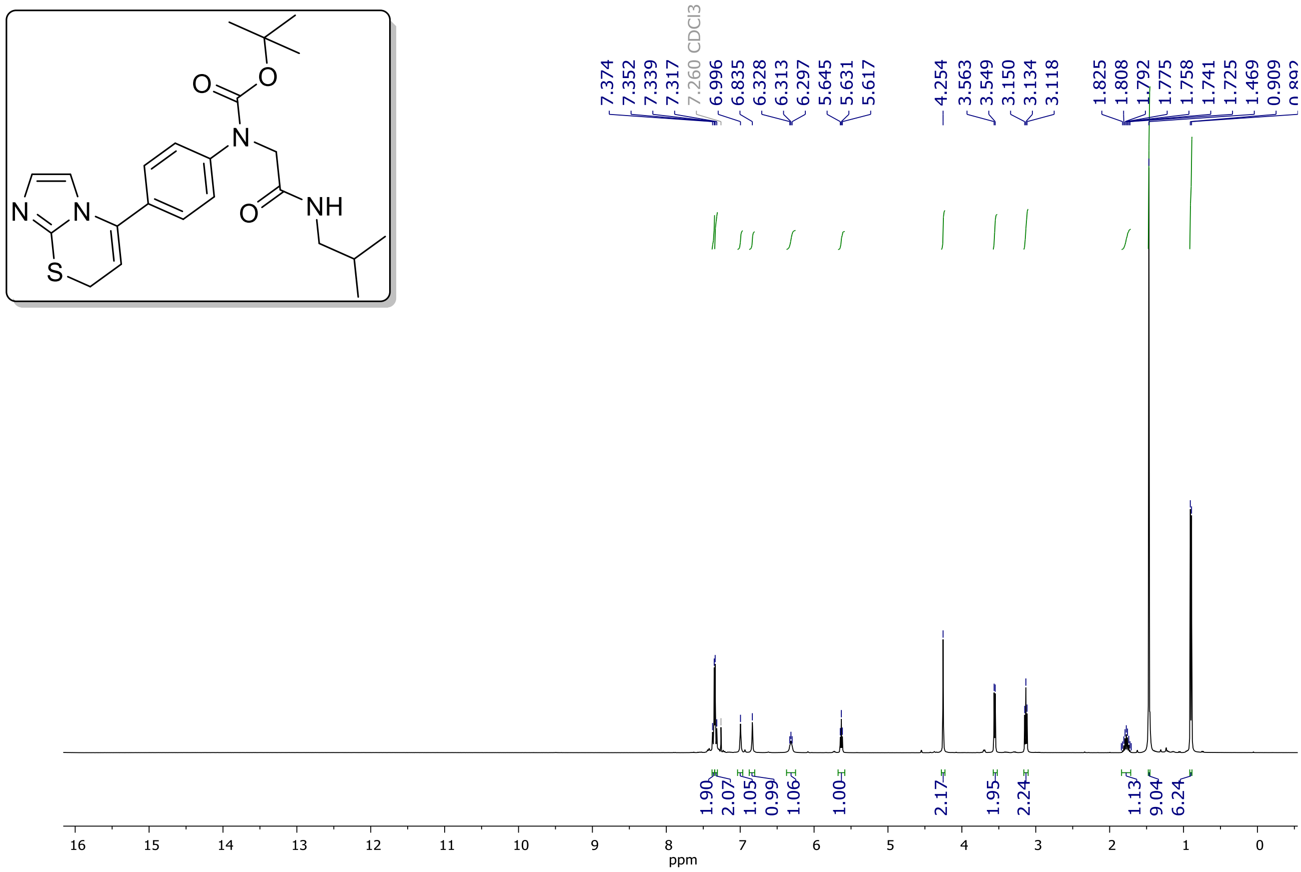

Figure S34. ^{1}H NMR (400 MHz, $CDCl_3$) spectrum of **2f**.

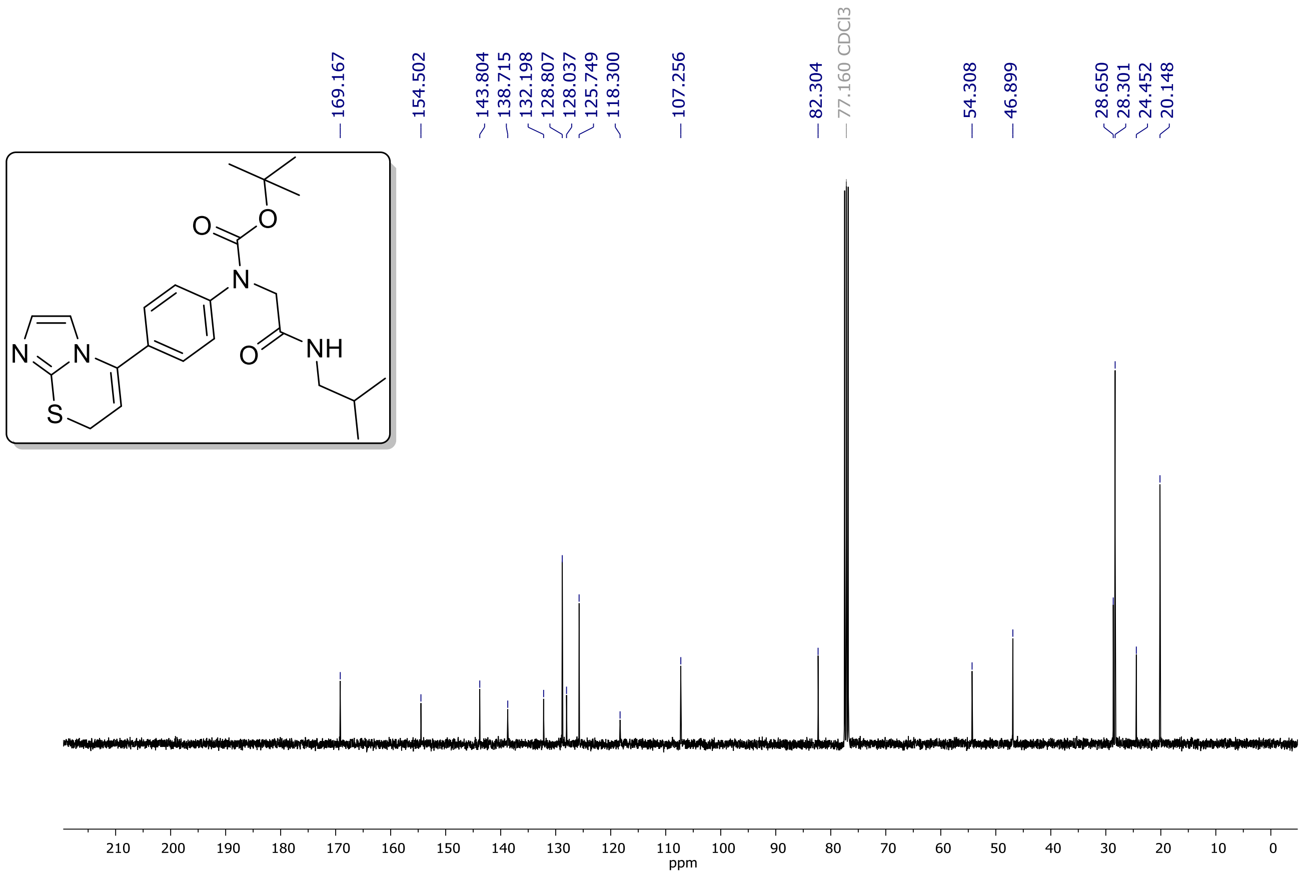

Figure S35. ^{13}C NMR (100 MHz, $CDCl_3$) spectrum of **2f**.

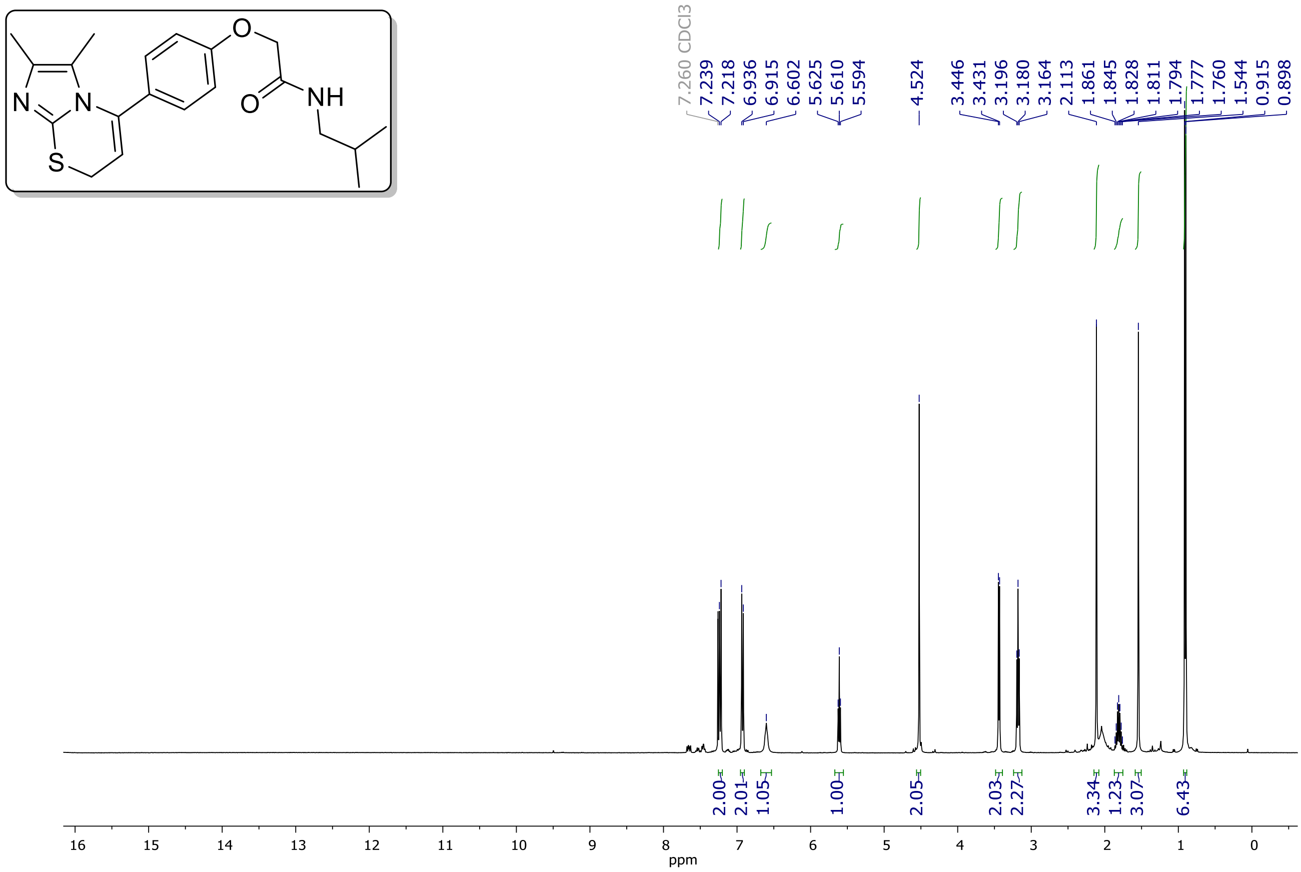

Figure S36. ^{1}H NMR (400 MHz, $CDCl_3$) spectrum of **2h**.

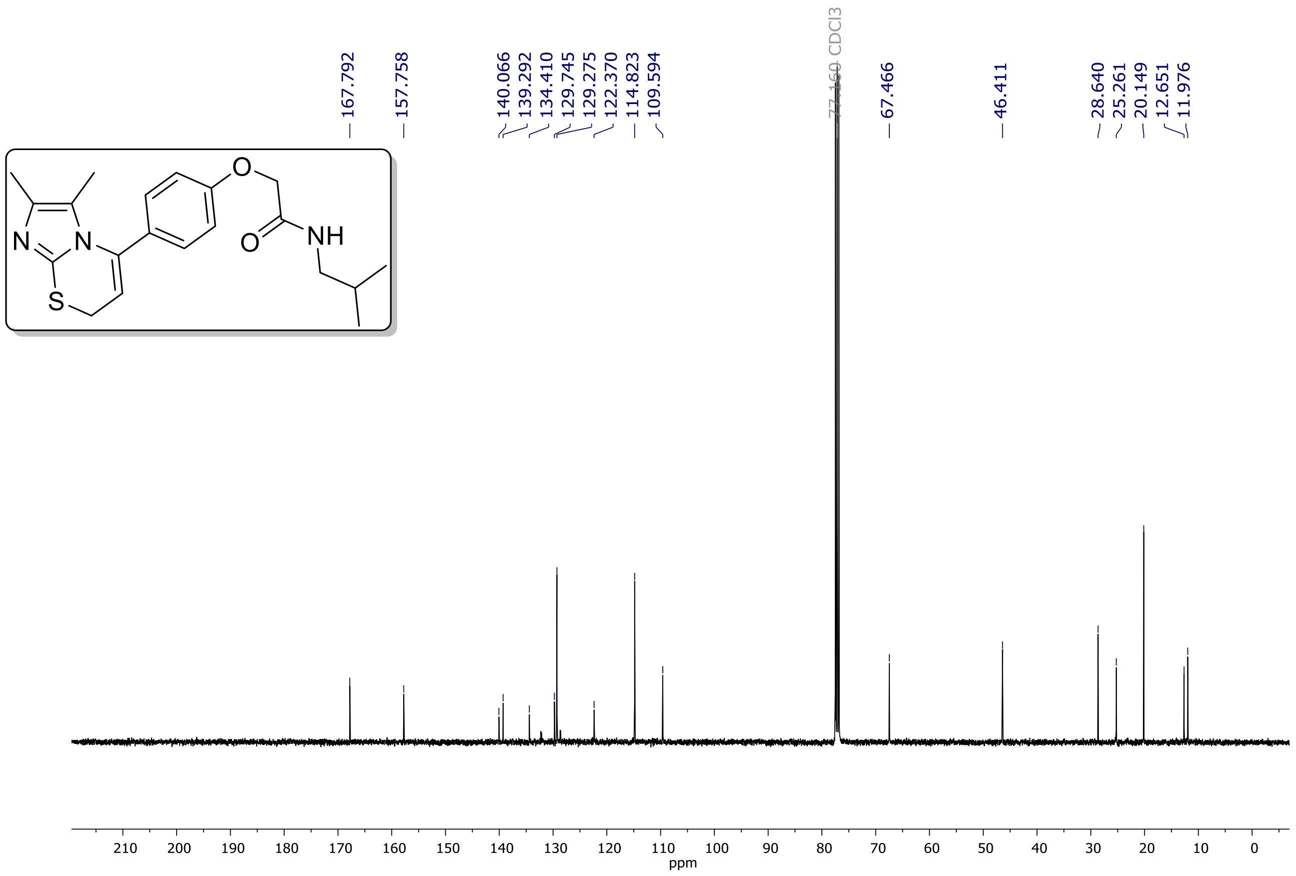

Figure S37. ^{13}C NMR (100 MHz, $CDCl_3$) spectrum of **2h**.

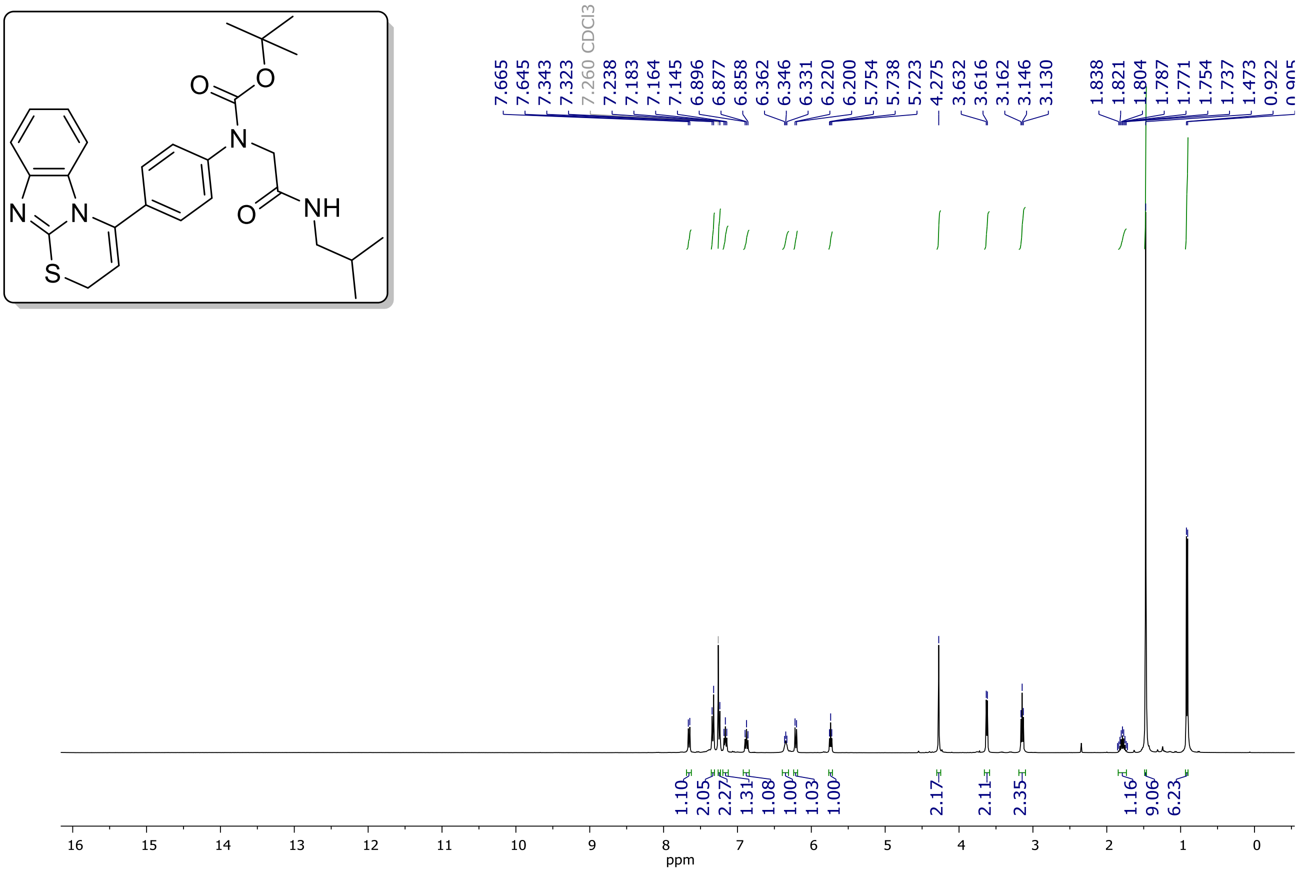

Figure S38. ^{1}H NMR (400 MHz, $CDCl_3$) spectrum of **2j**.

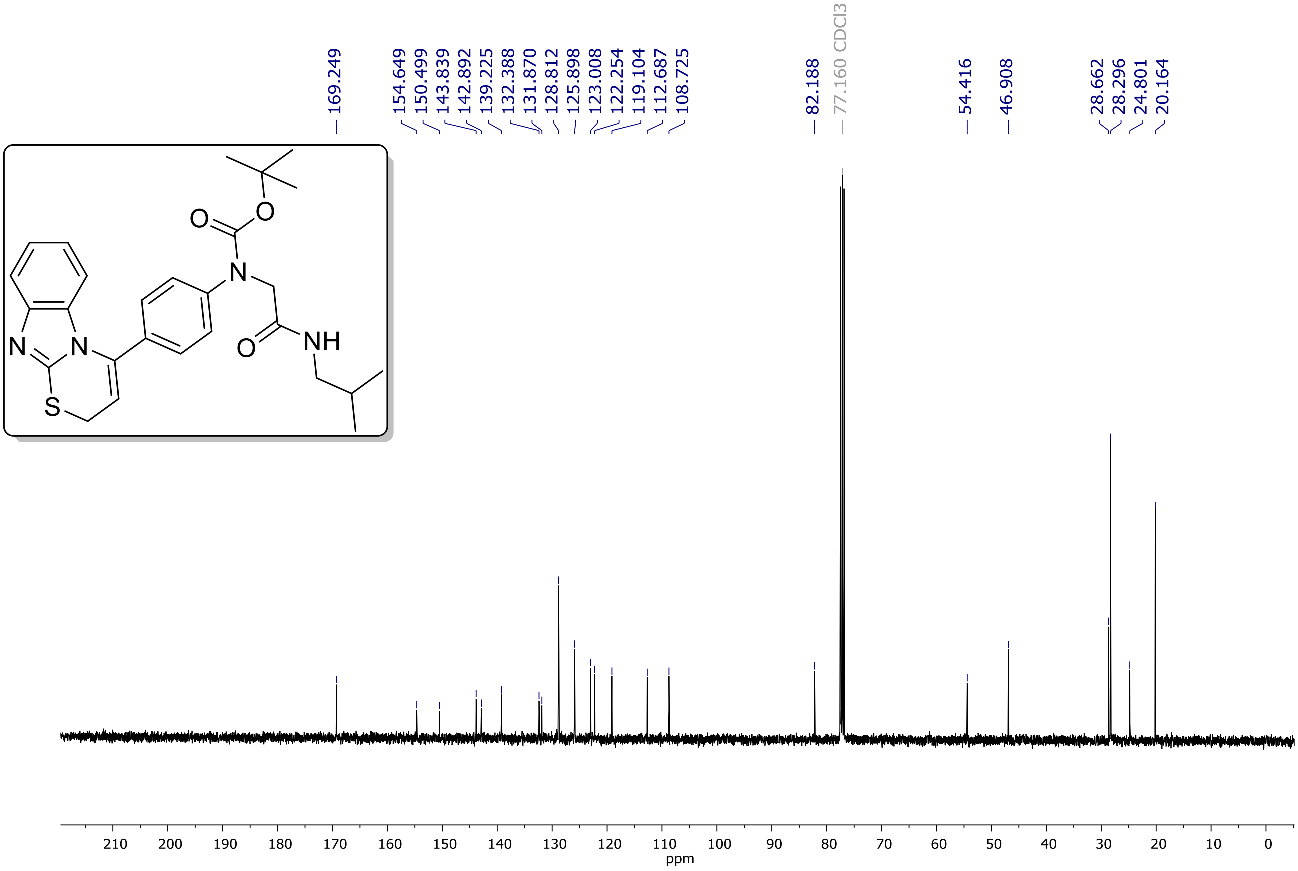

Figure S39. ^{13}C NMR (100 MHz, $CDCl_3$) spectrum of **2j**.

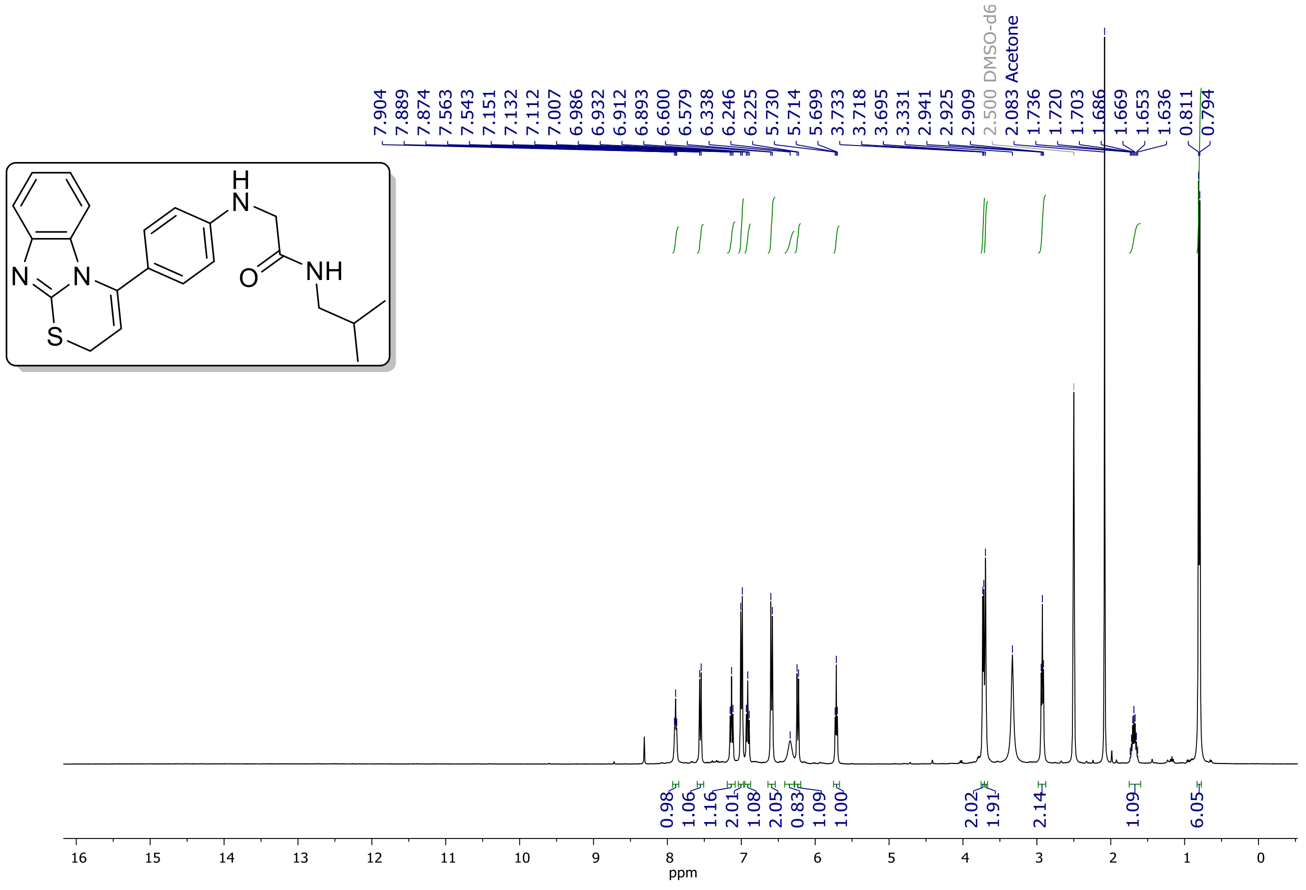

Figure S40. ^{1}H NMR (400 MHz, DMSO-d_6) spectrum of **3**.

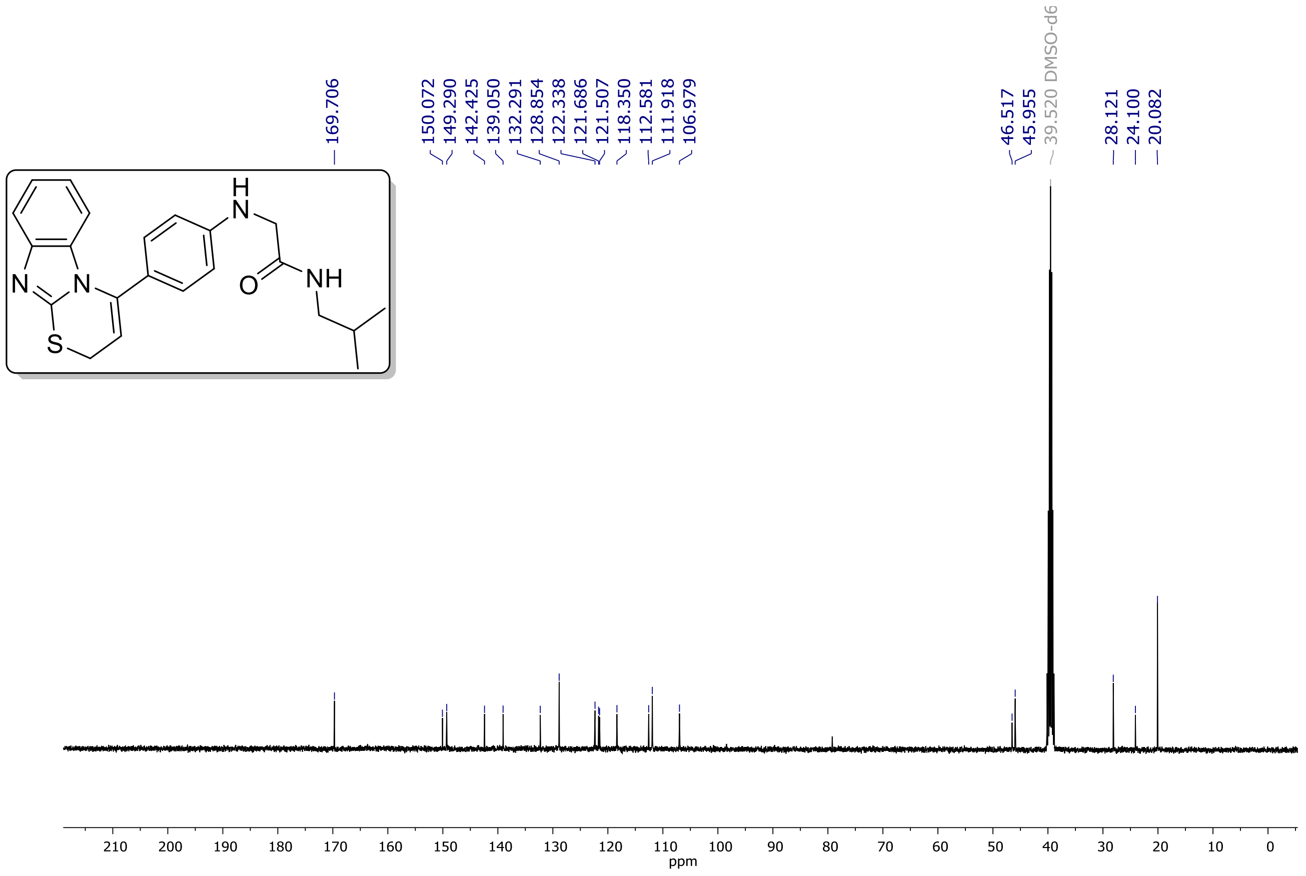

Figure S41. ^{13}C NMR (100 MHz, DMSO-d_6) spectrum of **3**.

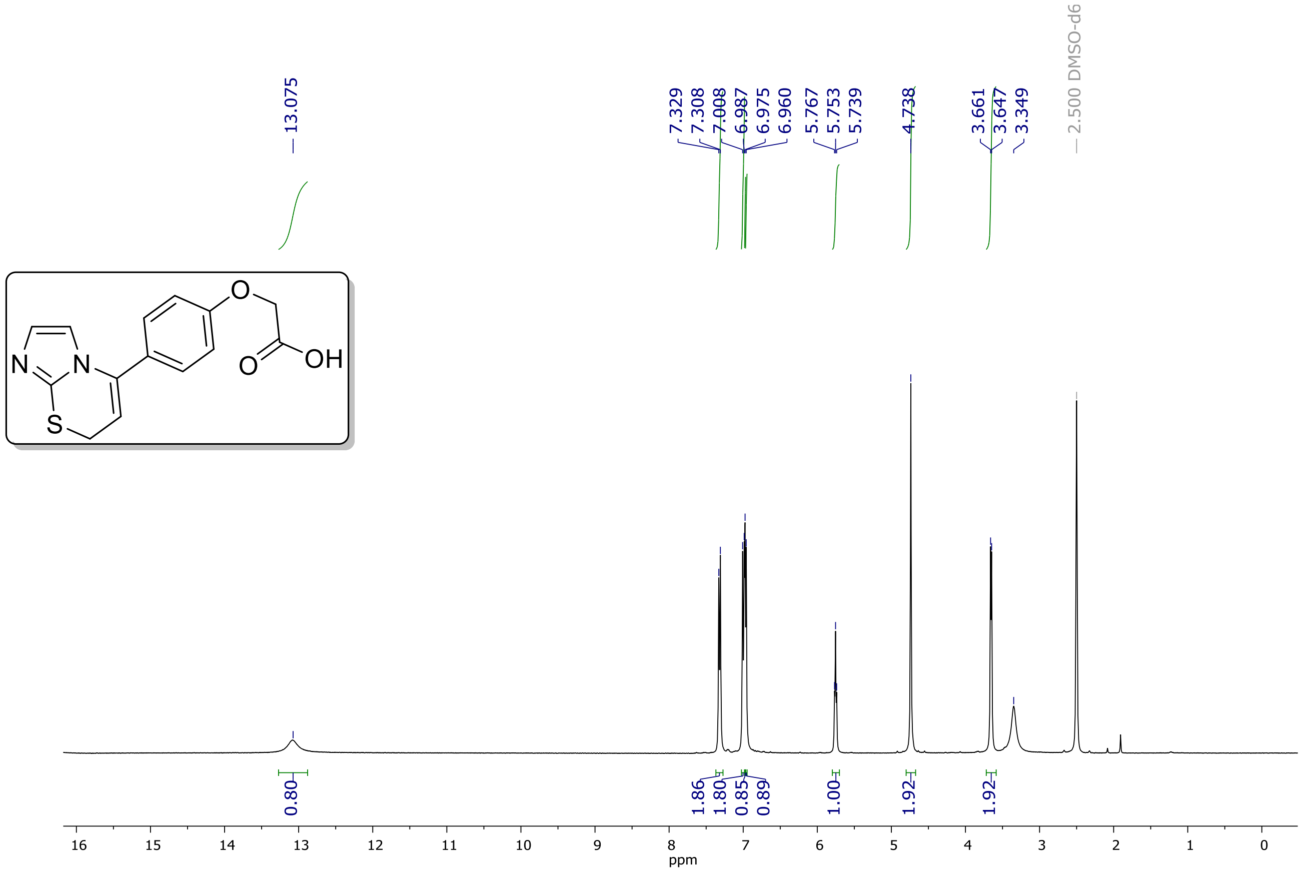

Figure S42. ^{1}H NMR (400 MHz, DMSO-d_6) spectrum of **4**.

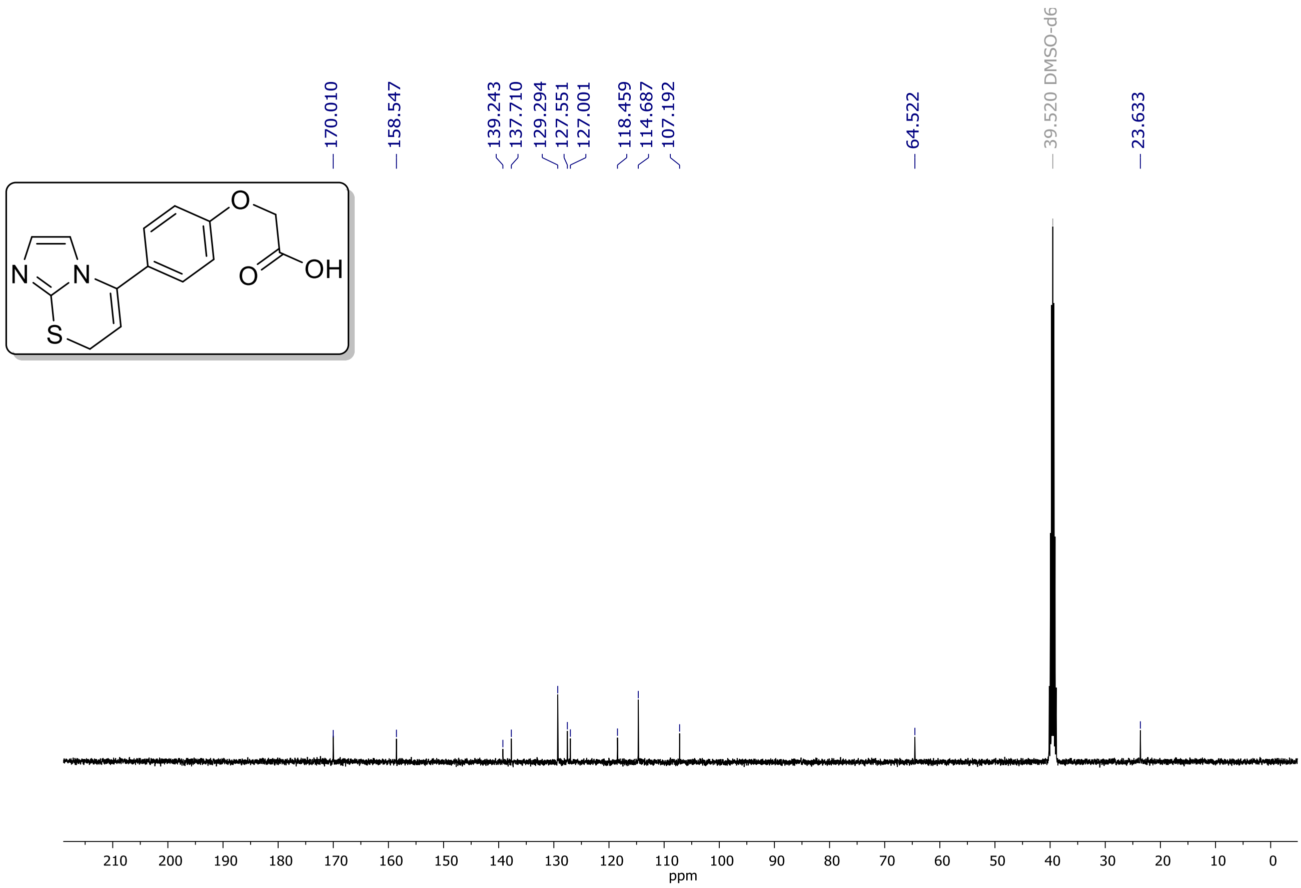

Figure S43. ^{13}C NMR (100 MHz, DMSO-d_6) spectrum of **4**.

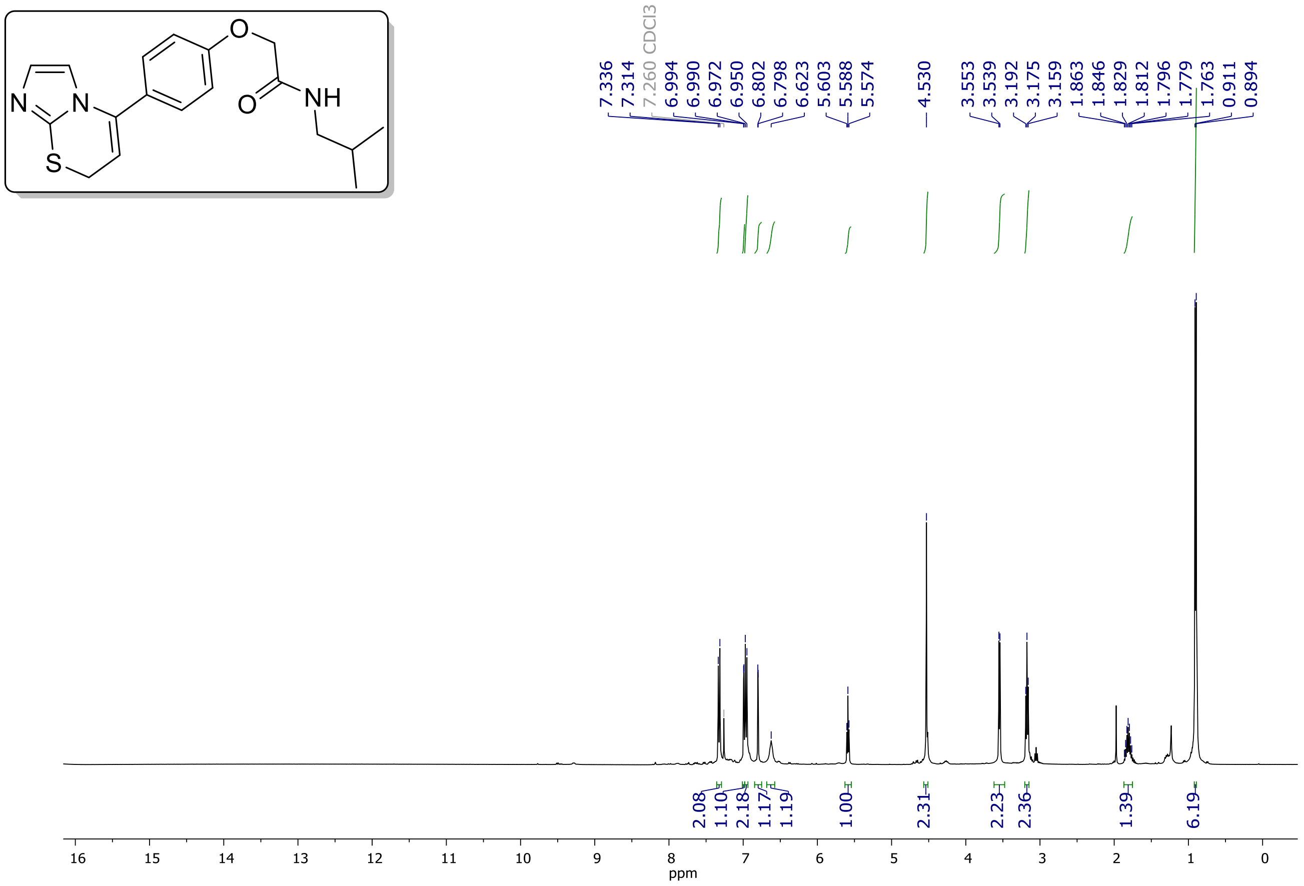

Figure S44. ^{1}H NMR (400 MHz, $CDCl_3$) spectrum of **5a**.

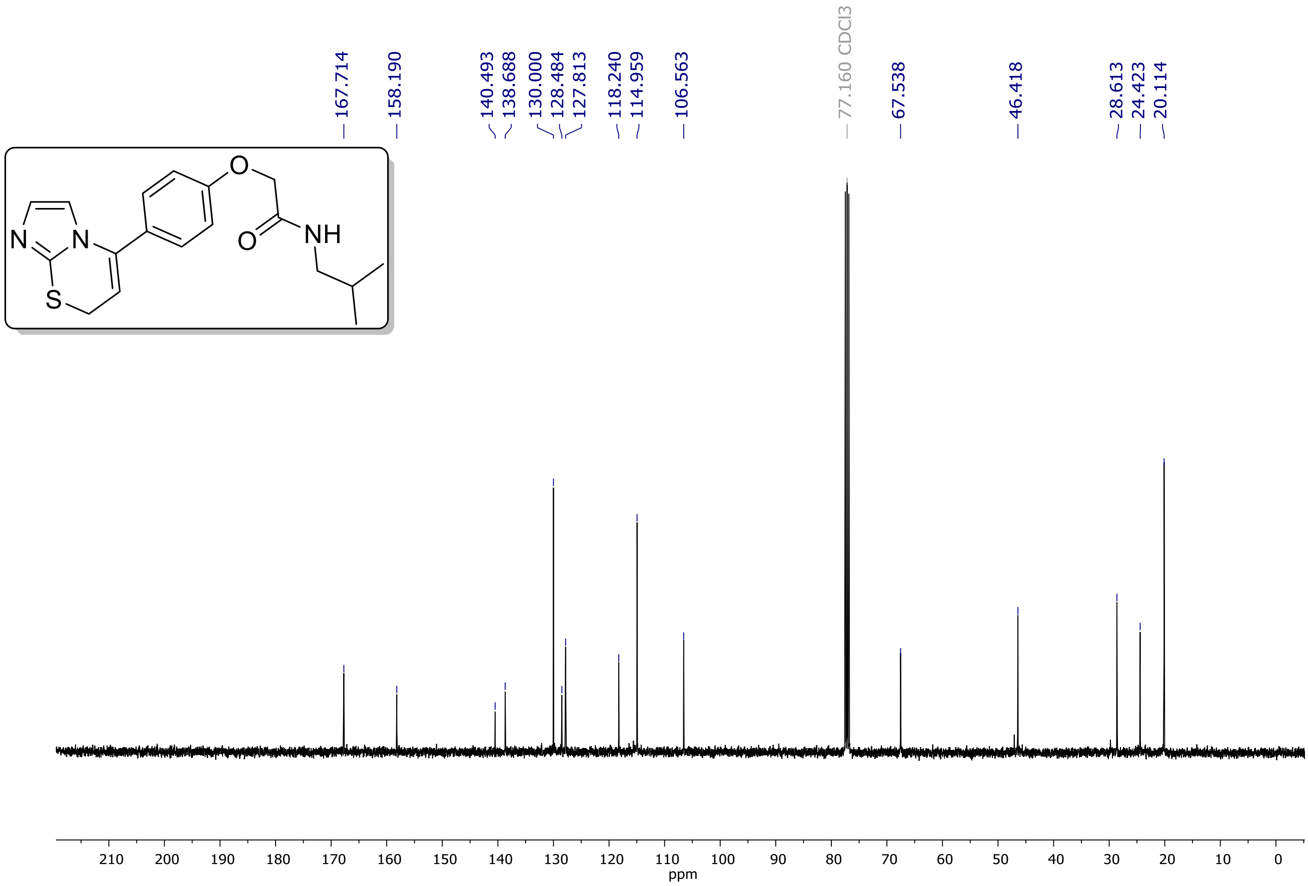

Figure S45. ^{13}C NMR (100 MHz, $CDCl_3$) spectrum of **5a**.

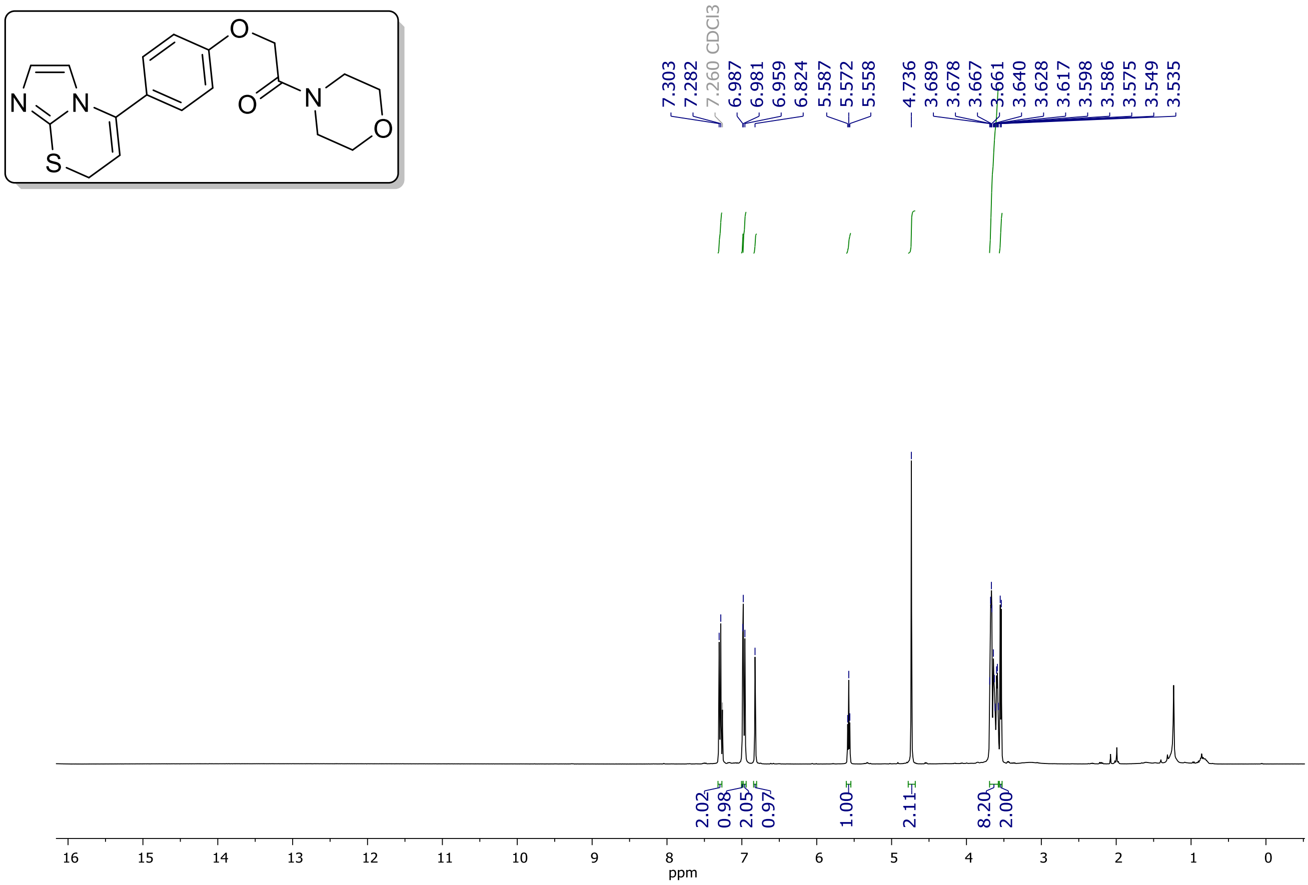

Figure S46. ^{1}H NMR (400 MHz, $CDCl_3$) spectrum of **5b**.

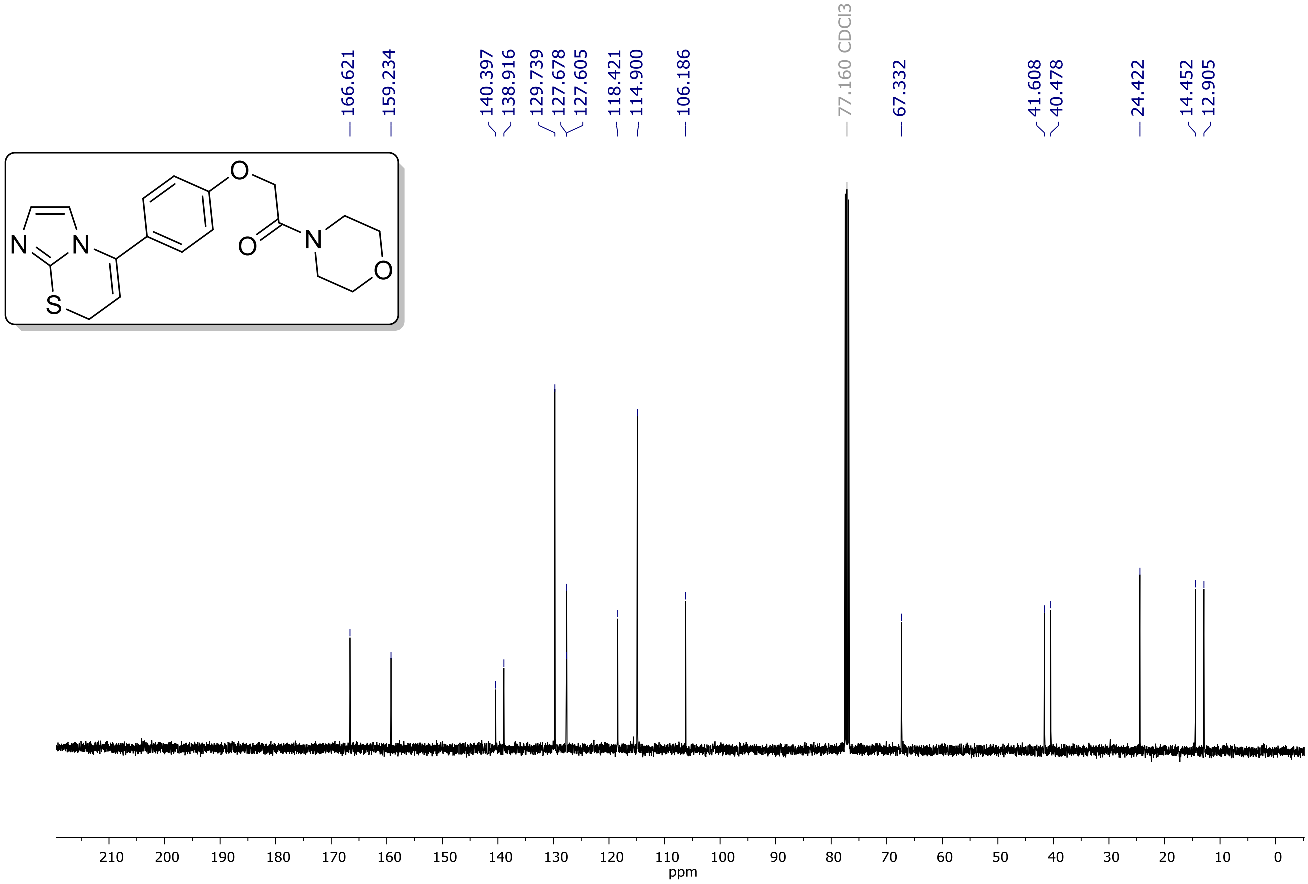

Figure S47. ^{13}C NMR (100 MHz, $CDCl_3$) spectrum of **5b**.

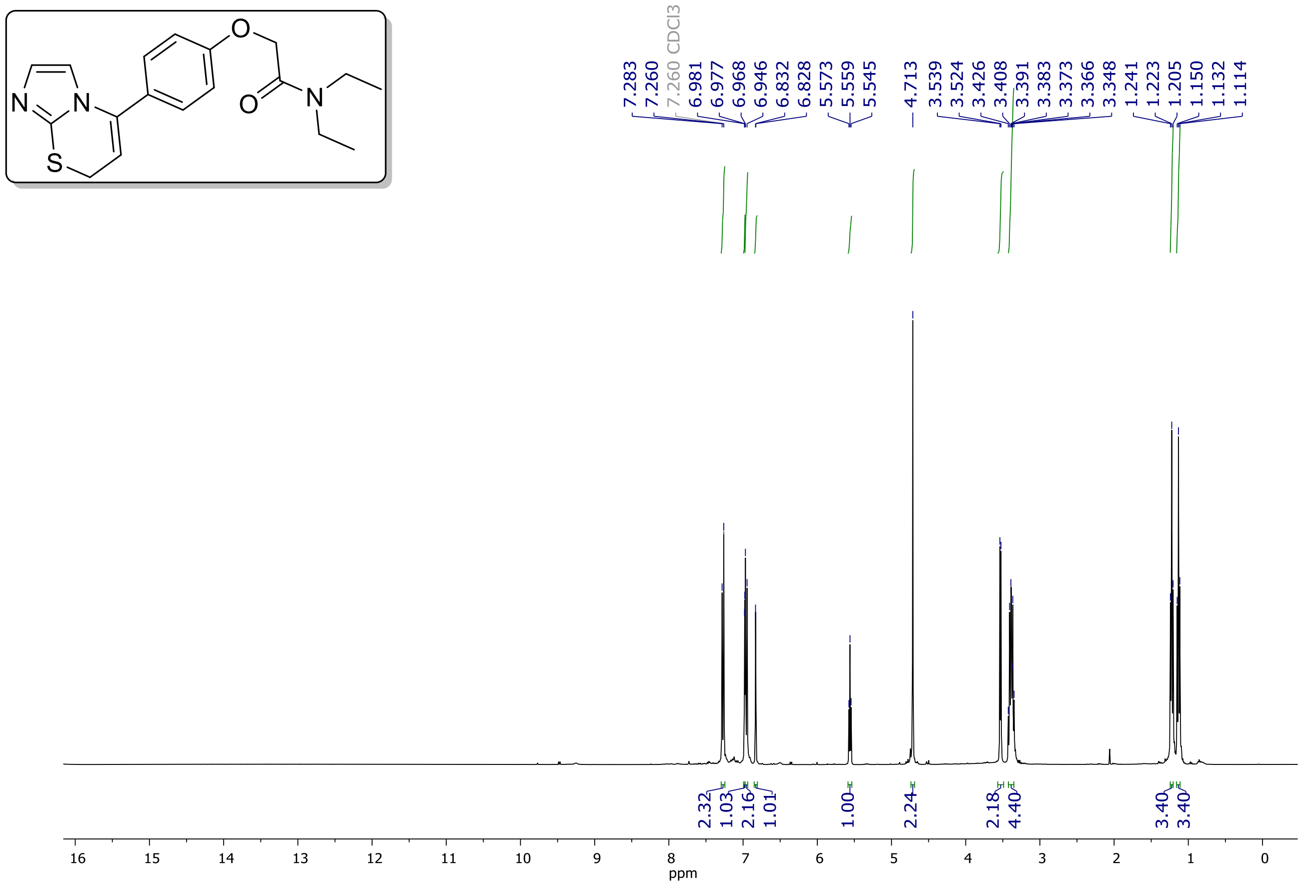

Figure S48. ^{1}H NMR (400 MHz, $CDCl_3$) spectrum of **5c**.

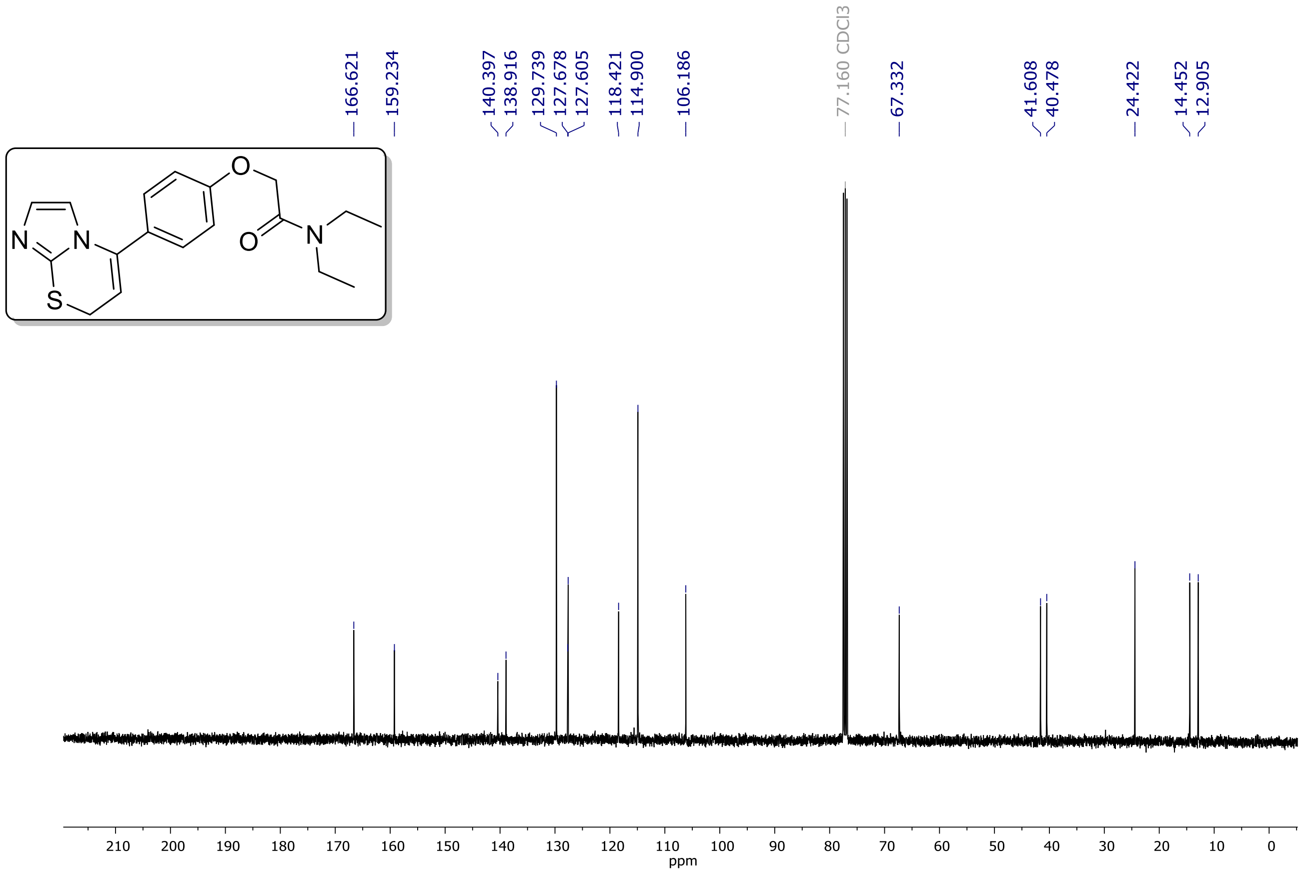

Figure S49. ^{13}C NMR (100 MHz, $CDCl_3$) spectrum of **5c**.

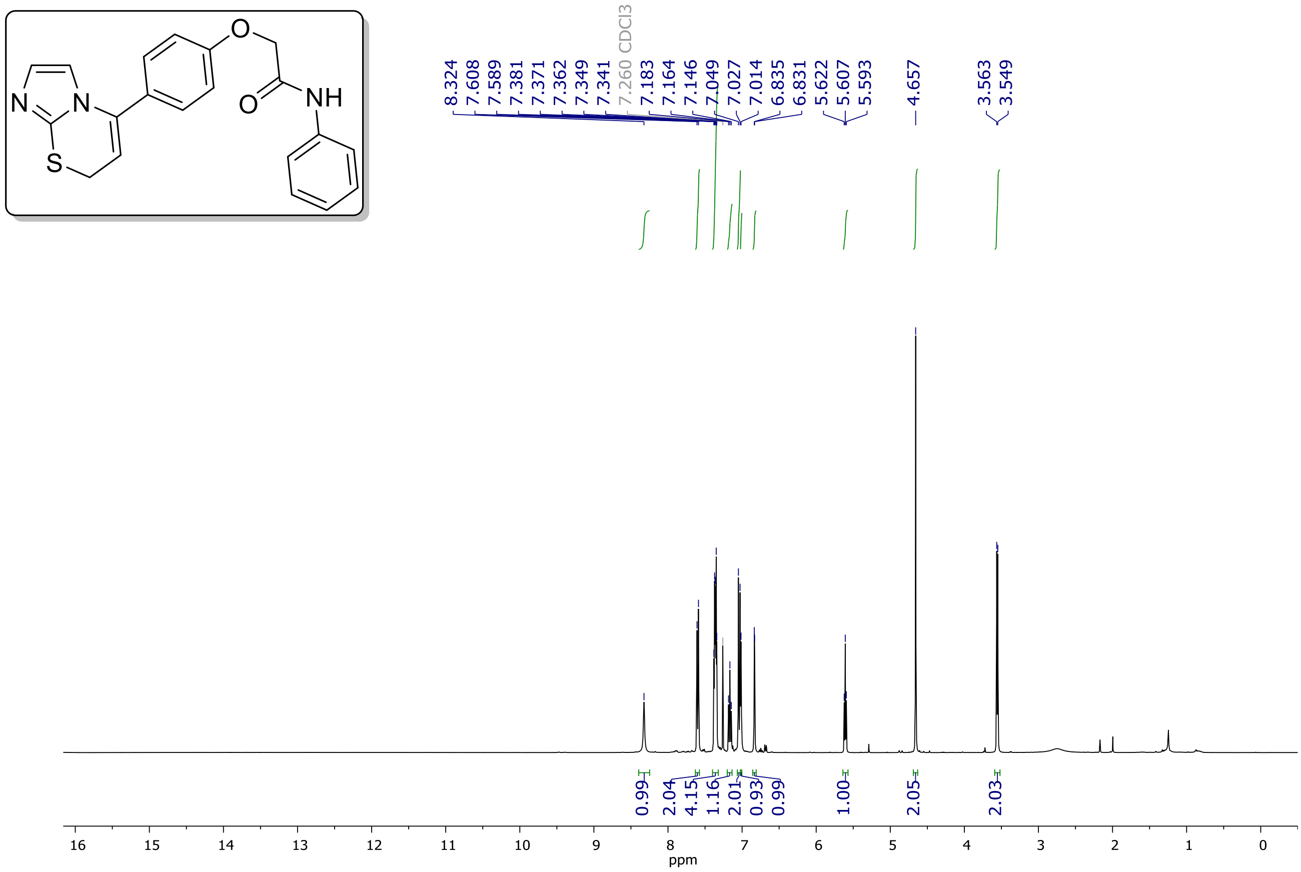

Figure S50. ^{1}H NMR (400 MHz, $CDCl_3$) spectrum of **5d**.

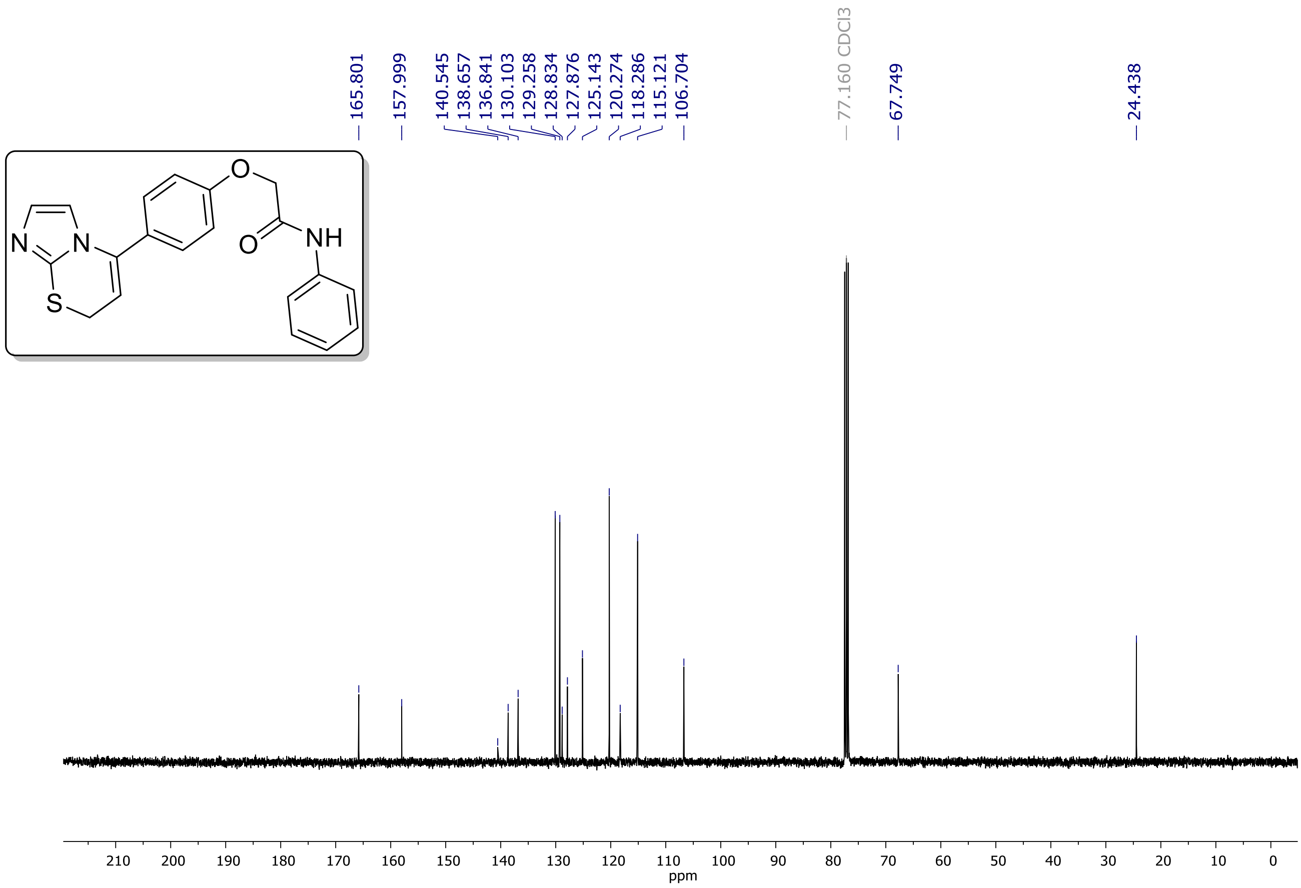

Figure S51. ^{13}C NMR (100 MHz, $CDCl_3$) spectrum of **5d**.

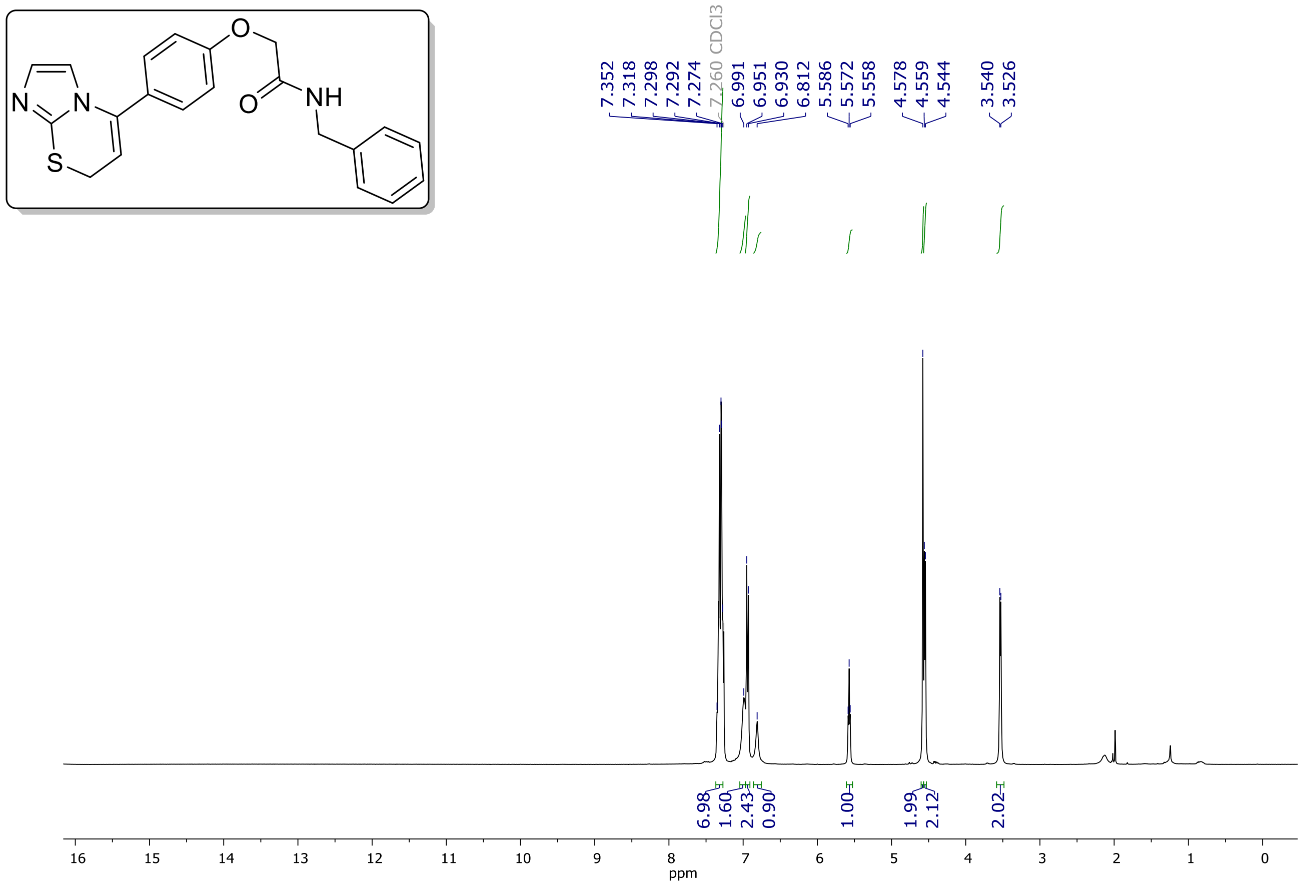

Figure S52. ^{1}H NMR (400 MHz, $CDCl_3$) spectrum of **5e**.

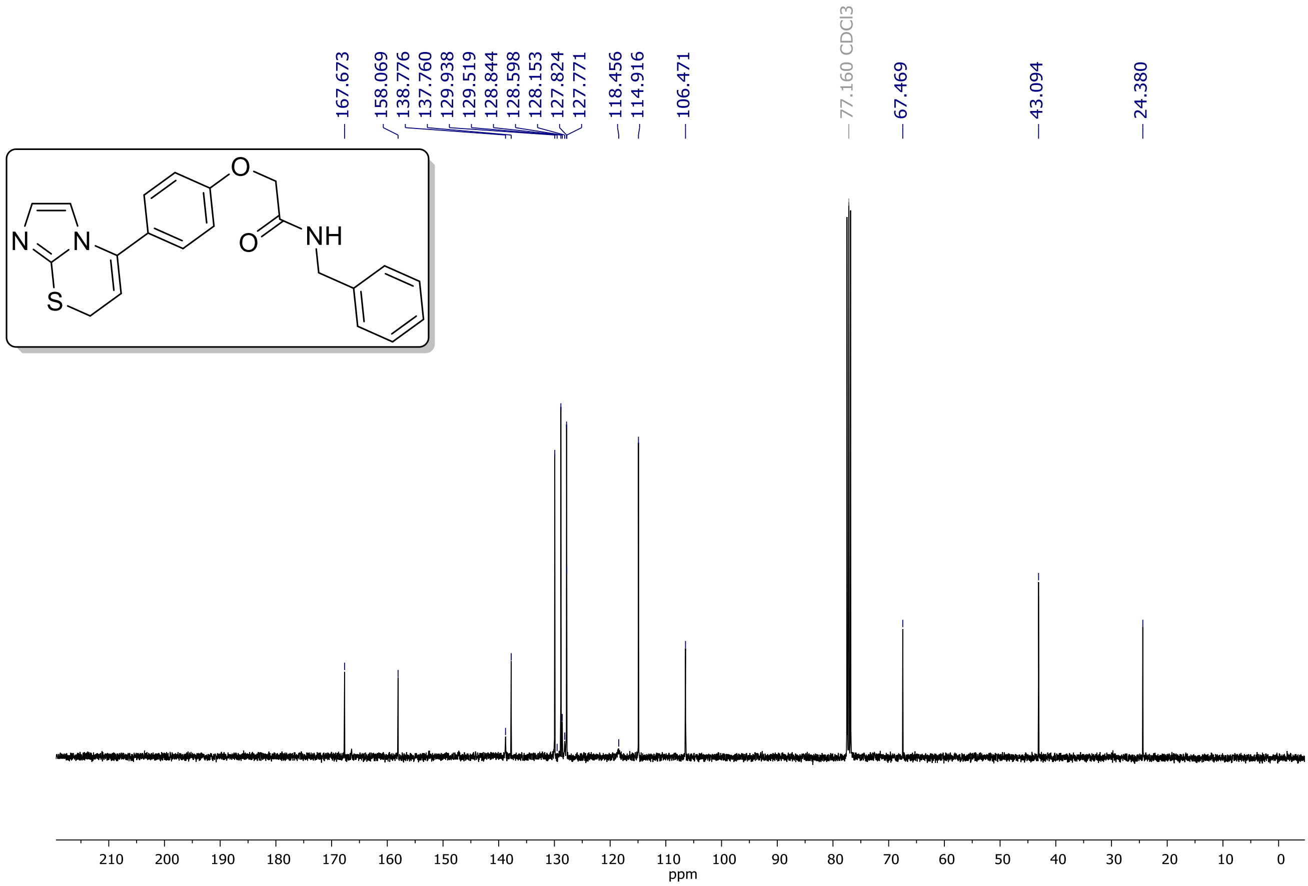

Figure S53. ^{13}C NMR (100 MHz, $CDCl_3$) spectrum of **5e**.

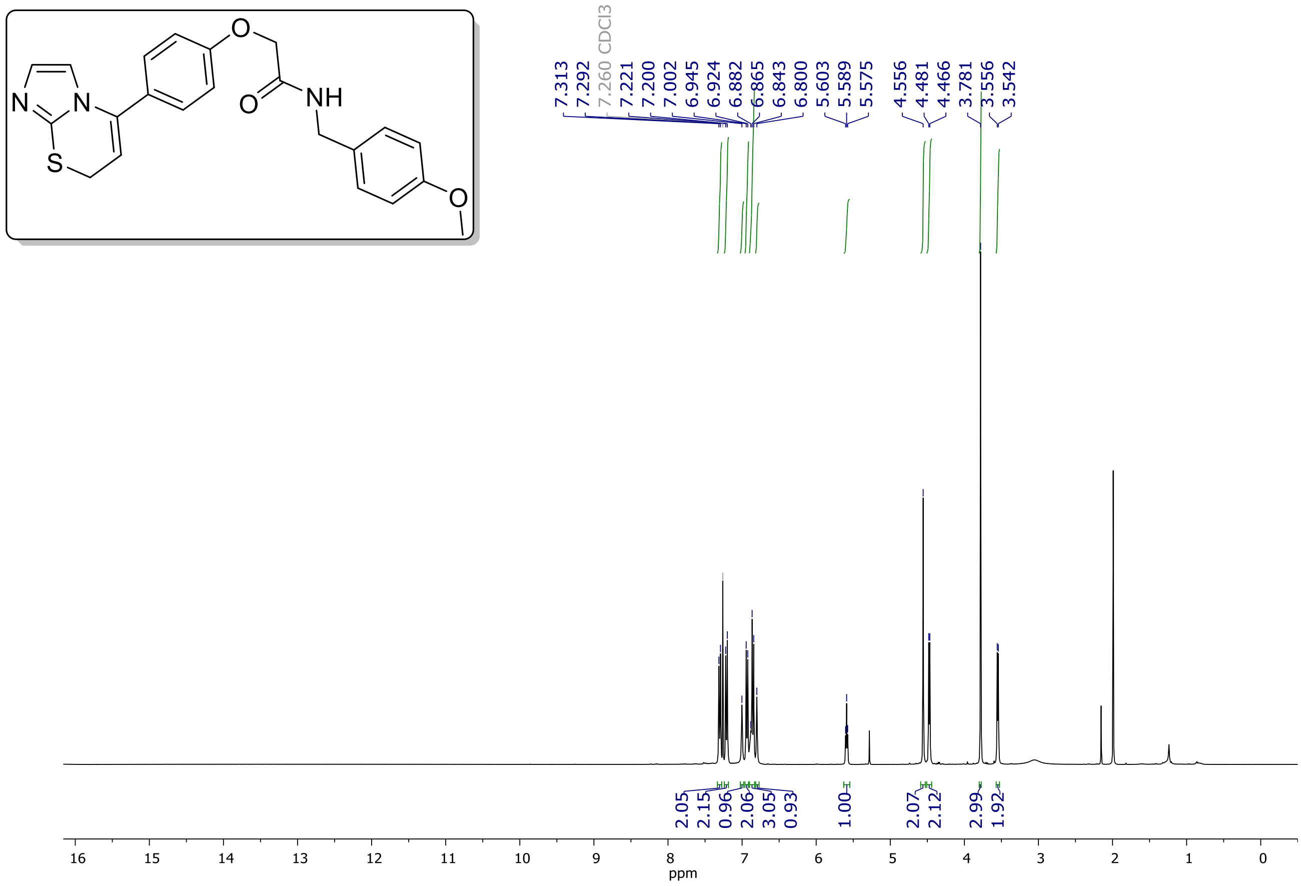

Figure S54. ^{1}H NMR (400 MHz, $CDCl_3$) spectrum of **5f**.

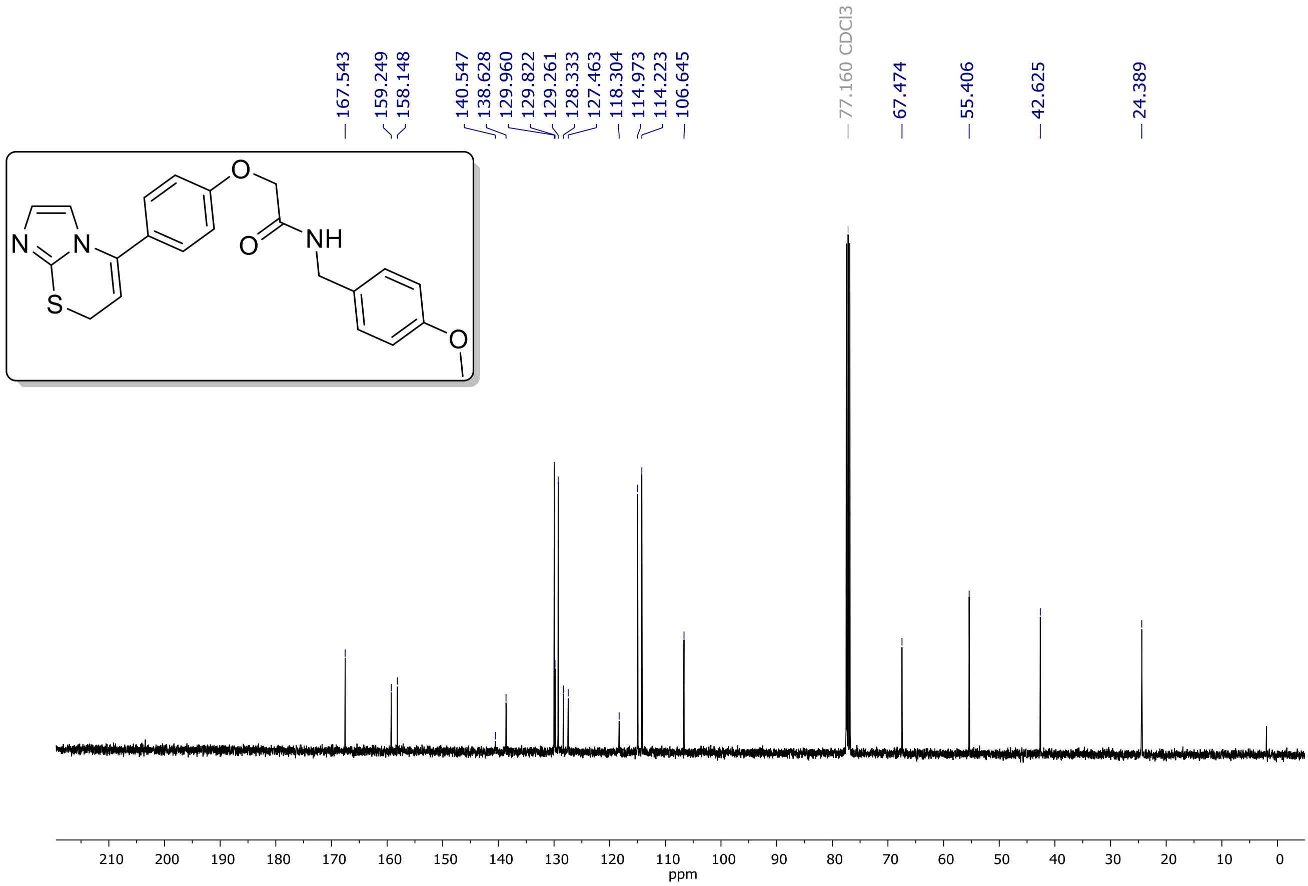

Figure S55. ^{13}C NMR (100 MHz, $CDCl_3$) spectrum of **5f**.

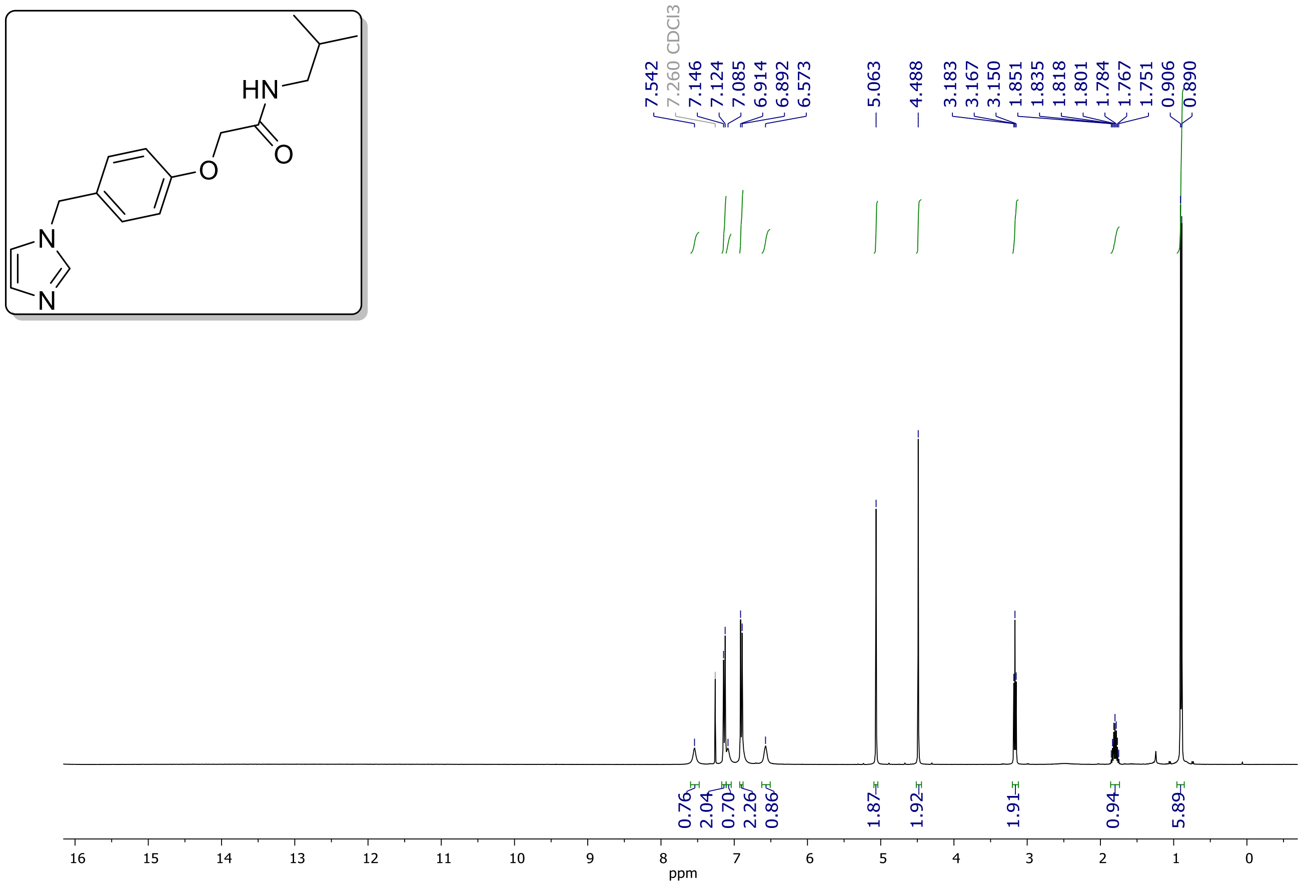

Figure S56. ^{1}H NMR (400 MHz, $CDCl_3$) spectrum of **6**.

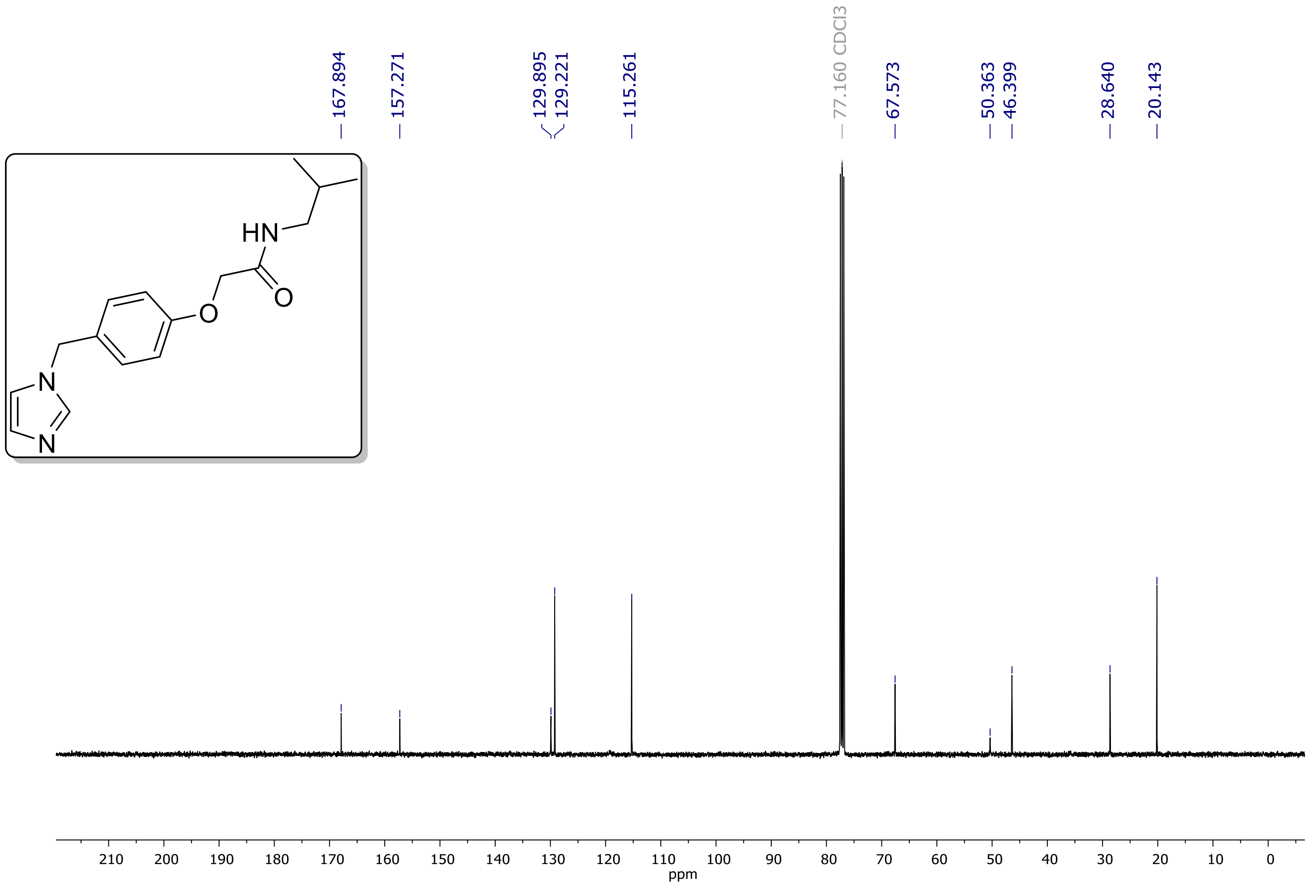

Figure S57. ^{13}C NMR (100 MHz, $CDCl_3$) spectrum of **6**.

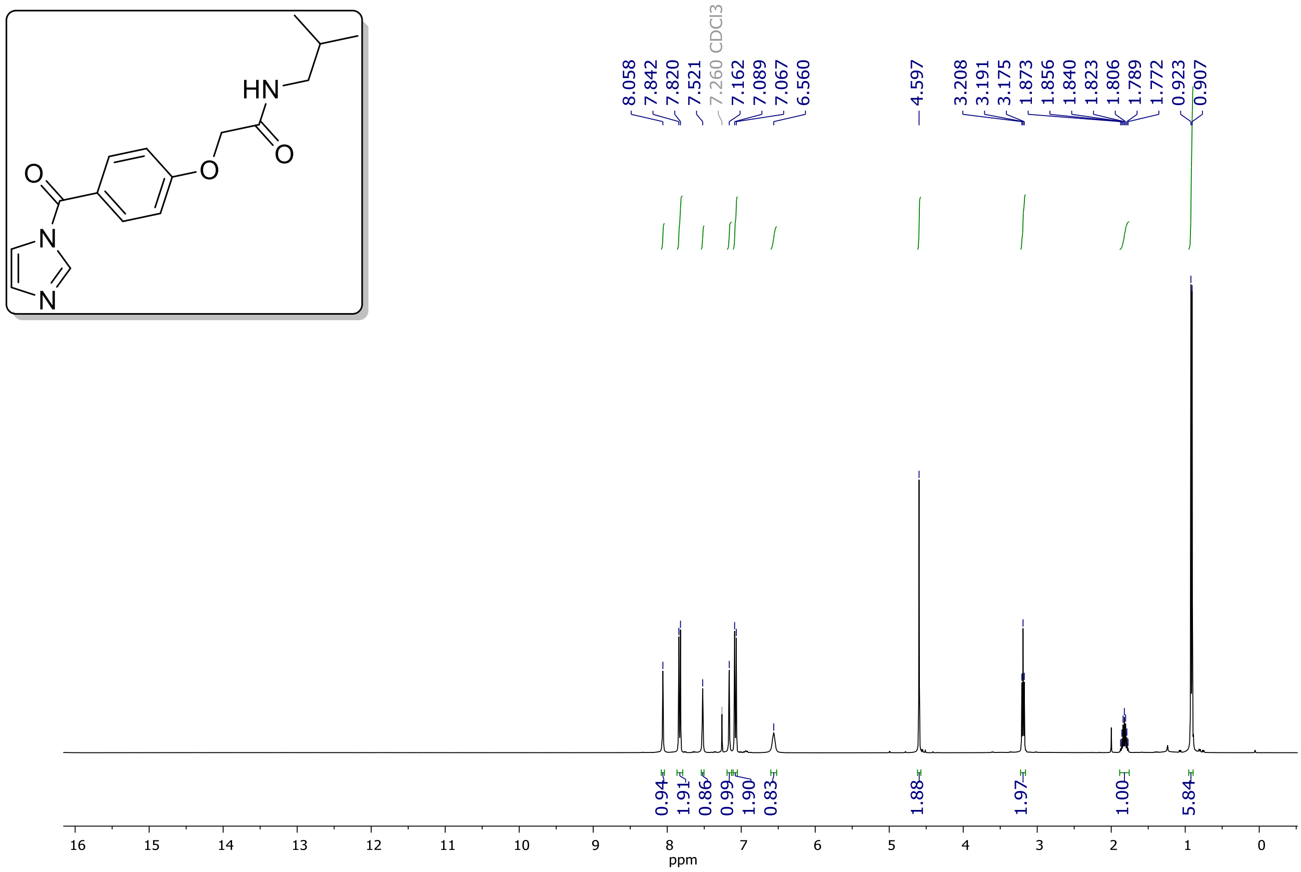

Figure S58. ^{1}H NMR (400 MHz, $CDCl_3$) spectrum of **7**.

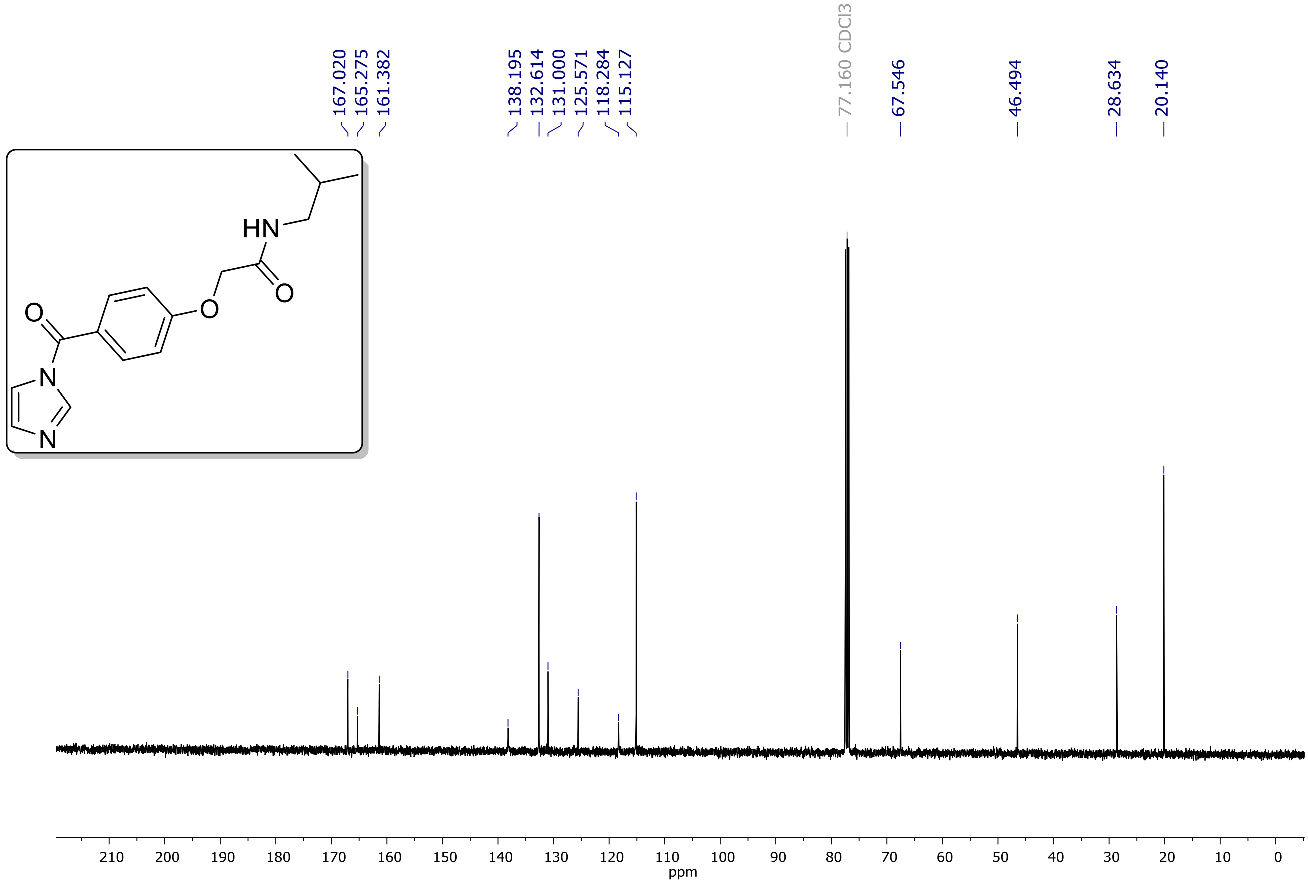

Figure S59. ^{13}C NMR (100 MHz, $CDCl_3$) spectrum of **7**.
